# The Impact of Long–Term Orientation Traits on Pandemic Fatigue Behavior: Evidence from the Columbian Exchange

**DOI:** 10.1007/s10887-022-09218-0

**Published:** 2022-10-27

**Authors:** Sutanuka Roy, Sudhir Gupta, Rabee Tourky

**Affiliations:** Research School of Economics, Australian National University, Canberra, Australia

**Keywords:** Long-term orientation traits, Delayed gratification, Agricultural origins of preferences, COVID-19 pandemic fatigue behavior, D90, I12, O10, O11, O33, O40, Z1

## Abstract

Leveraging exogenous variation in time preferences, we measure the causal effects of culturally embodied long-term orientation traits on voluntary social distancing behavior, COVID-19 cases, hospitalizations, and mortality outcomes in 2020 in the United States. We establish that long-term orientation traits with bio-geographical origins causally reduce measures of COVID-19 cases, deaths and hospitalization, inpatient bed utilization, and age-specific excess deaths. Mobility indicators measuring voluntary decisions to socially distance, comprising measures of visitors/visits to recreational locations, and mobility proxy measuring duration of hours away from home show that a lower prevalence of long-term orientation traits explains persistent resistance to social distancing.

**Supplementary Information:**

The online version contains supplementary material available at 10.1007/s10887-022-09218-0.

## Introduction

Preferences with deep historical origins diverge substantially across regions (Galor & Özak, 2016; Becker et al., 2020). There are convincing theoretical arguments (Galor & Moav, 2002; Galor & Michalopoulos, 2012), as well as empirical evidence,^1^ that human traits that are deeply rooted in long-term history—persisting over time, and intergenerationally transmitted—are key drivers of contemporary economic outcomes; including per capita income, entrepreneurial activity, labor force participation, frequency of armed conflict, and the process of economic development. In this paper, we examine the role of culturally embodied time preference traits that have been transmitted across generations in explaining variations in the impact of the ongoing COVID-19 pandemic.

The success of policy aimed at containing the pandemic depends, in part, on sustained voluntary adherence to guidelines, such as social distancing rules (Monod et al., 2021). Thus, the notion of pandemic fatigue—reduction in individual efforts over time to comply with recommended protective behaviors WHO (2020)—has been a recurrent theme (Crane et al., 2021). It is possible that the behavior consistent with pandemic fatigue is driven by, *a priori* given, time preference traits associated with a lower disposition to exert self-control or delay gratification, which are known to impact behavior in health-related contexts.^2^ We propose and study the hypothesis that long-term orientation traits (LTO)—the ability to sacrifice the present for future rewards, indicative of a future-oriented mindset^3^—causes sustained adherence to mobility restrictions, resulting in reduced COVID-19 incidence, hospitalizations, and deaths during protracted pandemic conditions.

Assessing the causal impact of time preferences on mobility and disease severity during the pandemic is an empirically challenging task. Observed regional variation in time preferences may be correlated with COVID-19 policies or unobservable contemporary economic and cultural factors. To identify the effect of time preferences, an exogenous variation in observed preferences is required. In this paper, we leverage the bio-geographical origins of contemporary spatial variation in time preferences identified by Galor and Özak (2016), where time preference is measured by the index of LTO traits (Hofstede, 1991). Using a natural experiment associated with an expansion of suitable crops for cultivation during the Columbian Exchange, Galor and Özak (2016) theoretically and empirically establish that preindustrial agro-climatic conditions, which were instrumental for a higher return on agricultural investment, initiated the processes of adaptation, learning, and intergenerational transmission that resulted in a higher prevalence of LTO traits in the modern period.

Moreover, Galor and Özak (2016) show that societies in which the ancestral population was exposed to a higher preindustrial caloric potential yield (for a given crop growth cycle) and a higher post-1500 CE potential yield change during the course of the Columbian Exchange (given pre-1500 CE levels) have a higher representation of traits for LTO in the contemporary period. They establish that the potential crop yield experienced by the ancestors of today’s population has had a direct causal effect on LTO rather than an indirect effect mediated by economic development processes or various other measures of cultural characteristics. This allows us, in this paper, to associate a causal interpretation with the reduced-form effect of the preindustrial potential crop yield experienced by the ancestors of the current population as a proxy for contemporary, culturally embodied LTO traits on mobility and COVID-19 severity during the pandemic.

Our study involves county-level observations in the US, a center of the COVID-19 pandemic with confirmed cases exceeding 13,000,000 and more than 266,000 deaths by November 2020, and with substantial variation in pandemic severity (Desmet & Wacziarg, 2021) and significant economic impact (Chetty et al., 2020).^4^ In the US context, a large fraction of the current population is descended from people who lived in Europe, Africa, or other countries in 1500 (Putterman & Weil, 2010), thereby generating a mismatch between the preindustrial agro-climatic characteristics of the territory and those experienced by the ancestors of contemporary US residents. Therefore, to estimate the effect of culturally embodied ancestral LTO on current pandemic behavior and disease severity in the US, we would need to correct the agricultural proxies for ancestry in 1500 CE. Even after correcting for the location of the 1500 CE ancestors of current county residents, the ancestry-adjusted crop yield measures will have endogeneity concerns due to possible selective historical migrations from origin countries to destination counties or to selective reporting of ancestry due to present or past economic conditions. To correct for ancestry in 1500 CE and address endogeneity concerns, we apply the set of instrumental variables (IVs) for the present-day ancestry composition of US counties developed in Burchardi et al. (2019) to the method used in Galor and Özak (2016).

Our empirical strategies use variation in primary measures of culturally embodied LTO traits established in Galor and Özak (2016): (1) the ancestry-adjusted pre-1500 CE potential crop yield and (2) the ancestry-adjusted post-1500 CE potential yield change during the Columbian Exchange. The first identification strategy uses cross-sectional county-level variation in LTO proxies. The second identification strategy uses event study specifications with county fixed effects and time-varying controls to identify differential trends in pandemic severity and social distancing across high and low LTO counties around the declaration of a national emergency in response to the pandemic on March 13, 2020.

The cross-sectional and dynamic county-level analyses account for potential omitted variable bias concerns^5^ by controlling for various geographic and socio-economic confounders. If there are cross-county population interactions, the county-level estimates may be confounded by the pandemic severity of neighboring counties, potentially violating the identifying assumption—the Stable Unit Treatment Value Assumption (SUTVA) (Rubin, 1980)— and may lead to erroneous inference due to correlated errors. In the county-level analysis, we mitigate the identification concern by controlling for COVID-19 severity and mobility behavior of neighboring counties and address inference concerns by clustering standard errors at the commuting zone (CZ) level, using various methods of inference. To further address the identification and inference issue owing to cross-county population movements, we replicate the whole analysis at the CZ level. In addition, the culturally embodied LTO traits could impact mobility behavior differently across regions even before the COVID-19 crisis. This could result in violation of the parallel trends assumption, which is the key identification assumption in event study specifications. We address this potential issue by using the methodology of Rambachan and Roth (2019) to provide sensitivity analyses for potential violations of the parallel trends assumption.

We find negative economically and statistically significant effects of ancestry-adjusted post-1500 CE potential yield change on measures of COVID-19 cases, deaths, and hospitalization, robust to various methods of inference and empirical strategies. To provide evidence on a broader impact of ancestral LTO traits on the pandemic death toll, we analyze the impact on age-specific excess deaths. In line with the findings on COVID-19 severity measures, we find robust, negative effects of ancestry-adjusted post-1500CE potential yield change on age-specific excess deaths. Consistent with the treatment effects of post-1500CE yield change, ancestry-adjusted pre-1500CE potential crop yield produces a negative effect, but the magnitudes are very large and imprecise. However, after accounting for county fixed effects in event study specifications, we find that both the ancestry-adjusted pre-1500 CE potential yield and the post-1500 CE yield change have economically significant negative effects with similar magnitudes that are highly statistically significant.

The second part of the empirical analysis tests whether cross-county differences in ancestral LTO traits explain cross-county differences in mobility proxies, which have been shown to be the key driver of COVID-19 transmission rates (Nouvellet et al., 2021). Using cross-sectional and event study specifications and cell phone location data on non-essential trips from SafeGraph and Google Community Mobility Reports, two independent sources of mobility data, we show that a lower prevalence of LTO traits explains voluntary resistance to social distancing behavior in the months following the national emergency declaration due to the pandemic. Taken together, our results establish that cultural LTO traits are key drivers of the human behavior affecting coronavirus caseloads and associated deaths. In particular, we show that preindustrial agricultural characteristics, which characterize deep-rooted time preference traits affecting technological adoption, education, and saving (Galor & Özak, 2016), also explain the substantial variation in the pandemic’s impact.

This paper provides the first empirical evidence of the causal effects of time preferences on resistance to social distancing behavior and on the spread of COVID-19 disease and mortality, a key driver of economic development (Lorentzen et al., 2008). While our causal effect estimates add to the recent literature on COVID-19 that examines the correlations between economic preferences and compliance with social regulations in response to COVID-19,^6^ the salience of time preferences during the current global crisis connects to the active literature showing that patience is strongly correlated with per capita income, physical and human capital accumulation and productivity,^7^ and positive personality traits^8^ (Alan & Ertac, 2018). Methodologically, the origins of the distribution of time preferences across countries and regions uncovered in Galor and Özak (2016) make it possible for us to obtain causal estimates of time preferences on pandemic severity. In the US context, where post-1500 CE migration is prevalent, the World Migration Matrix, 1500–2000 developed in Putterman and Weil (2010) and the ancestry IV developed in Burchardi et al. (2019) prove critical in the identification of the effect of ancestral LTO traits.

Our paper also builds on and adds to the long-established economic literature examining the long-term effects of historical factors on contemporary outcomes. In Sect. 2, we explain our contributions to this strand of literature on culture and development that might not be relevant to readers interested in COVID-19. Sections 3 and 4 present the data and empirical strategies. Sections 5 and 6 present impacts on COVID-19 severity and mobility. Section 7 presents the robustness of the event study analysis. Section 8 replicates the analysis at the CZ level, and Sect 9 concludes.

## Relation to literature on the impact of ancestral and historical factors

Our paper is related to the literature that highlights the role of current populations’ history in determining economic outcomes (Putterman & Weil, 2010; Burchardi et al., 2019; Dalgaard et al., 2020), rather than the history of the location where the population currently resides.^9^ The work is closely related to the literature emphasizing the impact of the history of the current populations’ ancestors on norms and beliefs. For instance, Nunn and Wantchekon (2011) show that individuals whose ancestors were heavily raided during the slave trade are less likely to be trusting, and Algan and Cahuc (2010) establish that inherited trust predicts economic productivity. Further, Alesina et al. (2011) test Boserups’ hypothesis by constructing a measure of historic plow use among the ancestors of today’s population and find that societies with a history of plow agriculture feature gender inequality and less female labor force participation. Voigtländer and Voth (2012) explain the local continuity of anti-Semitic beliefs for over 600 years in Germany due to the lack of population mobility, and Ashraf and Galor (2013) empirically establish that prehistoric exodus out of Africa had long-lasting effects on comparative development by affecting population diversity. More recently, Becker et al. (2020) explain population-level differences in economic preferences by the differences in these populations’ historical experiences due to migration in ancient times.

This paper also complements studies in economics that provide evidence for the salience of culture in economic transactions (Guiso et al., 2009); in particular, it is associated with research that examines cultural traits as an important channel through which historical processes affect modern economic performance (Nunn, 2012, 2014). A key interest in cultural economics has been explaining the mechanisms underlying the evolution of culture (Bisin & Verdier, 2011; Doepke & Zilibotti, 2014, 2017). For instance, Giavazzi et al. (2019) theoretically and empirically examine the evolution of a set of cultural traits such as religion, family, gender, sexuality, cooperation, political orientation, and the notion of fairness through processes of parental socialization, termed “vertical transmission,” and socialization outside the family, termed “horizontal transmission.” The main trade-offs are the transactions gained from assimilation and the costs of abandoning ancestral traits; these in part, depend on the time parents invest in teaching ancestral values. However, Giavazzi et al. (2019) does not examine the transmission of ancestral values that have evolved because of their higher economic evolutionary advantage, such as the cultural LTO traits in Galor and Özak (2016).^10^ Our work reinforces the relevance of ancestral traits that have a higher evolutionary advantage and are propagated through the mechanism of natural selection (Galor & Moav, 2002; Galor & Michalopoulos, 2012), adaptation, and intergenerational transmission.^11^

## Data

### Ancestry-adjusted crop yield measures

We obtain data on potential caloric crop yield and crop growth cycle available across the globe in grids with cells of size $5^{\prime }$ by $5^{\prime }$ and at the country level, measured using the Caloric Suitability Index (CSI) (Galor & Özak, 2015; Galor et al., 2016; Galor & Özak, 2016).^12^ The CSI estimates *potential* (not actual) caloric yield, are measured in calories per hectare per year, and are based on agro-climatic conditions under low levels of inputs and rain-fed agriculture that corresponds to cultivation methods in early stages of development. The measures distinguish between caloric suitability in the pre-1500 CE and post-1500 CE periods by basing the estimates in the pre-1500 CE period on the subset of crops in the Global Agro-Ecological Zoning/Food and Agricultural Organization data set which were available for cultivation in different regions of the world before 1500 CE, as documented by Crosby (1972) and Diamond (1997). In the post-1500 CE period, all regions could potentially cultivate all crops.

To estimate the causal impact of culturally embodied LTO traits, we correct for ancestry in crop yield measures in two steps. In the first step, we follow Galor and Özak (2016) and use the World Migration Matrix, 1500–2000, developed in Putterman and Weil (2010), to compute country-level ancestry-adjusted measures of the pre-1500 CE crop yield and crop growth cycle and their changes in the post-1500 CE period. The migration matrix in Putterman and Weil (2010) is available at the country level and details the 1500 CE origins of the present-day population for 165 countries. The entries in the matrix are estimates of the proportion of ancestors of the contemporary population who lived in each source country in 1500 CE.^13^

We follow the ancestry adjustment method in Galor and Özak (2016) and compute the ancestry-weighted agricultural measure for a country that takes into account 1500 CE ancestry by summing over the product of the ancestral composition of a given country, as predicted in the migration matrix and its corresponding agriculture measure.^14^ Next, we apply the ancestry IV, developed in Burchardi et al. (2019),^15^ which gives us plausibly exogenous variation in the distribution of country of origins in US counties. We create the final ancestry-adjusted measure for a given county by summing over the product of each country-of-origin composition in the county, as predicted by the ancestry IV in Burchardi et al. (2019), and the corresponding origin country’s agricultural measure that accounts for 1500 CE ancestry using the migration matrix of Putterman and Weil (2010).^16^

**Fig. 1:**
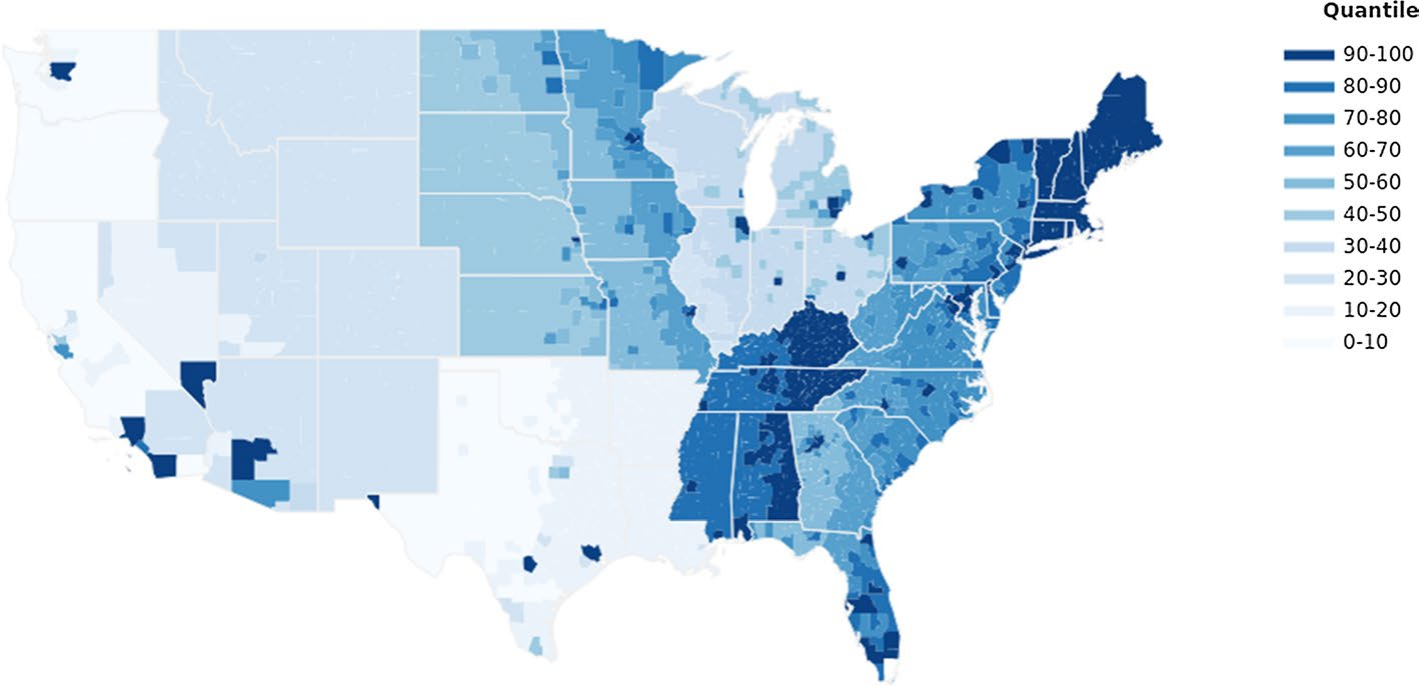
Ancestry-Adjusted Pre-1500 CE Potential Yields. Notes: This figure maps the distribution of ancestry-adjusted pre-1500 CE potential yield. The map’s color coding depicts the decile of pre-1500 CE potential yields across counties. Darker colors indicate a higher decile

**Fig. 2:**
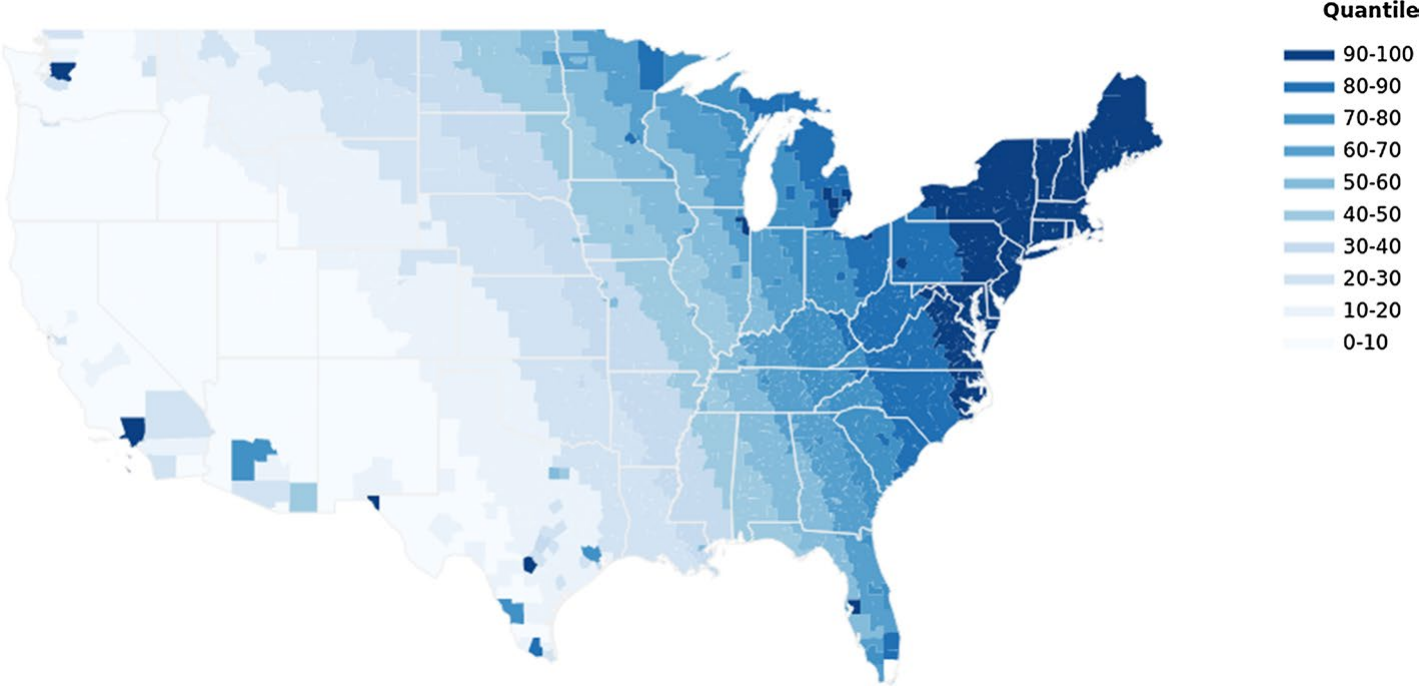
Ancestry-Adjusted Post-1500 CE Potential Yield Changes during the Columbian Exchange. Notes: This figure maps the distribution of ancestry-adjusted post-1500 CE potential yield change during the Columbian Exchange. The map’s color coding depicts the decile of post-1500 CE potential yield change across counties. Darker colors indicate a higher decile

Figure 1 illustrates the spatial variation in ancestry-adjusted pre-1500 CE crop yield, and Fig. 2 depicts the geographic variation in ancestry-adjusted post-1500 CE crop yield change across US counties. Higher crop yields and yield changes are marked with darker cells, and lower ones are marked with lighter cells.

### Outcome variables

Our primary outcomes of interest are county-level measures of disease severity that have substantial earnings and employment effects (Dobkin et al., 2018). No single measure can accurately capture COVID-19 severity. The total number of confirmed COVID-19 cases may not fully capture the severity of the pandemic, particularly during the early stages of the outbreak, because the number of reported cases depends on testing that was not widely available in the initial phase.^17^ Further, testing is likely to be targeted to individuals showing symptoms.

To mitigate concerns related to differences in testing, we measure county-level total COVID-19 cases and deaths per test conducted per 100,000. These measures effectively capture expected COVID-19 case or death prevalence in a county. Our monthly and cumulative county-level data on total COVID cases and deaths are from Johns Hopkins University Center for Systems Science and Engineering (Dong et al., 2020) for the period January 22, 2020 to November 30, 2020. The data on total monthly and cumulative county-level COVID-19 tests are obtained for the period March 15, 2020 to December 31, 2020 from the Centers for Disease Control and Prevention (CDC).^18^

The number of confirmed deaths from COVID-19 could underestimate the actual pandemic death toll even after accounting for testing. Reporting standards of measures, such as deaths due to COVID-19, may vary across jurisdictions. Further, the decision to report the underlying cause of death as COVID-19 infection involves discretion on the part of medical staff. Additionally, the pandemic could trigger deaths from non-COVID-19 causes by overwhelming hospitals, which may delay treatments for acute emergencies or chronic diseases. Therefore, we measure the impact on excess mortality.

Excess deaths for the year 2020 are defined as the total number of observed deaths from all causes in the year 2020 minus the expected deaths from all causes in the year 2020. One simple estimate of the expected deaths in 2020 is the product of the population denominator for the year under consideration and the mortality rate of the previous year. To mitigate any reverse causation bias from fatalities resulting from COVID-19, we do not use the population denominator for 2020 to estimate expected deaths for that year. We compute expected deaths in 2020 by multiplying the population denominator for 2019 with the mortality rates in 2018 using data from the CDC (see Appendix Sect. A.4 for details).^19^ Further, to explore the broader effects of LTO traits, we obtain facility-level data from the United States Department of Health and Human Services on the total number of staffed inpatient beds occupied and patients currently hospitalized in an adult inpatient bed who have laboratory-confirmed COVID-19. The sample includes facility-level data from hospital populations registered with the Centers for Medicare and Medicaid Services (CMS) and non-CMS hospitals that have reported hospital utilization use statistics from July 31 to November 30, 2020 (see Appendix A.5 for details).

Our hypothesis in this paper is that high culturally embodied LTO traits induce a higher willingness to socially distance, resulting in fewer COVID-19-related deaths and cases. To measure the effects on voluntary social distancing, we obtain data on mobility proxies that reflect individuals’ choice to socially distance—for example, mobility proxies measuring visits to workplaces might measure one’s occupational position rather than personal choice. Similarly, visits to groceries and pharmacies might be unavoidable. To capture the voluntary social distancing behavior of county residents in the US, we use GPS data from two independent sources that collect information on visits/visitors to recreational centers and non-essential trips: SafeGraph and Google Community Mobility Reports.^20^ Further, we expect that the changes in the COVID-19 cases will be observed for at least a few weeks after changes in mobility, as found in Cot et al. (2021). Therefore, we analyze changes in mobility up to a few weeks prior to the the end period of the analysis of COVID-19 cases and deaths.

To measure traffic patterns to a collection of points-of-interest (POIs), we obtain visit pattern data from SafeGraph, a data company that aggregates anonymized location data from about 45 million mobile devices across a wide range of carriers and numerous smartphone applications.^21^ One unique feature of GPS data from SafeGraph is that it identifies the visitors to each POI by their county of residence, allowing us to measure the behavioral response of county residents. Our analysis uses monthly county-level data from SafeGraph Patterns for the period February 1, 2020 to October 31, 2020 for POIs that include recreational places such as restaurants; clothing stores; hobby, toy, and game stores; fitness and recreational sports centers; and movie theaters (except drive-ins).

Further, our monthly data have information on the county location of each POI, POI category, the total number of visitors that have visited each POI, the total number of visits in each POI, and information about the home counties of all visitors to each POI category. Our first measure is the total visitors to a given recreational POI by county of residence. For each POI-county-month, the data are aggregated at the level of visitors’ county of residence. For a given county-month-POI, our second mobility proxy measures the total visits made by the residents of that county to places in that category during that month. For a given county-month-POI, we measure total visits by multiplying the total number of unique visitors from a given county to that POI during a given month with the average visits per visitor to the POI in a given county in a given month.

To provide robust evidence on the impact of LTO traits on the voluntary decision to socially distance, we complement our analysis on SafeGraph data with analysis using mobility indicators capturing recreational visits and time spent away from home from Google Community Mobility Reports. These reports are aggregate anonymized data from users who have opted to provide location history in their mobile devices, and they show how visits to (or time spent in) categorized places changed with respect to a baseline day.^22^ For each of the Google mobility proxies in each county, we take the monthly (four-week) average of the percentage change starting from 15th of every month to 14th of the next month for the period February 15, 2020 to November 14, 2020 in Google Community Mobility Reports.^23^

To capture non-essential trips, we use the “recreation” category in the report. Google Community Mobility Reports measure visits in counties but do not provide information on whether the visits are made by the county’s residents. Therefore, we use the “residential” category, which measures the duration of hours spent at residential locations and hence is more likely to capture the behavior of county residents.^24^ For ease of exposition, we compute an away-from-home proxy, which is a negative of the “Residential” category and which measures the percentage change in hours spent away from home relative to the pre-pandemic level.

The data on mobility indicators are based on smartphone owners who have requested travel direction or have opted to share their location history. One concern with these data is that individuals in our sample may have attributes that differ from those of the broader population. However, our data use a range of smartphone mobility applications to understand mobility changes in the US, where 81% of the population owns a smartphone (Pew Research Center, 2020).

### Additional variables

In order to address various identification concerns (as explained in the following Sect. 4), we collect data on a range of socioeconomic attributes, political orientation, and geographical characteristics (See Appendix-A.8 for details on variable construction and data sources.).

Since the analysis samples for COVID-19 severity measures and human mobility vary due to varying data availability, we show in Table 1 and in Appendix-Table B.1 that our analysis samples of different COVID-19 severity measures and mobility proxies are similar along most observables.

## Empirical strategy

We rely on Galor and Özak (2016) for identification of time preferences. The authors theoretically and empirically establish that societies in which the ancestral population was exposed to a higher potential crop yield (for a given crop growth cycle) had higher returns from agricultural investment, which induced selection, adaptation, and learning processes and increased the representation of LTO traits in the population over time. Further, societies that experienced additional increases in potential yield due to the expanded spectrum of suitable crops post-1500 CE had further gains in their degree of LTO traits. Galor and Özak (2016) also establish that crop yield measures do not affect a range of cultural characteristics such as individualism or collectivism, cooperation or competition, tolerance or rigidness, hierarchy, inequality of power, trust, and uncertainty avoidance. The impact of crop yield is not mediated by the above cultural covariates or by the potential consequences of past economic prosperity driven by higher agricultural productivity. More importantly, crop yield generated an evolutionary process in LTO traits without triggering a corresponding process in the evolution of risk aversion.

Galor and Özak (2016) use potential (instead of actual) crop yield measures under agro-climatic conditions in the early stages of development to avoid reverse causation bias in the analysis. The authors note that measures of the potential yield differ in terms of omitted variable bias concerns as well as in interpretations. The post-1500 CE potential yield measure is likely to be correlated with time-invariant omitted geographical attributes associated with contemporary economic outcomes that may determine the contemporary distribution of time preferences. The pre-1500 CE potential yield measure experienced by the ancestors mitigates, in several ways, the confounding effect of the potential current link between geographical characteristics and LTO traits that afflict the post-1500 CE potential yield measure. First, the pre-1500 CE measure captures the persistent historical effect of the ancestral homeland because it is based on crops grown before the Columbian Exchange. Second, the ancestral component of the measure reflects culturally embodied transmission rather than the direct effect of geography. However, the pre-1500 CE potential yield measure (given the pre-1500 CE crop growth cycle), which captures *levels* of culturally embodied LTO traits, can be potentially correlated with unobserved attributes of the ancestral homeland that may affect rewards from a having longer planning horizon and hence distribution of time preferences.

For an observed association between the potential yield measure experienced by ancestors and contemporary LTO traits of the descendants to be free of omitted variable bias concerns, an ideal experiment would require random assigning the potential yield measure to ancestors. This is achieved by the *change* in the crop yield measure associated with the Columbian Exchange because the post-1500 CE *change* in the crop yield and crop growth cycle are distributed randomly, conditional on pre-1500 levels (Galor & Özak, 2016). The post-1500 CE potential yield change measure captures an additional increase in LTO traits conditional on post-1500 CE change in the crop growth cycle, pre-1500 CE crop yield, and pre-1500 CE crop growth cycle. Following Galor and Özak (2016), we use pre-1500 CE potential yields and post-1500 yield changes experienced by ancestors of the current population as key, culturally-embodied LTO proxies.

In the US context, an exogenous random assignment of LTO proxies to ancestors is not sufficient to identify intergenerationally transmitted time preferences. As previously mentioned, most contemporary long-term residents of the US are not descendants of their territory’s inhabitants circa 1500 CE but are people whose ancestors were migrants from Europe, Africa, and other countries (Putterman & Weil, 2010). This generates a mismatch between the crop yield in the US counties and the crop yield to which the ancestral population of US residents was exposed in 1500 CE. Therefore, we use Putterman and Weil (2010) to correct for ancestry in crop yield and growth cycle measures for US county populations.

Even after identifying where the ancestors of current US residents lived in 1500 CE, the ancestry-adjusted measures have potential endogeneity concerns. It is possible that historical migrations might have occurred between origins and destinations with omitted climatic, cultural, institutional, or other characteristics, and these characteristics might, in turn, determine contemporary economic outcomes correlated with pandemic severity. This could lead to a spurious correlation between culturally embodied LTO traits and pandemic outcomes. There could be additional concerns—for example, the ancestral homeland could be selectively reported based on past and present socio-economic conditions (Perez & Hirschman, 2009). Therefore, we apply the ancestry IV, developed in Burchardi et al. (2019), to crop yield measures corrected for ancestry in 1500 CE to isolate the variation in the distribution of ancestry that is plausibly independent of unobserved factors that could potentially influence COVID-19 transmissions.

We estimate the following empirical specification via ordinary least squares (OLS) regression to measure the effect of LTO proxies:

1 $$\begin{aligned} y_{i} =\alpha + \beta _{0} \times yield_{i} + \beta _{1} \times \Delta yield_{i}+ \beta _{2} \times cycle_{i} + \beta _{3} \times \Delta cycle_{i} + \eta _{s} + \sigma X_{i}+ \epsilon _{i}, \end{aligned}$$

where $y_{i}$ is the outcome of interest for county *i*, $yield_{i}$ and $cycle_{i}$ are the ancestry-adjusted potential crop yield and crop growth cycle at pre-1500 CE level for county *i*, $\Delta yield_{i}$ is the ancestry-adjusted yield change in the post-1500 CE period, and $\Delta cycle_{i}$ is the ancestry-adjusted crop growth cycle change in the post-1500 CE period during the Columbian Exchange for county *i*.

The LTO proxy measured by the ancestry-adjusted post-1500 CE yield change is unlikely to have omitted variable bias concerns because it is independently distributed among the ancestral population conditional on pre-1500 CE levels (Galor & Özak, 2016). However, the LTO proxy measured by the ancestry-adjusted pre-1500 potential yield may be correlated with other unobserved attributes of ancestry since it is not randomly assigned to ancestors. Therefore, $X_{i}$ includes covariates that account for potential confounders that may bias the estimated effect of the pre-1500 CE potential yield measure.

The pre-1500 potential yield may be associated with unobservables of the ancestral homeland that influence industrial composition, affecting hours available for leisure and hence the ability to make non-essential trips, which impacts coronavirus transmission. Therefore we include the contribution to the percent change in real GDP by private goods–providing industries (2019), government enterprises (2019), and the percentage change in annual average employment for a given year (2019) as controls that account for the industrial makeup of each county.

The pre-1500 CE potential yields experienced by ancestors may also be associated with unobserved ancestral social norms that affect socio-economic^25^ and political factors^26^ correlated with COVID-19 prevalence, such as political partisanship, age or gender distribution, housing choice, choice of family structure, population density, underlying health conditions, and preference for public transport. Therefore, $X_{i}$ adds the proportion of votes for the Democrats in counties during the 2016 US presidential election to control for political preference. $X_{i}$ also includes socio-economic controls^27^ measured at pre-crisis levels comprising of county-level dummy variables for the urban status of each county including large central or large fringe metro counties and medium metro and small metro counties; mean income; proportion of males; population density; proportion of Black or African American, Indian American and Native Alaskan, White alone, proportion of population of Hispanic or Latino origin; share of population using public transport; proportion of family and non-family households living in two or more unit structures and proportion of family and non-family households with three or more members; a wide range of age groups (under 19, 19–34, 35–64, and 65 and above); share of population with an education level higher than or equal to the higher secondary level; proportion working from home; health-care coverage comprising the proportion of the population with two or more health insurance policies in the age groups under 19, 19–34, 35–64, and 65 and above; the proportion of those above 65 years without any insurance; distance to an airport with direct international flights to high-severity countries; Gini index and share of population below the poverty line; Social Capital Index; the percentage of adults with obesity and the percentage of adult smokers; 30-day risk-adjusted mortality rate; heart disease death rate, and percentage diagnosed with diabetes among adults above 20 years of age. $X_i$ includes county-level COVID-19 tests per 100,000 for dependent variables that do not take into account COVID-19 testing.

The ancestry-adjusted pre-1500 CE potential yield proxy may be associated with ancestral genetic factors that could influence COVID transmission through its responsiveness to geographic factors. Thus, we include geographical controls such as elevation, terrain roughness, temperature, and precipitation, which have been shown to affect COVID-19 transmission.^28^ Apart from observables, we account for unobserved state-varying norms and COVID-19 policies with a set of state fixed effects, $\eta _{s}$.

The county-level coefficient estimates of the impact of LTO traits may be biased if there are cross-county population movements, leading to a potential violation of SUTVA (Rubin, 1980). The pandemic severity of neighboring counties may also drive county-level effects of LTO traits on COVID-19 prevalence measures. Further, dependent error structures across counties could result in erroneous inference. In our county-level analysis, $X_{i}$ includes controls for mobility and COVID-19 severity and testing in neighboring counties to address the identification concern associated with cross-county interaction. We address the inference concern by clustering our standard errors, $\epsilon _{i}$, at the CZ level using various bootstrap methods of inference to account for correlated shocks across contiguous counties.^29^ To test for robustness across various methods of inference, we report standard errors that account for arbitrary spatial correlation by using the acreg package developed in Colella et al. (2019) that follows Conley (1999).^30^ However, to further mitigate identification and inference concerns emerging from cross-county population interactions, we replicate the whole analysis at the CZ level.

One potential concern related to controls in our setting is that pre-crisis socio-economic and political preference characteristics can be affected by LTO proxies and hence can be viewed, in accordance with Angrist and Pischke (2008), as "bad controls" that may increase bias in the estimates. However, recent advances in the bad control literature^31^ do not view this condition as a criterion to distinguish good controls from bad controls. Whether controls are good controls or bad controls in a regression depend on the target quantity of the analysis. Our hypothesis is that LTO traits affect people’s willingness to social distance, resulting in lower COVID-19 severity. If LTO traits affect mobility behavior directly by affecting people’s inherent willingness to comply with social distancing norms and indirectly by changing the pre-crisis distribution of demographic and economic characteristics correlated with COVID norm compliance, then controlling for socio-economic covariates allows us to measure the impact of LTO traits through its effect on the inherent willingness to invest in COVID-safe behavior, which is precisely our target quantity of interest. Similar arguments for identification can be made in relation to including controls that measure neighboring county behavior and COVID-19 prevalence. We are interested in estimating the impact of LTO traits of county residents on their voluntary compliance with COVID-19 norms and resultant disease prevalence. The estimate of this impact can be confounded by mobility behavior and caseloads of neighboring counties, which could be affected by LTO traits. Therefore, controlling for neighboring county outcomes isolates the mediating effects from the neighboring county population.

Our first coefficient of interest is $\beta _{0}$, which measures the causal effect of the ancestry-adjusted pre-1500 CE potential crop yield, a proxy for the level of culturally embodied LTO traits. Our second coefficient of interest is $\beta _{1}$, which captures the causal effect of changes in the ancestry-adjusted potential crop yields during the Columbian Exchange, where increases in the ancestry-adjusted potential crop yields during the Columbian Exchange proxy for additional increases in LTO traits, beyond the initial levels of LTO generated by pre-1500 CE crops. We expect $\beta _{0}<0$ and $\beta _{1}<0$ for measures of COVID-19 severity; that is, we expect that intergenerationally transmitted LTO traits reduce COVID-19 severity. We then explore whether ancestral LTO traits reduce COVID-19 prevalence by increasing compliance with social distancing norms. We use mobility proxies for non-essential trips and duration of time away from home as outcome variables and expect $\beta _{0}<0$ and $\beta _{1}<0$, indicating that a culturally embodied, future-oriented outlook causes voluntary compliance with stay-at-home norms.

The panel structure of the data on COVID-19 deaths, cases, and mobility proxies allows us to estimate the trajectories of causal effects after the onset of the pandemic in an event study framework. We use the following equation as our baseline specification to analyze the differential paths of high LTO versus low LTO traits:

2 $$\begin{aligned} Y_{it} = \sum _{k\ne -1}\beta _{k} High LTO_{i}*1(t=k) + \mu _i + \gamma _t + \delta X_{it}+ \epsilon _{it}, \end{aligned}$$

where $Y_{it}$ are county-level measures of COVID-19 severity and mobility proxies from county *i* at time *t*. We report results with two alternative definitions of high LTO counties. In our first specification, $High LTO_{i}$ is an indicator that takes a value of 1 if the ancestry-adjusted pre-1500 CE potential yield is above the sample median. In an alternative specification, $High LTO_{i}$ takes a value of 1 if the ancestry-adjusted post-1500 CE potential yield change due to the Columbian Exchange is above the median of the sample.

In the case of mobility proxies, $1(t=k)$ is a dummy variable for every month, with February (2020), which is the month before the declaration of a national emergency in response to the coronavirus, as the omitted month.^32^ However, for the measures of COVID-19 cases and deaths that *account* for tests conducted in each county, we could not use monthly data from January to March 15, 2020 because testing data from that period are zero for 95% of the observations. Moreover, the data on COVID-19 cases during the initial phase of the pandemic are likely to have high measurement errors. Therefore, for COVID-19 severity measures, our $1(t=k)$ is a dummy variable for every month from April to November, with April (2020) as the omitted month.

$\mu _i$ is the county fixed effect controlling for omitted time-invariant confounders varying at the county level, and the $\gamma _t$ are time fixed effects. $X_{it}$ is a vector of time-varying controls such as temperature, precipitation, COVID-19 cases, and mobility proxies in neighboring counties. $\epsilon _{it}$ is the error term. We report *p*-values based on inference conducted using the wild cluster bootstrap method (Roodman et al., 2019) because it is appropriate for panel data cases where error terms or data are not i.i.d. In addition, the wild bootstrap method is a more general method and is shown to be more accurate than the popular “pairs bootstrap” method (MacKinnon, 2006). However, we find that inference based on both methods yields similar results in our setting, as shown in Sect. 7.

Our coefficient of interest $\beta _{t}$ captures the trajectory of the causal effects of high LTO traits. We further augment our baseline specification with state–time trends to control for differential time trends across high and low LTO counties. In Sect. 7, we also address the issue of potential differences in pre-trends using the methodology of Rambachan and Roth (2019) that provides valid inference under violations of the parallel trends assumptions.

## Impact of LTO traits on COVID-19 severity

In this section, we examine the role of time preferences in cross-county heterogeneity in COVID-19 severity in the US in 2020.

### COVID-19 case-prevalence and death-prevalence

We estimate the empirical specification in Eq. 1 via OLS to measure the causal effects of ancestral LTO traits on county-level total confirmed COVID-19 cases and deaths per test administered per 100,000 during the period from January 22, 2020 to November 30, 2020. We analyze data up until the onset of COVID-19 vaccination rollouts because the vaccination drive will confound the identification of the association between time preferences and COVID-19 infection rates in the analysis.

Column (1) of Panel A in Table 2 establishes the relationship between the ancestry-adjusted post-1500 CE potential yield change during the Columbian Exchange and COVID-19 cases per test conducted per 100,000, accounting for time-invariant unobservables at the state level. We find that an increase of one standard deviation in the ancestry-adjusted potential yield change decreases COVID-19 case prevalence by 1.2 standard deviations, which is statistically significant at the 1% level across various methods of inference that account for clustering at the CZ level and arbitrary spatial autocorrelation. Column (1) of Panel B shows that an increase of one standard deviation in the ancestry-adjusted potential yield change decreases deaths from COVID-19 by 1.06 standard deviations, which is statistically significant at the 1% level, robust to different inference methods.

Column (2) accounts for confounding geographical differences, such as terrain roughness, elevation, precipitation, and temperature. The effect of the ancestry-adjusted post-1500 CE potential yield change due to the Columbian Exchange remains negative, stable, and statistically significant at the 1% level for the COVID-19 case prevalence measure in Panel A and the COVID-19 death prevalence measure in Panel B. For both the COVID-19 case prevalence and COVID-19 death prevalence, the effect of the ancestry-adjusted potential yield change in the post-1500 CE period is larger than any of the geographical attributes that we use as controls. The estimated coefficient on the pre-1500 CE potential crop yield is negative, large, and imprecise.

In columns (3) and (4), we include controls measured at the pre-crisis level. Column (3) considers the confounding effect of mobility behaviors and the COVID-19 infection prevalence of neighboring counties. Reassuringly, the coefficient on the ancestry-adjusted post-1500 CE potential yield change remains stable and statistically significant at the 1% level for both COVID-19 case and COVID-19 death prevalence in panels A and B, respectively. The estimated coefficients on geographical attributes remain smaller than the effect of the ancestry-adjusted potential yield change.

Additionally, the COVID-19 case prevalence in neighboring counties increases COVID-19 case prevalence, but the magnitude is relatively small and marginally statistically significant. Similarly, the estimated coefficient on COVID-19 case prevalence in neighboring counties has no statistically significant effect on the COVID-19 death prevalence measure. The results indicate that spillover effects are marginal and unlikely to bias our county-level estimates. Column (4) accounts for a range of socio-economic, demographic covariates, underlying health conditions, county-level industry composition, and changes in employment. The effect of the ancestry-adjusted post-1500 CE potential yield change remains stable at $-$1.04 standard deviations for COVID-19 case prevalence and $-$0.98 standard deviations for COVID-19 death prevalence.

**Fig. 3:**
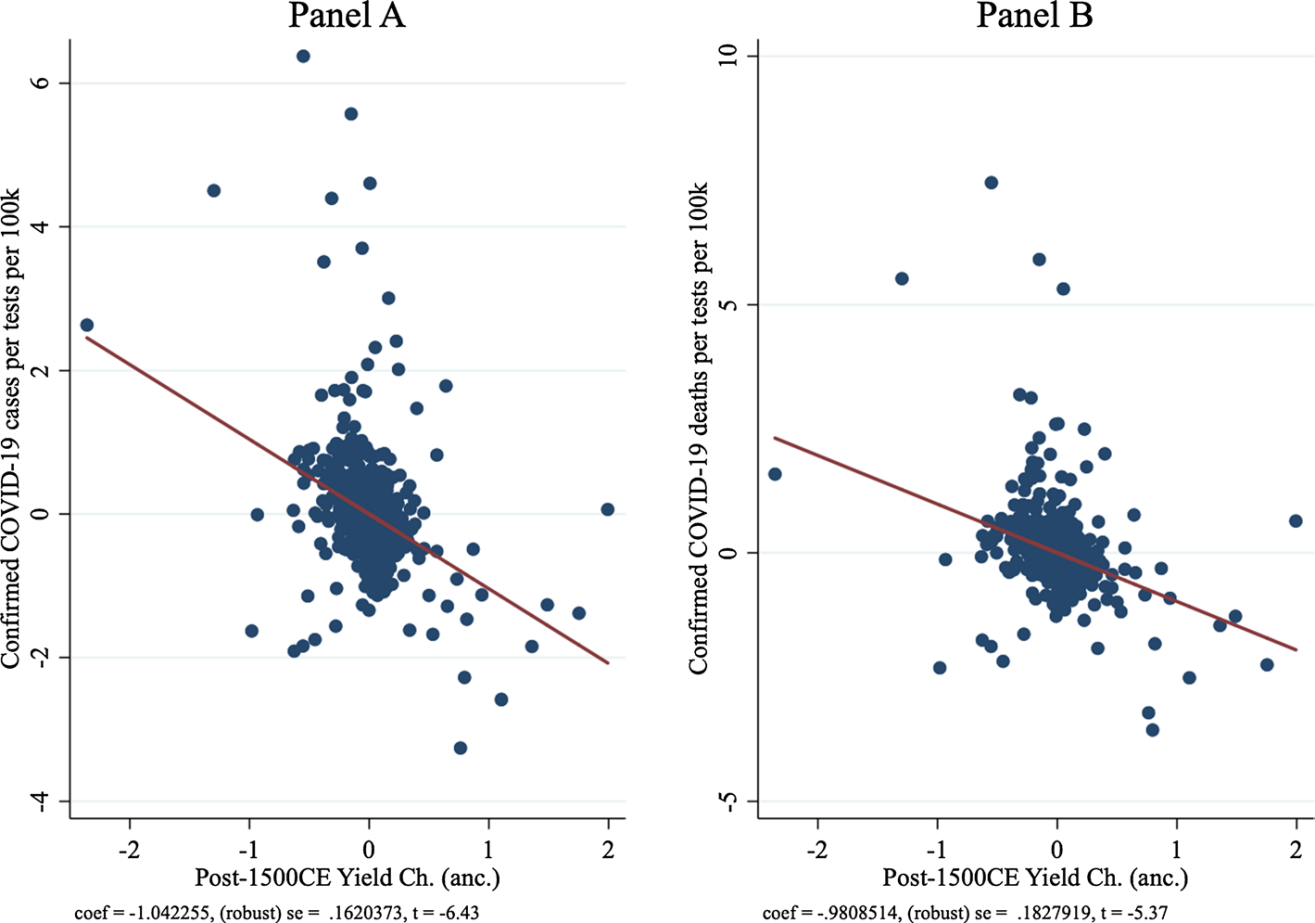
Impact of Long-term Orientation Traits. Notes: The figure illustrates the negative effect of the ancestry-adjusted potential yield change in post-1500 CE period on COVID-19 cases per test per 100,000 (panel **A**) and COVID-19 deaths per test per 100,000 (panel **B**). The depicted relationships account for the full set of controls presented in column(4) of Table 2

Panel A of Fig. 3 depicts the partial correlation plot for column (4) for COVID-19 case prevalence, and Panel B of Fig. 3 depicts the partial correlation plot for COVID-19 death prevalence. Overall, we find that the coefficient estimate on the ancestry-adjusted post-1500 CE potential yield change is robust and very stable at approximately—1 standard deviation. Consistent with our hypothesis, the coefficient estimate on the ancestry-adjusted pre-1500 CE crop yield is negative but very large and imprecise. The stable and robust coefficient estimate on the ancestry-adjusted post-1500 CE potential yield change reinforces the assumption in Galor and Özak (2016) that the change in the crop yield during the Columbian Exchange is likely to be free of omitted variable bias.

Our second empirical strategy exploits the panel data structure to measure the dynamic effect of culturally embodied LTO traits on COVID-19 severity. The estimation approach uses within-county variation, which allows us to control for omitted county-level time-invariant unobservables and time effects. Columns (1)–(4) of Appendix Table B.3 report the effects of high LTO traits, where high LTO traits are defined as counties with ancestry-adjusted pre-1500 CE potential crop yield above the sample median. Columns (5)–(8) of Appendix Table B.3 report the effects of high LTO traits, where high LTO traits are defined as counties with ancestry-adjusted post-1500 CE potential yield change above the sample median. All specifications control for monthly average precipitation, temperature, mobility, and COVID-19 cases in neighboring counties.

Column (1) of Appendix Table B.3 shows that high LTO traits have persistent negative effects on COVID-19 case prevalence: $-$0.34 standard deviations in May, $-$0.31 standard deviations in June, $-$0.30 standard deviations in July, $-$0.30 standard deviations in August, $-$0.31 standard deviations in September, $-$0.31 standard deviations in October, and $-$0.30 standard deviations in November, all statistically significant at the 1% level. When controlling for differential state trends that possibly capture state COVID-19 policies varying over time in column (2), the effects increase slightly. Columns (3) and (4) show similar persistent negative statistically and economically significant effects on COVID-19 death prevalence.

In columns (5) to (8), where we measure high LTO traits with the ancestry-adjusted post-1500CE yield change, we observe persistent statistically significant negative effects of COVID-19 death prevalence, without and with controlling for state–time trends. The effect on COVID-19 case prevalence is negative, which becomes statistically significant toward the later phase of the pandemic when state trends are added. Including state trends only marginally changes the magnitude of the treatment effects.

Both empirical strategies provide consistent evidence that ancestral LTO traits substantially reduce measures of COVID-19 cases and deaths. However, there could be errors in the measurement of deaths attributed to COVID-19. To fully capture the effects on mortality, we next explore the impact on excess deaths.

### Age-specific excess deaths (2020)

We analyze excess deaths for older age groups. This is because of selection concerns associated with samples for younger age groups owing to the CDC’s data suppression policy whereby total deaths below 10 are not reported. Table 3 shows that for all age-specific excess death outcomes across all specifications, the effect of the ancestry-adjusted post-1500 CE potential yield change is negative, stable, and statistically significant at the 1% level, robust to different inference methods. In column (4), which accounts for the full set of controls,^33^ the magnitude of the effect of the ancestry-adjusted post-1500 CE yield change is $-$0.81 standard deviations for excess deaths of people age 45 years and over (2 times less than the sample average (mean $=326$). The effects are similar for excess deaths of people aged 55 years and over and 65 years and over, which are $-$0.86 standard deviations (2.6 times less than the average (mean $=189$)) and -$-$0.86 standard deviations (2.7 times less than the sample average (mean $=132$)) respectively. The coefficient estimate on the ancestry-adjusted pre-1500 CE potential yield is negative but imprecise. The pattern found here is consistent with the above findings on COVID-19 case and death prevalence.

**Fig. 4:**
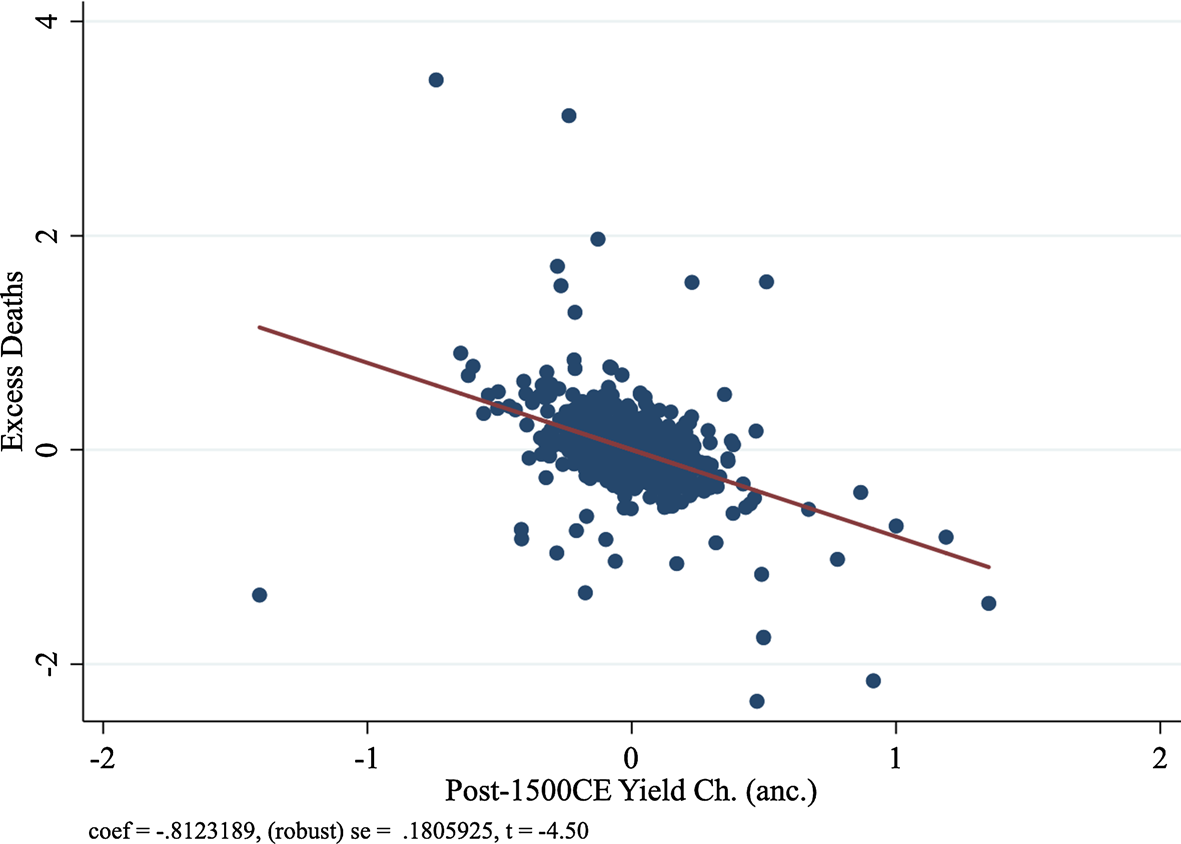
Impact of Long-term Orientation Traits on Excess Deaths (45 and above). Notes: The figure illustrates the negative effect of the ancestry-adjusted potential yield change in post-1500 CE period on excess deaths in people aged 45 and older. The sample includes only counties that have reported deaths in every five-year age group from age 45 and older. The depicted relationships account for the full set of controls presented in column (4) of Table 3

**Fig. 5:**
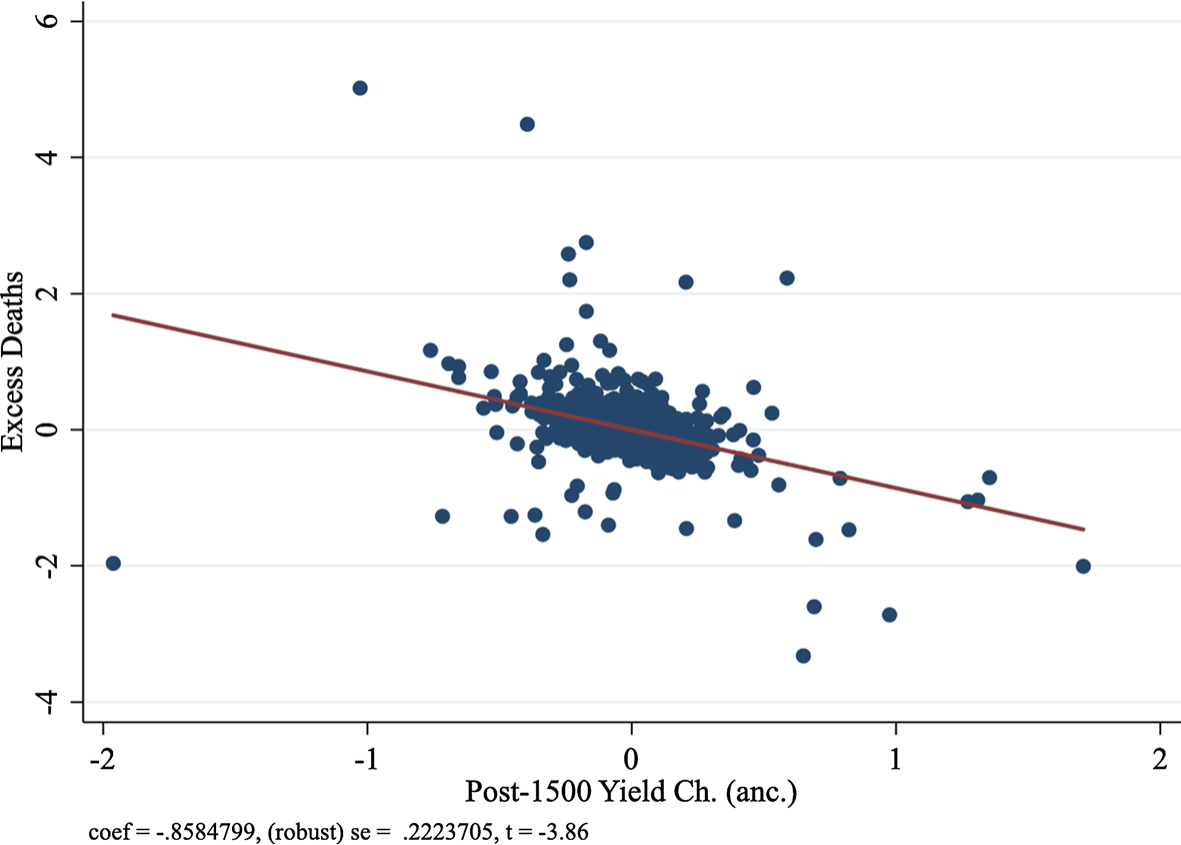
Impact of Long-term Orientation Traits on Excess Deaths (55 and above). Notes: The figure illustrates the negative effect of the ancestry-adjusted potential yield change in post-1500 CE period on excess deaths in people aged 55 and older. The sample includes only counties that have reported deaths in every five-year age group from age 55 and older. The depicted relationships account for the full set of controls presented in column (4) of Table 3

**Fig. 6:**
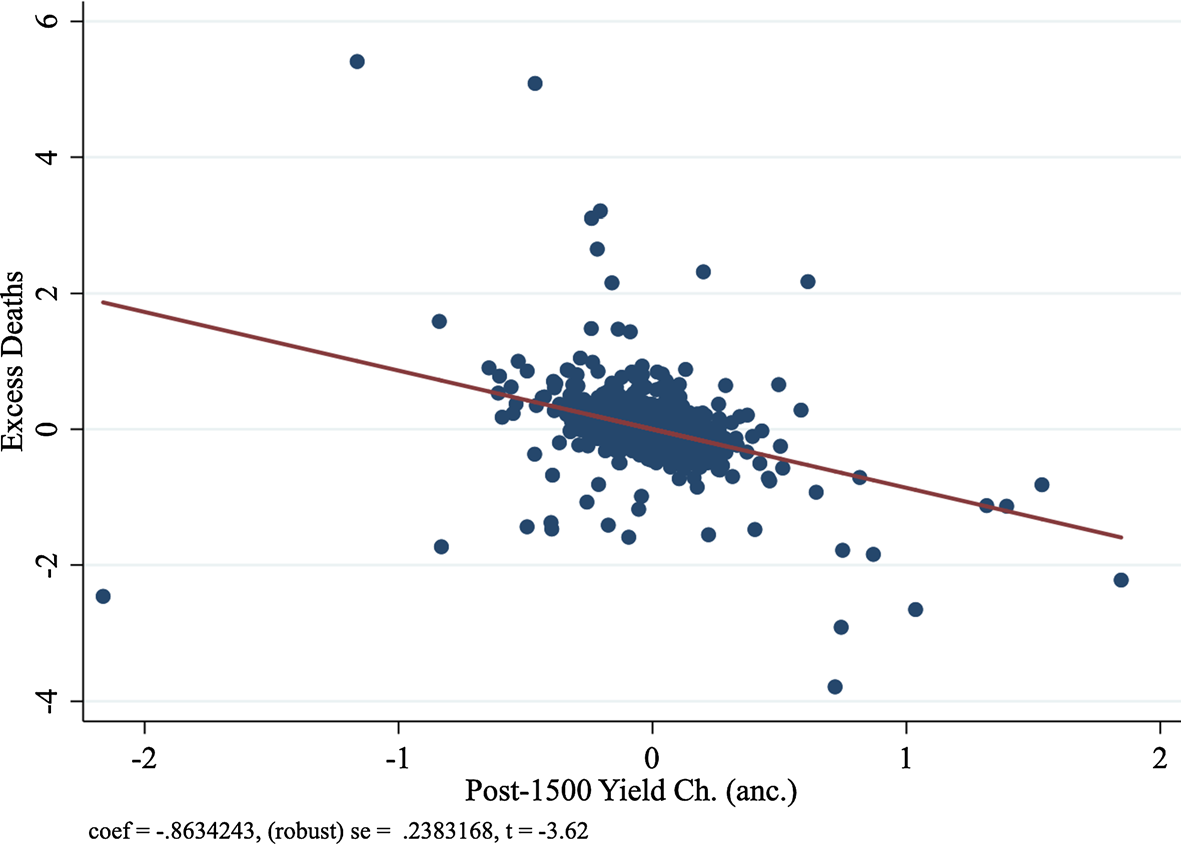
Impact of Long-term Orientation Traits on Excess Deaths (65 and above). Notes: The figure illustrates the negative effect of the ancestry-adjusted potential yield change in post-1500 CE period on excess deaths in people aged 65 and older. The sample includes only counties that have reported deaths in every five-year age group from age 65 and older. The depicted relationships account for the full set of controls presented in column (4) of Table 3

Figures 4, 5 and 6 illustrate the partial correlation plot for excess mortality in the age group 45 and above, age group 55 and above, and age group 65 and above respectively.

### Additional outcomes

Panel A of Appendix Table B.4 reports the effects of ancestral LTO traits on the county-level prevalence of hospitalization from COVID-19, which is measured as the total adults hospitalized with COVID-19 per test per 100,000. Consistent with the above results for mortality outcomes, we find that the measure of LTO traits based on the ancestry-adjusted post-1500 CE yield change has a negative effect on the prevalence of hospitalization from COVID-19 across specifications and is statistically significant at the 1% level, which is robust to various methods of clustering standard errors at the CZ level and accounting for arbitrary spatial correlation. The coefficient estimate on the ancestry-adjusted post-1500 CE potential yield change is $-$1.14 standard deviations when only state fixed effects are accounted for. The estimate remains stable at $-$1.04 standard deviations after adding geographic controls, is almost unchanged at $-$0.99 standard deviations when mobility and COVID-19 prevalence in neighboring counties have been added, and is $-$0.96 standard deviations after adding the controls for hospital capacity,^34^ in addition to our full set of socio-economic controls. The effect of the LTO traits as measured by the ancestry-adjusted pre-1500 CE crop yield remains negative across specifications, but the magnitude is large and imprecise.

Panel B of Appendix Table B.4 reports the effect of LTO on the county-level average of the seven-day sum of inpatient beds used per hospital. The effect of LTO traits measured by the ancestry-adjusted pre-1500 CE potential yield is statistically significant but too large when pre-existing hospital capacity, underlying health conditions, socio-demographic, and economic and political attributes are not accounted for. The estimate reduces substantially after accounting for socio-economic, political, and health confounders, indicating that the ancestry-adjusted pre-1500 CE potential yield has omitted variable bias concerns. The coefficient estimate on the ancestry post-1500 CE yield change is not statistically significant, although the sign of the effect is negative, which is consistent with our hypothesis.^35^

### Testing for omitted variable bias for COVID-19 cases and deaths and excess mortality

The Columbian Exchange resulted in an increase in potential yield if and only if the potential yield of a newly introduced crop is higher than the potential yield of the dominating crop prior to the exchange. Therefore, conditional on pre-1500 CE levels, the potential distribution of superior crops during the natural experiment is independent of omitted attributes of the region. The ancestry-adjusted measure of the potential yield change further adjusts for ancestry by using the variations in historical leave-out push–pull shocks, which are independent of plausible confounders that make a given US county attractive for both migrations and economic transactions, and could potentially be correlated with COVID-19 transmissions.

We use statistics on the selection of observables and unobservables (Oster, 2019) to establish that omitted variable bias is unlikely to explain the estimated effect of LTO traits on the measures of pandemic severity in the pre-vaccination phase. Using the method in Oster (2019), which is based on the assumption that the relationship between treatment and unobservables can be recovered from that of the treatment and observed covariates, we show in Appendix Table B.5, that the bias-adjusted estimated effect of the ancestry-adjusted post-1500 CE potential yield change is strictly negative with a magnitude larger than the OLS estimate.

### Sorting versus inter-generational transmission of culture

Galor and Özak (2016) establish that the potential yield triggers a gradual propagation of higher LTO traits through the forces of natural selection and cultural evolution. A priori, a positive relationship between potential yield measures and LTO traits could potentially be driven by the sorting of high LTO individuals into high-yield locations. Therefore, the relationship between crop yield measures and pandemic outcomes could potentially be interpreted as being driven by a sorting effect. As argued in Galor and Özak (2016), if a positive relationship between yields and LTO traits is an outcome of sorting, then one would observe a stronger relationship between unadjusted potential yield measures and LTO traits compared with the relationship between ancestry-adjusted crop yield measures and LTO in a sample where post-1500 CE migration is high. Galor and Özak (2016) find that the effect of the ancestry-adjusted potential yield on LTO is stronger than the effect of the unadjusted measures in the whole world sample where migration is prevalent, reinforcing the hypothesis that the impact of the potential yield on contemporary time preferences is culturally transmitted.^36^

In our context, we find that the intergenerationally transmitted cultural LTO traits predict variations in COVID-19 severity measures. The potential preindustrial crop yield measures of the territory, which do not account for the ancestry of current residents, do not have any effect on COVID-19 case or death prevalence, as reported in Appendix Table B.6; on excess deaths, as presented in Appendix Tables B.7–B.9; or on hospitalization rates or inpatient bed utilization rates, as presented in Appendix Tables B.10 and B.11 respectively. We report the horse race between ancestry-adjusted and unadjusted measures of crop yield and yield change conditional on the crop growth cycle and its change after accounting for a full set of controls in column (4) of Appendix Tables B.6–B.11. Reassuringly, across all outcomes (except for inpatient bed utilization rate), we find that it is the ancestry-adjusted post-1500 CE crop yield change due to the Columbian Exchange that have statistically and economically significant negative effects that are robust to various methods of computing standard errors. For inpatient bed utilization rates, we find that it is the ancestry-adjusted pre-1500 CE potential yield that is economically and statistically significant.

## Effects of LTO traits on compliance with social distancing

Mobility measures reflecting the level of human contact are highly correlated with transmission rates (Nouvellet et al., 2021). Therefore, our results on the negative effect of culturally embodied LTO traits on COVID-19 severity could be driven by their effects on mobility. After an initial decline, mobility trends in the US indicate a subsequent increase in mobility across all age groups by mid-2020 (Monod et al., 2021). In this section, we examine whether visits to recreational centers or non-essential trips, potentially indicative of pandemic fatigue behavior across US counties, is driven by a lower prevalence of future-oriented mindsets that could potentially drive individuals’ choice to conform with social distancing.

We first estimate the empirical specification in Eq. 1 via OLS to measure the effect of LTO traits on non-essential trips. Table 4 shows the effect of the ancestry-adjusted pre-1500 CE potential yield and the ancestry-adjusted post-1500 CE potential yield change on total visitors by county in hobby centers and gaming stores in Panel A, restaurants and other eating places in Panel-B, and movie theaters (except drive-ins) in Panel C. We find that a one standard deviation increase in the ancestry-adjusted pre-1500 CE potential crop yield, which is a historical proxy for LTO traits, decreases total visitors across all the above POIs. The coefficient estimate on the ancestry-adjusted pre-1500 CE potential crop yield is implausibly large in magnitude and the degree of statistical significance varies across inference methods and outcome variables. In contrast, the effect of the ancestry-adjusted post-1500 CE potential crop yield change decreases the total number of visitors across all POIs, and the magnitude is economically significant and robust to various inference methods.

Table 5 presents similar patterns for visitors to clothing centers and fitness centers. In line with the findings on COVID-19 severity, in the cross-sectional empirical strategy the effects of the ancestry-adjusted pre-1500 CE yield are too large. However, the effects of the ancestry-adjusted post-1500 CE yield change are –1 standard deviation for most mobility proxies, statistically significant at the 1% level and robust to various inference methods that account for spatial correlation and noise from estimated regressors.

Total visitors from counties measures only the extensive margin of human mobility. We next examine the impact on total visits made by county residents, which is the intensive margin of human mobility proxies. Appendix Tables B.12 and B.13 report the effects of the ancestry-adjusted pre-1500 CE potential yield and the ancestry-adjusted post-1500 CE potential yield change on total visits. The results for the total visits are similar to our above findings on total visitors.

We next report the effects on Google mobility proxies based on Eq. 1. Appendix Table B.14 shows that culturally embodied LTO traits decrease visits to recreation centers. We find that a one standard deviation increase in the ancestry-adjusted pre-1500 CE potential yield results in a decrease in the visits to recreation centers that is statistically significant for months toward the later phase of the pandemic ($-$4.63 standard deviations in September and $-$3.78 standard deviations in October), indicating higher pandemic fatigue behavior in counties with a lower representation of cultural future-oriented mindset. A one standard deviation increase in the ancestry-adjusted post-1500 CE potential yield change decreases the visits to recreation centers in the initial phase of the pandemic to $-$0.18 standard deviations, indicating that populations exhibiting higher culturally embodied LTO traits are more willing to adhere to social distancing measures. However, the effect of the ancestry-adjusted post-1500 CE is noisy toward the later phase of the pandemic. Consistent with the results on visits to recreation centers, the effect of the ancestry-adjusted pre-1500 CE potential yield on time spent away from home is negative and is statistically significant at the 5% level toward the later phase of the pandemic ($-$4.85 standard deviation in October). The effect of the ancestry-adjusted post-1500 CE potential yield change on time spent away from home is noisy.

In our second empirical strategy, where we estimate Eq. 2, we measure high LTO in two ways: (1) above-median ancestry-adjusted pre-1500 CE potential yield and (2) above-median ancestry-adjusted post-1500 CE potential yield change. We establish that the effect of high ancestral LTO traits on the total county visitors or visits to restaurants, movie theaters, clothing stores, and fitness and recreation centers are negative, persistent, and statistically significant at the 1% level. This is in line with our hypothesis and is consistent with our first estimation approach, which follows specification in Eq. 1. For most proxies, the magnitude of the effect in the later months of the pandemic is higher than during the immediate months post declaration of the pandemic, suggesting that pandemic fatigue behavior is less prevalent among populations with higher cultural future-oriented outlooks.

Columns (1)–(4) of Appendix Tables B.15–B.19 report the effects of a higher prevalence of ancestral LTO traits proxied by the ancestry-adjusted pre-1500 CE potential yield above the sample median. Columns (5)–(8) of the above tables report the effects of higher prevalence of culturally embodied LTO traits proxied by the ancestry-adjusted post-1500 CE potential yield change due to the Columbian Exchange above the sample median. Below, we report the magnitude of treatment effects based on specifications accounting for time-varying geographic attributes, COVID-19 incidence and mobility proxies of the neighboring counties, and state trends, presented in columns (2), (4), (6), and (8) of Appendix Tables B.15–B.19.

Appendix Table B.15 shows that the effect of the ancestry-adjusted high pre-1500 CE yield on total visitors (total visits) to restaurants ranges from $-$0.15 ($-$0.18) standard deviations in March to $-$0.32 ($-$0.37) standard deviations in October, statistically significant at the 1% level. The effect of the ancestry-adjusted high post-1500 yield change on total visitors (visits) to restaurants is statistically significant at the 1% level and ranges from from $-$0.07 ( $-$0.10) standard deviations in March and $-$0.29 ($-$0.33) standard deviations in October.

In Appendix Table B.16, we find economically and statistically significant effects for the measures of visitors and visits to movie theaters. Column (2) (column (4)) shows that the effects of the ancestry-adjusted high pre-1500 CE yield measure on total visitors (total visits) ranges from $-$0.58 ($-$0.54) standard deviations in March to $-$2.11 (–2) standard deviations in October. We find statistically significant effects of ancestry-adjusted post-1500 CE yield change on total visitors (total visits) to movie theaters, which ranges from $-$0.36 ($-$0.32) standard deviation in March and $-$1.61 ($-$1.52 ) standard deviation in October, as depicted in column (6) (column (8)) in Appendix Table B.16.

Column (2) (column (4)) of Appendix Table B.17 shows that the effect of the ancestry-adjusted high pre-1500 CE yield on total county visitors (total visits) to clothing stores is $-$0.18 ($-$0.18) standard deviations in March and $-$0.14 ($-$0.20) standard deviations in October, statistically significant at the 1% level. Columns (6) and (8) shows that the effect of the ancestry-adjusted high post-1500 CE yield change on total county visitors (total visits) to clothing stores is $-$0.08 ($-$0.08) standard deviations in March, statistically significant at the 10% level. For total visitors, the effect persists until September. For total visits to clothing stores, the magnitude of the effect is $-$0.15 standard deviations in October, statistically significant at the 5% level.

Accounting for the full set of time-varying controls and state trends presented in columns (2), (4), (6) and (8) of Appendix Table B.18, we find the following effects on total visitors (visits) to fitness and recreation centers. The ancestry-adjusted high pre-1500 CE yield has treatment effects of $-$0.25 ($-$0.31) standard deviations in March and $-$0.67 ($-$0.71) standard deviations in October, while the treatment effects of the ancestry-adjusted high post-1500 CE yield change are $-$0.14 ($-$0.17) standard deviations in March and $-$0.49 ($-$0.50) standard deviations in October, all statistically significant at the 1% level. Appendix Table B.19 shows that the effect of cultural LTO traits on visitors (visits) to hobby centers are not robustly persistent over time.

When analyzing Google mobility indicators using Eq. 2, we find that a higher prevalence of culturally embodied measures for LTO traits causes a shorter duration of time spent away from home and fewer visits to recreational locations. Column (1) of Appendix Table B.20 shows high ancestral future-oriented mindset as measured by the ancestry-adjusted high pre-1500 CE yield decreases the duration of time spent away from home, where the magnitude of the difference between high LTO versus low LTO counties is $-$0.53 standard deviations in March; the effect persisting with $-$0.87 standard deviations in October, statistically significant at the 1% level. Column (3) shows a similar pattern for the effect of LTO traits, measured by the ancestry-adjusted high post-1500 CE yield change, on the duration of time away from home, where the magnitude of the difference between the high versus low LTO counties are $-$0.56 standard deviations in March and $-$0.69 standard deviations in October, statistically significant at the 1% level. Column (2) shows that the ancestry-adjusted high pre-1500 CE yield decreases visits to recreation locations with the effect ranging from $-$2.18 standard deviations in March to $-$9.02 standard deviations in October, statistically significant at the 1% level. Column (4) presents consistent results for the effect of LTO traits measured by the ancestry-adjusted high post-1500 CE yield change on the visits to recreational locations. The magnitude of the effect ranges from $-$1.43 standard deviations in March to $-$3.15 standard deviations in October, statistically significant at the 1% level.

Both measures of ancestral LTO traits show that intergenerationally transmitted future-oriented outlooks decrease the total number of visitors and visits to each POI, that is robust to the inclusion of state–time trends. In addition, the magnitudes of treatment effects in the event study specification are economically significant and not implausibly large. The treatment effects for the ancestry-adjusted pre-1500 CE measure in the dynamic setting are similar to the ancestry-adjusted post-1500 yield change measure for most mobility proxies. Overall, the treatment effects on mobility and COVID-19 severity are in line with our hypothesis that the populations with a higher representation of LTO traits are more willing and continue to adhere to Non Pharmaceutical Interventions (i.e., social distancing) and therefore experience lower COVID-19 severity.

## Sensitivity analysis for pre-trends

The key identifying assumption in event study designs is the assumption of parallel trends. In other words, pre-treatment differences in trends inform the counterfactual differences in trends after the event. One concern with the identifying assumption is that it is possible for human mobility behavior to have differential pre-pandemic trends across high and low LTO counties. In this section, we conduct sensitivity analyses in which we report confidence sets under a set of possible violations of parallel trends assumption using the methods of Rambachan and Roth (2019).

We perform sensitivity analysis on mobility proxies from SafeGraph^37^ by augmenting our baseline specification, namely Eq. 2, with pre-treatment mobility proxies. Appendix Table B.21 shows that for all recreational locations in the SafeGraph data, there were more visitors from counties with higher LTO traits, where high LTO traits are defined by the ancestry-adjusted high pre-1500 CE potential yield, *before* the declaration of a national emergency. However, the magnitude of the differences is very marginal. In contrast to the ancestry-adjusted pre-1500 CE potential yield variable, the treatment variable ancestry-adjusted high post-1500 CE yield change has no statistically significant treatment effects on the pre-period monthly mobility proxies for almost all the POIs.^38^ Further, Appendix Table B.21 shows that the results are robust to various bootstrap methods.

We next analyze confidence sets of treatment effects for both linear and non-linear violations of parallel trends for high LTO trait measures based on the ancestry-adjusted pre-1500CE potential yield. Guided by the pre-period data, we impose the additional restriction that the violation of parallel trends in mobility proxies is positive in the post period. We find in Appendix Tables B.22–B.26 that high LTO trait counties exhibit a higher compliance to voluntary social distancing by most measures of mobility proxies that is robust to various linear and non-linear violations of parallel trends.

## Replicating the analysis at the commuting zone level

Cross-county population movements could confound the identification of county-level coefficient estimates. To address this concern, we replicate our entire analysis at the CZ level, which are geographic units that define local spatial labor markets. We establish, in cross-sectional analysis at the CZ level in Appendix Table B.27, that the ancestry-adjusted potential yield change in the post-1500 CE period has negative effects on COVID-19 incidence, mortality, and hospitalization, which are significant at the 1% level. The coefficient estimates are $-$1.71 standard deviations for COVID-19 cases per test per 100,000 and $-$1.24 standard deviations for COVID-19 deaths per test per 100,000. The effect ranges from $-$1.18 to $-$1.46 standard deviations for excess mortality outcomes.

The effect of the ancestry-adjusted pre-1500 CE potential yield is $-$9.80 standard deviations for COVID-19 cases per test per 100,000 and $-$5.91 standard deviations for seven-day total inpatient beds used per hospital, statistically significant at the 10% and the 5% level respectively. The ancestry-adjusted pre-1500 CE potential yield measure also has negative effects on excess mortality outcomes, which are imprecise.

In Appendix Tables B.28 and B.29, we show that the effect of the ancestry-adjusted post-1500 CE yield change in the cross-sectional analysis is around –1 standard deviation across most POIs and mobility measures in the SafeGraph dataset. Similar to the estimated effects on COVID-19 severity measures analyzed at the CZ level, the effect of the ancestry-adjusted pre-1500CE potential yield is negative but imprecise.

Appendix Table B.30 presents results using Google mobility data. We find that a one standard deviation increase in the ancestry-adjusted post-1500 CE potential yield change causes an effect of $-$0.42 standard deviations on visits to recreational locations in April. The effects in later months are negative but imprecise. The treatment effect of the ancestry-adjusted post-1500 CE potential yield change on the duration of hours spent away from home is negative and persistent with $-$0.284 standard deviation effect in September that is statistically significant at the 10% level. The effects of the pre-1500 CE potential yield on visits to recreation centers are imprecise. The treatment effect of the pre-1500 CE potential yield on the duration of hours spent away from home is negative, large, and marginally statistically significant.

In Appendix Table B.31, we report the dynamic effects on COVID-19 case and death prevalence at the CZ level. We find similar patterns to those found in the county-level analysis. The treatment effect of the ancestry-adjusted high pre-1500 CE potential yield spans from $-$0.49 standard deviation in May to $-$0.39 in November for COVID-19 cases per test per 100,000 and $-$0.34 standard deviation in May to $-$0.40 standard deviation in November for COVID-19 deaths per test per 100,000, statistically significant at the 1% level. The coefficient estimate on the ancestry-adjusted high post-1500 CE potential yield change is economically and statistically significant for the COVID-19 death prevalence measure, with the magnitude of the effect spanning from $-$0.32 standard deviations in March to $-$0.29 standard deviation in November. The impact of the ancestry-adjusted high post-1500 CE yield change on COVID-19 cases is negative and imprecise.

The event study analysis at the CZ level of SafeGraph Patterns data, presented in Appendix Tables B.32–B.36, show that high LTO trait counties as measured by the ancestry-adjusted high pre-1500 CE potential yield and the ancestry-adjusted high post-1500 CE potential yield change have persistent statistically significant negative effects on total visitors and total visits to most recreational locations, consistent with the county-level analysis. The dynamic treatment effects in Google mobility data present a similar pattern for recreational visits as presented in Appendix Table B.37. The effects on the duration of hours away from home are negative, but the magnitudes are small and not statistically significant for later months. Overall, analyses at the CZ level yield results consistent with the county-level findings.

## Conclusion

Long-term orientation traits with bio-geographical origins causally explain the heterogeneity in COVID-19 severity in the US in 2020, when the US was the epicenter of the pandemic. In particular, we show that counties with a higher representation of future-oriented mindsets among the ancestral population exhibit less resistance to protracted social distancing, have fewer COVID-19 cases, and have better mortality outcomes.

Identifying the causal effects of time preferences on pandemic severity is an empirically challenging task because unobserved contemporary factors may co-determine variations in time preferences and COVID-19 severity. To identify the effect of time preferences, we leverage the exogenous variation in bio-geographical origins of culturally embodied long-term orientation traits established in Galor and Özak (2016). Since our study context is the US, where a large fraction of the current population is descended from other countries, we first use the World Migration Matrix, 1500–2000 (Putterman & Weil, 2010) to adjust for ancestry in the proxies of ancestral time preference traits using the method in Galor and Özak (2016). There still remains possible selection concerns associated with selective historical migration or the selective reporting of ancestry. We resolve the above endogeneity concerns by applying the set of instrumental variables for contemporary ancestry composition in the US developed in Burchardi et al. (2019) to ancestry adjustments made using Galor and Özak (2016).

To measure the impact of culturally embodied future-oriented outlooks on voluntary compliance with social distancing and resultant disease severity, we use GPS data from independent data sources on a range of non-essential trips during the pre-vaccination phase and analyze various measures of COVID-19 severity. We study both cross-sectional and dynamic impacts of intergenerationally transmitted LTO traits at the county-level and show that our findings are robust to different methods of inference and empirical specifications. Additionally, we replicate our entire county-level analysis at the commuting zone level to address potential concerns arising from cross-county population interactions. We find that our treatment effect estimates at the commuting zone level are consistent with county-level estimates.

Long-term orientation traits that have been transmitted over time—through evolutionary processes of natural selection, adaptation, learning, and other modes of intergenerational transmission—are shown in this paper to be at the heart of socially desirable responses to the global crisis.

**Table 1:** Descriptive Statistics

	(1)	(2)	(3)	(4)	(5)	(6)
Analysis samples	COVID-19 cases/deaths	Ex. deaths ($\ge$45)	Ex. deaths ($\ge$55))	Ex. deaths ($\ge$65)	COVID-19 hosp	Inpatient bed use
Total counties	2034	895	1480	1751	1693	1702
Mean income (USD)	68427.35	72050.1	69023.02	68477.17	69321.06	69332.82
	*(13396.31)*	*(15431.75)*	*(14315.65)*	(13775.5)	*(13454.9)*	*(13424.43)*
Tot. pop	115835.3	240716.6	155666.8	133689.9	135163.1	134597.9
	*(378530.7)*	*(545654.7)*	*(437167.7)*	*(405,174)*	*(412073.5)*	*(411055.8)*
HH with $\ge 2$ unit structure (share)	0.5543	0.7178	0.6191	0.5865	0.5965	0.5960
	(0.3299)	(0.3582)	(0.3404)	(0.3332)	(0.3321)	(0.3321)
HH with $\ge 3$ members (share)	0.5305	0.5630	0.5458	0.5386	0.5349	0.5344
	(0.0747)	(0.0660)	(0.0687)	(0.0708)	(0.0742)	(0.0745)
Female (share)	0.4994	0.5061	0.5024	0.5008	0.5004	0.5002
	(0.0214)	(0.0135)	(0.0183)	(0.0199)	(0.0195)	(0.0199)
Black pop. (share)	0.0895	0.1084	0.1054	0.1002	0.0867	0.0860
	(0.1441)	(0.1358)	(0.1493)	(0.1501)	(0.1407)	(0.1398)
Indian American and Alaska Native pop. (share)	0.0180	0.0141	0.0155	0.0164	0.0181	0.0182
	(0.0620)	(0.0527)	(0.0586)	(0.0577)	(0.0590)	(0.0589)
White pop. (share)	0.8327	0.8027	0.8154	0.8224	0.8320	0.8322
	(0.1582)	(0.1505)	(0.1605)	(0.1604)	(0.1556)	(0.1547)
Hispanic or Latino* (share)	0.0991	0.1072	0.0963	0.0954	0.1027	0.1033
	(0.1435)	(0.1366)	(0.1317)	(0.1350)	(0.1410)	(0.1410)
Public transport (share)	0.0075	0.0117	0.0087	0.0081	0.0083	0.0084
	(0.0184)	(0.0250)	(0.0202)	(0.0190)	(0.0199)	(0.0205)
Working from home (share)	0.0495	0.0441	0.0444	0.0458	0.0489	0.0492
	(0.0270)	(0.0198)	(0.0213)	(0.0228)	(0.0254)	(0.0257)
H.S graduate or higher (share)	0.8687	0.8719	0.8662	0.8669	0.8726	0.8727
	(0.0618)	(0.0562)	(0.0585)	(0.0594)	(0.0602)	(0.0601)
Under 19 yrs (share)	0.2490	0.2519	0.2488	0.2483	0.2504	0.2501
	(0.0350)	(0.0301)	(0.0321)	(0.0333)	(0.0338)	(0.0340)
19–34 yrs (share)	0.1804	0.1921	0.1866	0.1833	0.1832	0.1831
	(0.0351)	(0.0328)	(0.0347)	(0.0345)	(0.0350)	(0.0352)
35–64 yrs (share)	0.3830	0.3836	0.3839	0.3842	0.3814	0.3815
	(0.0271)	(0.0255)	(0.0265)	(0.0262)	(0.0265)	(0.0265)
Above 65 yrs (share)	0.1876	0.1724	0.1807	0.1842	0.1851	0.1853
	(0.0446)	(0.0415)	(0.0425)	(0.0432)	(0.0440)	(0.0442)
$\ge$ 2 health insurance (under 19) (share)	0.0622	0.0615	0.0622	0.0627	0.0629	0.0631
	(0.0273)	(0.0213)	(0.0237)	(0.0252)	(0.0263)	(0.0263)
$\ge$ 2 health insurance (19–34) (share)	0.0610	0.0600	0.0607	0.0609	0.0614	0.0615
	(0.0273)	(0.0188)	(0.0232)	(0.0244)	(0.0256)	(0.0256)
$\ge$ 2 health insurance (35–64) (share)	0.1012	0.1017	0.1036	0.1031	0.1001	0.1002
	(0.0313)	(0.0290)	(0.0295)	(0.0303)	(0.0302)	(0.0302)
$\ge$ 2 health insurance ($\ge$65) (share)	0.6990	0.6940	0.6966	0.6979	0.6993	0.6993
	(0.0656)	(0.0589)	(0.0605)	(0.0624)	(0.0648)	(0.0647)
% Obesity	35.2317	34.3261	35.0730	35.1750	34.9946	34.9451
	(4.5286)	(4.7698)	(4.6602)	(4.6068)	(4.6040)	(4.6230)
% Diabetes	10.5284	10.8384	10.9482	10.8183	10.4291	10.4175
	(3.4546)	(3.0635)	(3.3929)	(3.4386)	(3.3658)	(3.3612)
% Smokers	21.2884	20.6579	21.4328	21.4614	20.9770	20.9453
	(4.0603)	(4.3417)	(4.2618)	(4.1624)	(3.9782)	(3.9816)
Heart disease rate per 100k	184.0556	184.5404	187.7250	186.4204	182.2980	182.1661
	(43.1865)	(44.9196)	(44.0567)	(43.5564)	(42.9085)	(42.8318)
30-day risk adjusted mortality rate	0.4625	0.3451	0.4676	0.4809	0.4631	0.4654
	(1.0786)	(0.8439)	(0.9415)	(0.9816)	(1.0404)	(1.0396)
Perc. Ch. GDP by services industries	2.2152	1.8888	2.1226	2.1893	2.0530	2.0951
	(2.5441)	(2.0046)	(2.1990)	(2.3945)	(2.3829)	(2.4475)
Perc. Ch. GDP by Pvt. goods industries	0.6251	0.3016	0.3294	0.4042	0.6653	0.6610
	(5.1807)	(2.4987)	(3.2627)	(3.9444)	(4.9322)	(4.9155)
Perc. Ch. GDP by Govt. enterprises	0.0930	0.1344	0.0993	0.0923	0.0917	0.0914
	(0.5791)	(0.5375)	(0.5309)	(0.5529)	(0.5126)	(0.5124)
Perc. Ch. in annual avg. empl.	0.5798	0.9022	0.7301	0.6059	0.5379	0.5441
	(3.1428)	(1.9079)	(2.4280)	(2.7823)	(3.0026)	(2.9965)
Gini Index	0.3813	0.4138	0.3994	0.3906	0.3864	0.3863
	(0.0848)	(0.0785)	(0.0796)	(0.0815)	(0.0865)	(0.0862)
Pop. below poverty line (share)	0.1430	0.1358	0.1436	0.1435	0.1398	0.1399
	(0.0637)	(0.0609)	(0.0637)	(0.0641)	(0.0616)	(0.0614)
Precipitation (mm/month)	86.1769	96.1929	94.1806	91.4396	85.0073	84.8353
	(33.2898)	(30.8682)	(30.7765)	(31.7098)	(33.6475)	(33.6180)
Temperature (celsius)	13.4444	14.5415	14.1735	13.8426	13.2626	13.2498
	(4.9585)	(4.6114)	(4.7251)	(4.8306)	(4.9792)	(4.9678)
Elevation (metres)	476.7445	414.3564	415.9409	436.3273	497.1921	505.0485
	(523.1734)	(490.8341)	(497.4843)	(510.0766)	(533.8980)	(540.5600)

The table presents summary statistics for our sample analyzed in Table 2 in column (1); Table 3 in columns (2), (3), and (4); and Appendix Table B.4 in columns (5) and (6). Standard deviations are reported in parentheses.

**Table 2:** Impact of Long-Term Orientation on COVID-19 Severity

January 22, 2020 to November 30, 2020				
Panel A COVID-19 case per test per 100k	(1)	(2)	(3)	(4)
Pre-1500CE yield (Anc.)	$-$6.612	$-$9.307	$-$8.845	$-$7.135
	[5.049]	[5.372]*	[4.802]*	[4.686]
	(4.504)	(4.714)**	(4.189)**	(3.915)*
	$\langle p=0.185 \rangle$	$\langle p=0.0941 \rangle$	$\langle p=0.061 \rangle$	$\langle p=0.137 \rangle$
Post-1500CE yield Ch. (Anc.)	$-$1.213	$-$1.130	$-$1.066	$-$1.042
	[0.169]***	[0.173]***	[0.178]***	[0.192]***
	(0.158)***	(0.159)***	(0.153)***	(0.150)***
	$\langle p=0.000 \rangle$	$\langle p=0.000 \rangle$	$\langle p=0.000 \rangle$	$\langle p=0.000 \rangle$
Pre-1500CE crop cycle (Anc.)	5.444	7.780	7.210	5.607
	[5.204]	[5.372]	[4.806]	[4.738]
Post-1500CE cycle Ch. (Anc.)	2.493	2.707	2.710	2.551
	[0.448]***	[0.505]***	[0.459]***	[0.454]***
Roughness of terrain		$-$0.0472	$-$0.100	$-$0.0778
		[0.0513]	[0.0557]*	[0.0513]
Avg. precipitation		$-$0.0364	$-$0.0251	$-$0.00346
		[0.0484]	[0.0465]	[0.0469]
Avg. temperature		0.366***	0.308***	0.197**
		[0.114]	[0.0860]	[0.0839]
Avg. elevation		0.120*	0.158**	0.0944
		[0.0679]	[0.0723]	[0.0666]
Avg. neighbor COVID-19 cases per tests/100k			0.134	0.112
			[0.0745]*	[0.0747]
Observations	2,034	2,034	2,034	2,034
R-squared	0.767	0.777	0.799	0.825

Panel B: COVID-19 deaths per test per 100k	(1)	(2)	(3)	(4)
Pre-1500CE yield (Anc.)	$-$6.468	$-$8.657	$-$8.006	$-$6.731
	[4.958]	[5.090]*	[5.023]	[5.249]
	(4.348)	(4.346)**	(4.235)*	(4.382)
	$\langle p=0.202 \rangle$	$\langle p=0.120\rangle$	$\langle p=0.138\rangle$	$\langle p=0.249 \rangle$
Post-1500CE yield Ch. (Anc.)	$-$1.056	$-$0.981	$-$0.936	$-$0.981
	[0.177]***	[0.183]***	[0.200]***	[0.221]***
	(0.149)***	(0.154)***	(0.164)***	(0.169)***
	$\langle p=0.000 \rangle$	$\langle p=0.000 \rangle$	$\langle p=0.000 \rangle$	$\langle p=0.000 \rangle$
Pre-1500CE crop cycle (Anc.)	6.146	8.030	7.359	6.243
	[5.302]	[5.306]	[5.269]	[5.466]
Post-1500CE cycle Ch. (Anc.)	1.883	2.070	2.025	1.926
	[0.489]***	[0.546]***	[0.535]***	[0.512]***
Roughness of terrain		$-$0.0774*	$-$0.112**	$-$0.109**
		[0.0425]	[0.0512]	[0.0494]
Avg. precipitation		$-$0.0063	0.0072	0.0229
		[0.0375]	[0.0381]	[0.0450]
Avg. temperature		0.316	0.266	0.167
		[0.102]***	[0.0814]***	[0.0821]**
Avg. elevation		0.150	0.171	0.139
		[0.0679]**	[0.0744]**	[0.0790]*
Avg. neighbor COVID-19 cases per tests/100k			0.0868	0.0653
			[0.0731]	[0.0683]
Observations	2,034	2,034	2,034	2,034
R-squared	0.787	0.794	0.803	0.816
Geographic	N	Y	Y	Y
Neighbor mob & COVID-19 prev	N	N	Y	Y
Socio-economic, pol., health	N	N	N	Y
State fixed effects	Y	Y	Y	Y

Standard errors are denoted as follows: [bootstrap standard errors account for clustering at the commuting zone level using the bootstrap command with the cluster option in STATA; that is, in this method, the sample drawn during each replication is a bootstrap sample of commuting zones] and (standard errors are adjusted for arbitrary spatial clustering using the acreg package written by Colella et al. (2019)). Stars $$^{*** (**)[*]}$$ indicate significance at the 0.01(0.05)[0.1] level. $$\langle$$*p*-values are generated by the wild cluster bootstrap method using the boottest command written by Roodman et al. (2019), clustered at the commuting zone level$$\rangle$$. The table reports the effects of the ancestry-adjusted pre-1500 CE potential crop yield and the ancestry-adjusted post-1500 CE crop yield change due to the Columbian Exchange on county-level COVID-19 case prevalence in panel A and county-level COVID-19 death prevalence in panel B, based on OLS estimates of Eq. 1. COVID-19 case prevalence in a county is measured as the total COVID-19 confirmed cases per test per 100,000. The COVID-19 death prevalence in a county is measured as total COVID-19 deaths per test per 100,000. All columns have state fixed effects, and all variables are normalized by subtracting their mean and dividing by their standard deviation. Therefore, all coefficients are comparable and estimate the effect of a one standard deviation increase in the independent variable. Column (1) includes only ancestry-adjusted pre-1500 CE crop yield, crop growth cycle, and their changes. Column (2) adds geographic controls such as mean roughness of terrain, mean precipitation, average temperature, and average elevation. Column (3) adds controls for county-level COVID-19 prevalence in neighboring counties, which is measured as the confirmed COVID-19 cases per test per 100,000 averaged over all neighboring counties. Further, for each county, we generate controls for mobility in neighboring counties by applying principal component analysis (PCA) on five mobility proxies that measure the average number of total visitors from neighboring counties to the following five POIs: hobby centers, restaurants, clothing stores, fitness centers, and movie theaters (except drive-ins). The first four components (PCA) that explain 99% of variation are added in column (3). Column (4) adds dummy variables for the urban status of each county (composed of large central metro, large fringe metro, medium metro, or small metro county, as defined by the National Center for Health Statistics); mean income; proportion of males; population density; proportion of population from a Black or African American, Native American, White, and Hispanic or Latino background; proportion of population working from home; proportion of population using public transport; proportion of family and non-family households living in two or more unit structures; proportion of family and non-family households with three or more members; proportion of population in age groups younger than 19, 19–34, and 35–64 (with 65 and older as the omitted group); proportion of population with an education level higher than or equal to higher secondary level; healthcare coverage measured as the proportion of the population with two or more health insurance policies in the age groups younger than 19, 19–34, 35–64, and 65 and older; and proportion of population older than age 65 without any health insurance. Column (4) further adds distance to an airport with direct international flights to high-severity countries measured following the method in Desmet and Wacziarg (2021); Gini index and proportion of population below the poverty line; Social Capital Index; percentage of adult population with obesity; percentage of population who smoke as adults; 30-day risk-adjusted mortality rate; heart disease death rate; percentage of population diagnosed with diabetes among adults older than 20 years of age; contribution to the percentage change in real GDP by private-services-providing industries, private-goods-providing industries, and government enterprises, and government enterprises; percentage change in the annual average employment for a given year; and the proportion of votes for Democrats in counties in the 2016 US presidential election as a proxy for pre-crisis political orientation. Predicted ancestral compositions in each county using the instruments from Burchardi et al. (2019) and the post-1500 World Migration Matrix of Putterman and Weil (2010) were used to adjust crop yield measures

**Table 3:** Impact of Long-Term Orientation on Excess Deaths

January 1, 2020 to December 31, 2020				
Panel A dependent variable: excess deaths 45 and above	(1)	(2)	(3)	(4)
Pre-1500 CE crop yield (Anc.)	2.027	0.0404	0.272	$-$0.451
	[4.225]	[4.321]	[4.563]	[4.416]
	(3.709)	(3.791)	(3.940)	(4.056)
	$\langle p=0.633 \rangle$	$\langle p=0.9890 \rangle$	$\langle p=0.950 \rangle$	$\langle p=0.931 \rangle$
Post-1500 CE yield Ch. (Anc.)	$-$0.811	$-$0.772	$-$0.762	$-$0.812
	[0.189]***	[0.187]***	[0.190]***	[0.216]***
	(0.163)***	(0.166)***	(0.162)***	(0.182)***
	$\langle p=0.001 \rangle$	$\langle p=0.001 \rangle$	$\langle p=0.001 \rangle$	$\langle p=0.001 \rangle$
Pre-1500 CE cycle (Anc.)	$-$2.107	$-$0.162	$-$0.347	0.772
	[4.522]	[4.568]	[4.843]	[4.669]
Post-1500 cycle Ch. (Anc.)	1.535	1.537	1.490	1.281
	[0.596]**	[0.607]**	[0.615]**	[0.613]**
Observations	895	895	895	895
R-squared	0.874	0.879	0.883	0.903

Panel B dependent variable: excess deaths 55 and above	(1)	(2)	(3)	(4)
Pre-1500 CE crop yield (Anc.)	1.299	$-$0.549	$-$0.201	$-$0.886
	[4.600]	[4.737]	[4.986]	[4.996]
	(4.211)	(4.222)	(4.343)	(4.573)
	$\langle p=0.805 \rangle$	$\langle p=0.917 \rangle$	$\langle p=0.972 \rangle$	$\langle p=0.881 \rangle$
Post-1500 CE yield Ch. (Anc.)	$-$0.877	$-$0.831	$-$0.813	$-$0.858
	[0.229]***	[0.229]***	[0.233]***	[0.256]***
	(0.205)***	(0.207)***	(0.204)***	(0.223)***
	$\langle p=0.0020 \rangle$	$\langle p=0.003 \rangle$	$\langle p=0.001 \rangle$	$\langle p=0.002 \rangle$
Pre-1500 CE cycle (Anc.)	$-$1.294	0.414	0.114	1.194
	[4.951]	[5.026]	[5.290]	[5.264]
Post-1500 CE cycle Ch. (Anc.)	1.576**	1.647**	1.589**	1.397**
	[0.639]**	[0.683]**	[0.684]**	[0.659]**
Observations	1,480	1,480	1,480	1,480
R-squared	0.861	0.865	0.868	0.883

Panel C dependent variable: excess deaths 65 and above	(1)	(2)	(3)	(4)
Pre-1500 CE crop yield (Anc.)	1.519	$-$0.330	0.195	$-$0.223
	[4.758]	[4.914]	[5.113]	[4.965]
	(4.402)	(4.419)	(4.501)	(4.694)
	$\langle p=0.778 \rangle$	$\langle p=0.956 \rangle$	$\langle p=0.975\rangle$	$\langle p=0.970 \rangle$
Post-1500 CE yield Ch. (Anc.)	$-$0.880	$-$0.831	$-$0.806	$-$0.863
	[0.245]***	[0.247]***	[0.249]***	[0.279]***
	(0.223)***	(0.224)***	(0.219)***	(0.240)***
	$\langle p=0.005 \rangle$	$\langle p=0.006 \rangle$	$\langle p=0.006 \rangle$	$\langle p=0.005 \rangle$
Pre-1500 CE cycle (Anc.)	$-$1.353	0.307	$-$0.172	0.653
	[5.069]	[5.129]	[5.365]	[5.111]
Post-1500 CE cycle Ch. (Anc.)	1.486	1.589	1.523	1.337
	[0.660]**	[0.720]**	[0.721]**	[0.690]**
Observations	1,751	1,751	1,751	1,751
R-squared	0.850	0.854	0.857	0.872
Geographic	N	Y	Y	Y
Neighbor mob& COVID-19 prev	N	N	Y	Y
Socio-economic, pol., health	N	N	N	Y
State fixed effects	Y	Y	Y	Y

Standard errors are denoted as follows: [bootstrap standard errors account for clustering at the commuting zone level using the bootstrap command with the cluster option in STATA; that is, in this method, the sample drawn during each replication is a bootstrap sample of commuting zones] and (standard errors are adjusted for arbitrary spatial clustering using the acreg package written by Colella et al. (2019)). Stars $$^{*** (**)[*]}$$ indicate significance at the 0.01(0.05)[0.1] level. $$\langle$$*p*-values are generated by the wild cluster bootstrap method using the boottest command written by Roodman et al. (2019), clustered at the commuting zone level$$\rangle$$. The table reports the effects of the ancestry-adjusted proxies of LTO traits based on OLS estimates of Eq. 1. Excess deaths for the age group "x" years and older is calculated as the total deaths in the age group in 2020 minus the product of the 2018 death rate in the age group and the 2019 population in the age group. All columns have state fixed effects, and all variables are normalized by subtracting their mean and dividing by their standard deviation. Therefore, all coefficients are comparable and estimate the effect of a one standard deviation increase in the independent variable. Column (1) includes only pre-1500 CE crop yield, crop growth cycle, and their changes. Column (2) adds geographic controls comprising the mean roughness of the terrain, precipitation, average temperature, and average elevation. Column (3) adds controls for county-level COVID-19 prevalence in neighboring counties, measured as the confirmed COVID-19 cases per test per 100,000 averaged over all neighboring counties. Further, for each county, we generate controls for mobility in neighboring counties by applying PCA on five mobility proxies that measure the average total number of visitors from neighboring counties to five POIs: hobby centers, restaurants, clothing stores, fitness centers, and movie theaters. The first four components (PCA) that explain 99% of the variation are added in column (3). Column (4) adds county-level COVID-19 tests per 100,000; dummy variables for the urban status of each county (composed of large central metro, large fringe metro, medium metro, or small metro county, as defined by the National Center for Health Statistics); mean income; proportion of males; population density; proportion of population from a Black or African American, Native American, White, and Hispanic or Latino background; proportion of population working from home; proportion of population using public transport; proportion of family and non-family households living in two or more unit structures; proportion of family and non-family households with three or more members; proportion of population in age groups younger than 19, 19–34, and 35–64 (with 65 and older as the omitted group); proportion of population with an education level higher than or equal to higher secondary level; healthcare coverage measured as the proportion of the population with two or more health insurance policies in the age groups younger than 19, 19–34, 35–64, and 65 and older; and proportion of population older than age 65 without any health insurance. Column (4) further adds distance to an airport with direct international flights to high-severity countries measured following the method in Desmet and Wacziarg (2021); Gini index and proportion of population below the poverty line; Social Capital Index; percentage of adult population with obesity; percentage of population who smoke as adults; 30-day risk-adjusted mortality rate; heart disease death rate; percentage of population diagnosed with diabetes among adults older than 20 years of age; contribution to percentage change in real GDP by private-services-providing industries, private-goods-providing industries, and government enterprises, and government enterprises; percentage change in the annual average employment for a given year; and the proportion of votes for Democrats in counties in the 2016 US presidential election as a proxy for pre-crisis political orientation. Predicted ancestral compositions in each county using the instruments from Burchardi et al. (2019) and the post-1500 World Migration Matrix of Putterman and Weil (2010) were used to adjust crop yield measures

**Table 4:** Impact of Long-Term Orientation on Mobility Proxies: Visitors to Hobby Centers, Restaurants, and Movie Theaters (except drive-ins)

Total visitors from county march to november 2020								
Panel A: hobby centers and gaming stores	(1)	(2)	(3)	(4)	(5)	(6)	(7)	(8)
	March	April	May	June	July	August	September	October
Pre-1500CE yield (Anc.)	$-$11.28	$-$12.58	$-$11.64	$-$12.00	$-$13.19	$-$12.63	$-$11.74	$-$11.75
	[6.357]*	[6.375]**	[7.488]	[6.163]*	[6.210]**	[6.348]**	[6.261]*	[6.404]*
	(4.211)***	(4.078)***	(5.410)**	(4.228)***	(4.188)***	(4.312)***	(4.228)***	(4.274)***
	$\langle p=0.125 \rangle$	$\langle p=0.081 \rangle$	$\langle p=0.139 \rangle$	$\langle p=0.087 \rangle$	$\langle p=0.071 \rangle$	$\langle p=0.081 \rangle$	$\langle p=0.105 \rangle$	$\langle p=0.107 \rangle$
Post-1500CE yield Ch. (Anc.)	$-$0.974	$-$1.039	$-$0.997	$-$0.996	$-$0.989	$-$1.008	$-$0.938	$-$0.991
	[0.224]***	[0.248]***	[0.328]***	[0.221]***	[0.213]***	[0.224]***	[0.219]***	[0.233]***
	(0.201)***	(0.223)***	(0.314)***	(0.197)***	(0.188)***	(0.206)***	(0.202)***	(0.215)***
	$\langle p=0.003 \rangle$	$\langle p=0.003 \rangle$	$\langle p=0.010 \rangle$	$\langle p=0.001 \rangle$	$\langle p=0.000 \rangle$	$\langle p=0.001 \rangle$	$\langle p=0.002 \rangle$	$\langle p=0.003 \rangle$
Observations	1,733	1,733	1,733	1,733	1,733	1,733	1,733	1,733
R-squared	0.864	0.824	0.801	0.855	0.856	0.851	0.855	0.849

Panel B: restaurants	(1)	(2)	(3)	(4)	(5)	(6)	(7)	(8)
Pre-1500CE yield (Anc.)	$-$5.351	$-$5.526	$-$6.976	$-$7.669	$-$9.034	$-$8.500	$-$7.737	$-$7.709
	[4.590]	[5.275]	[5.755]	[5.072]	[4.737]*	[4.805]*	[4.901]	[4.965]
	(3.705)	(4.297)	(4.600)	(3.981)*	(3.547)**	(3.621)**	(3.779)**	(3.828)**
	$\langle p=0.339 \rangle$	$\langle p=0.375 \rangle$	$\langle p=0.307 \rangle$	$\langle p= 0.239 \rangle$	$\langle p=0.137 \rangle$	$\langle p=0.167 \rangle$	$\langle p=0.210 \rangle$	$\langle p= 0.218 \rangle$
Post1500 CE yield Ch. (Anc.)	$-$1.032	$-$1.010	$-$1.058	$-$1.048	$-$1.028	$-$1.050	$-$1.008	$-$1.058
	[0.191]***	[0.224]***	[0.251]***	[0.205]***	[0.188]***	[0.197]***	[0.198]***	[0.208]***
	(0.171)***	(0.202)***	(0.226)***	(0.184)***	(0.166)***	(0.175)***	(0.178)***	(0.182)***
	$\langle p=0.000 \rangle$	$\langle p=0.003 \rangle$	$\langle p=0.001 \rangle$	$\langle p=0.001 \rangle$	$\langle p=0.000 \rangle$	$\langle p=0.000 \rangle$	$\langle p=0.001 \rangle$	$\langle p=0.000 \rangle$
Observations	1,824	1,824	1,824	1,824	1,824	1,824	1,824	1,824
R-squared	0.917	0.897	0.880	0.896	0.899	0.899	0.899	0.893

Panel C: movie theaters (except drive-INS)	(1)	(2)	(3)	(4)	(5)	(6)	(7)	(8)
Pre-1500CE yield (Anc.)	$-$3.712	3.955	3.621	$-$1.217	$-$0.583	$-$3.798	$-$7.399	$-$7.085
	[7.517]	[5.854]	[5.460]	[5.759]	[4.914]	[5.200]	[6.296]	[5.181]
	(5.148)	(4.854)	(4.757)	(5.778)	(4.912)	(4.140)	(4.359)*	(3.744)*
	$\langle p=0.598 \rangle$	$\langle p=0.525 \rangle$	$\langle p=0.512 \rangle$	$\langle p=0.824 \rangle$	$\langle p=0.903 \rangle$	$\langle p=0.423 \rangle$	$\langle p=0.248 \rangle$	$\langle p=0.187 \rangle$
Post-1500CE yield Ch. (Anc.)	$-$0.596	$-$0.609	$-$0.529	$-$0.380	$-$0.514	$-$0.677	$-$0.601	$-$0.632
	[0.297]**	[0.255]**	[0.231]**	[0.284]	[0.246]**	[0.214]***	[0.258]**	[0.209]***
	(0.251)**	(0.193)***	(0.200)***	(0.271)	(0.225)**	(0.175)***	(0.199)***	(0.168)***
	$\langle p=0.100 \rangle$	$\langle p=0.030 \rangle$	$\langle p=0.031 \rangle$	$\langle p=0.270 \rangle$	$\langle p=0.089 \rangle$	$\langle p=0.009 \rangle$	$\langle p=0.028 \rangle$	$\langle p=0.009 \rangle$
Observations	822	822	822	822	822	822	822	822
R-squared	0.812	0.781	0.787	0.779	0.806	0.814	0.796	0.796
Geographic	Y	Y	Y	Y	Y	Y	Y	Y
Neighbor mob & COVID-19 prev	Y	Y	Y	Y	Y	Y	Y	Y
Socio-economic, pol., health	Y	Y	Y	Y	Y	Y	Y	Y
State fixed effects	Y	Y	Y	Y	Y	Y	Y	Y

Standard errors are denoted as follows: [bootstrap standard errors account for clustering at the commuting zone level using the bootstrap command with the cluster option in STATA; that is, in this method, the sample drawn during each replication is a bootstrap sample of commuting zones] and (standard errors are adjusted for arbitrary spatial clustering using the acreg package written by Colella et al. (2019)). Stars $$^{*** (**)[*]}$$ indicate significance at the 0.01(0.05)[0.1] level. $$\langle$$*p*-values are generated by the wild cluster bootstrap method using the boottest command written by Roodman et al. (2019), clustered at the commuting zone level$$\rangle$$. Each column reports monthly estimates from separate regressions, following Eq. 1, which includes ancestry-adjusted pre-1500 CE crop yield and ancestry-adjusted post-1500 CE yield change and accounts for ancestry-adjusted crop growth cycle and its changes; unobserved state-varying attributes via state fixed effects; geographic factors that include average roughness of terrain, precipitation, temperature, and elevation; and county-level COVID-19 prevalence in neighboring counties in the month when the regression is estimated, which is measured as the confirmed COVID-19 cases per test per 100,000 averaged over all neighboring counties; COVID-19 tests per 100,000 conducted in the county in the month when the regression is estimated; mobility proxies in neighboring counties; dummy variables for the urban status of each county comprising large central or large fringe metro counties and medium metro and small metro counties; mean income; proportion of males; population density; proportion of population from a Black or African American, Native American, White, and Hispanic or Latino background; proportion of population using public transport; proportion of family and non-family households living in two or more unit structures; proportion of family and non-family households with three or more members; proportion of population in age groups younger than 19, 19–34, and 35–64 (with 65 and older as the omitted group); proportion working from home; proportion of population with an education level higher than or equal to the higher secondary level; healthcare coverage measured as the proportion of the population with two or more health insurance policies in the age groups younger than 19, 19–34, 35–64, and 65 and older, and the proportion of the population older than age 65 without any health insurance; distance to an airport with direct international flights to high-severity countries; Gini index and proportion of population below the poverty line; Social Capital Index; percentage of adult population with obesity; percentage of population who smoke as adults; 30-day risk-adjusted mortality rate; heart disease death rate; percentage of population diagnosed with diabetes among adults older than 20 years of age; contribution to the percentage change in GDP by private-services-providing industries, private-goods-providing industries, and government enterprises, and government enterprises; percentage change in annual average employment for a given year; and proportion of votes for Democrats in counties in the 2016 US presidential election. Predicted ancestral compositions in each county using the instruments from Burchardi et al. (2019) and the post-1500 World Migration Matrix of Putterman and Weil (2010) were used to adjust crop yield measures. Our outcome variables are total number of visitors from each county to POIs from the start of every month to the end of the month obtained from SafeGraph Patterns data. The POIs are hobby, toy, and games stores in panel A; restaurants, and other eating places (full-service restaurants, limited-service restaurants, cafeterias, grill buffets, and buffets, and snacks and non-alcoholic beverage bars) in panel B; and motion picture theaters (excluding drive-ins) in panel C. All variables are normalized by subtracting their mean and dividing by their standard deviation. Therefore, all coefficients are comparable and estimate the effect of a one standard deviation increase in the independent variable

**Table 5:** Impact of Long-Term Orientation on Mobility Proxies: Visitors to Fitness Centers and Clothing Stores

Total visitors from county march–november 2020								
Panel A fitness centers/gym	(1)	(2)	(3)	(4)	(5)	(6)	(7)	(8)
	March	April	May	June	July	August	September	October
Pre-1500CE yield (Anc.)	$-$8.221	$-$8.469	$-$11.49	$-$10.99	$-$12.14	$-$12.10	$-$11.81	$-$11.92
	[3.185]***	[3.298]**	[4.426]***	[3.919]***	[3.934]***	[4.092]***	[4.376]***	[4.292]***
	(1.763)***	(1.676)***	(2.544)***	(2.320)***	(2.709)***	(2.720)***	(2.720)***	(2.486)***
	$\langle p=0.0020 \rangle$	$\langle p=0.000 \rangle$	$\langle p=0.002 \rangle$	$\langle p=0.000 \rangle$	$\langle p=0.000 \rangle$	$\langle p=0.000 \rangle$	$\langle p=0.000 \rangle$	$\langle p=0.000 \rangle$
Post-1500CE yield Ch. (Anc.)	$-$1.035	$-$1.044	$-$1.130	$-$1.102	$-$1.065	$-$1.073	$-$1.030	$-$1.084
	[0.128]***	[0.135]***	[0.178]***	[0.139]***	[0.137]***	[0.141]***	[0.150]***	[0.153]***
	(0.0774)***	(0.0769)***	(0.138)***	(0.0886)***	(0.0955)***	(0.104)***	(0.109)***	(0.105)***
	$\langle p=0.000 \rangle$	$\langle p=0.000 \rangle$	$\langle p=0.000 \rangle$	$\langle p=0.000 \rangle$	$\langle p=0.000 \rangle$	$\langle p=0.000 \rangle$	$\langle p=0.000 \rangle$	$\langle p=0.000 \rangle$
Observations	1,800	1,800	1,800	1,800	1,800	1,800	1,800	1,800
R-squared	0.929	0.925	0.896	0.905	0.913	0.910	0.905	0.900

Panel B clothing stores	(1)	(2)	(3)	(4)	(5)	(6)	(7)	(8)
Pre-1500CE yield (Anc.)	$-$10.21	$-$11.39	$-$13.88	$-$12.69	$-$13.02	$-$12.75	$-$12.14	$-$11.95
	[3.710]***	[3.731]***	[5.332]***	[3.893]***	[3.662]***	[3.878]***	[4.038]***	[3.856]***
	(2.996)***	(3.189)***	(4.325)***	(2.950)***	(2.902)***	(2.931) ***	(3.072)***	(2.953)***
	$\langle p=0.0430 \rangle$	$\langle p=0.005 \rangle$	$\langle p=0.050 \rangle$	$\langle p=0.013 \rangle$	$\langle p=0.001 \rangle$	$\langle p=0.017 \rangle$	$\langle p= 0.020 \rangle$	$\langle p=0.025$
Post-1500CE yield Ch. (Anc.)	$-$1.220	$-$1.270	$-$1.323	$-$1.230	$-$1.191	$-$1.197	$-$1.171	$-$1.234
	[0.142]***	[0.152]***	[0.263]***	[0.146]***	[0.142]***	[0.159]***	[0.157]***	[0.157]***
	(0.116)***	(0.110)***	(0.250)***	(0.123)***	(0.111)***	(0.140)***	(0.135)***	(0.132)***
	$\langle p=0.000 \rangle$	$\langle p=0.000 \rangle$	$\langle p=0.000 \rangle$	$\langle p=0.000 \rangle$	$\langle p=0.000 \rangle$	$\langle p=0.000 \rangle$	$\langle p=0.000 \rangle$	$\langle p=0.000 \rangle$
Observations	1,752	1,752	1,752	1,752	1,752	1,752	1,752	1,752
R-squared	0.932	0.916	0.882	0.922	0.918	0.915	0.919	0.920
Geographic	Y	Y	Y	Y	Y	Y	Y	Y
Neighbor mob & COVID-19 prev	Y	Y	Y	Y	Y	Y	Y	Y
Socio-economic, pol., health	Y	Y	Y	Y	Y	Y	Y	Y
State-fixed effects	Y	Y	Y	Y	Y	Y	Y	Y

Standard errors are denoted as follows: [bootstrap standard errors account for clustering at the commuting zone level using the bootstrap command with the cluster option in STATA; that is, in this method, the sample drawn during each replication is a bootstrap sample of commuting zones] and (standard errors are adjusted for arbitrary spatial clustering using the acreg package written by Colella et al. (2019)). Stars $$^{*** (**)[*]}$$ indicate significance at the 0.01(0.05)[0.1] level. $$\langle$$*p*-values are generated by the wild cluster bootstrap method using the boottest command written by Roodman et al. (2019), clustered at the commuting zone level$$\rangle$$. Each column reports monthly estimates from separate regressions, following Eq. 1, which includes ancestry-adjusted pre-1500 CE crop yield and ancestry-adjusted post-1500 CE yield change and accounts for ancestry-adjusted crop growth cycle and its changes; unobserved state-varying attributes via state fixed effects; geographic factors that include average roughness of terrain, precipitation, temperature, and elevation, and county-level COVID-19 prevalence in neighboring counties in the month when the regression is estimated, which is measured as the confirmed COVID-19 cases per test per 100,000 averaged over all neighboring counties; COVID-19 tests per 100,000 conducted in the county in the month when the regression is estimated; mobility proxies in neighboring counties; dummy variables for the urban status of each county comprising large central or large fringe metro counties and medium metro and small metro counties; mean income; proportion of males; population density; proportion of population from a Black or African American, Native American, White, and Hispanic or Latino background; proportion of population using public transport; proportion of family and non-family households living in two or more unit structures; proportion of family and non-family households with three or more members; proportion of population in age groups younger than 19, 19–34, and 35–64 (with 65 and older as the omitted group); proportion working from home; proportion of population with an education level higher than or equal to the higher secondary level; healthcare coverage measured as the proportion of the population with two or more health insurance policies in the age groups younger than 19, 19–34, 35–64, and 65 and older, and the proportion of the population older than age 65 without any health insurance; distance to an airport with direct international flights to high-severity countries; Gini index and proportion of population below the poverty line; Social Capital Index; percentage of adult population with obesity; percentage of population who smoke as adults; 30-day risk-adjusted mortality rate; heart disease death rate; percentage of population diagnosed with diabetes among adults older than 20 years of age; contribution to the percentage change in GDP by private-services-providing industries, private-goods-providing industries, and government enterprises, and government enterprises; percentage change in annual average employment for a given year; and proportion of votes for Democrats in counties in the 2016 US presidential election. Predicted ancestral compositions in each county using the instruments from Burchardi et al. (2019) and the post-1500 World Migration Matrix of Putterman and Weil (2010) were used to adjust crop yield measures. Our outcome variables are total number of visitors from each county to POIs from the start of every month to the end of the month obtained from SafeGraph Patterns data. The POIs are fitness and recreational centers in panel A and clothing stores in panel B. All variables are normalized by subtracting their mean and dividing by their standard deviation. Therefore, all coefficients are comparable and estimate the effect of a one standard deviation increase in the independent variable

## Supplementary Information

Below is the link to the electronic supplementary material.

Supplementary file 1 (pdf 386 KB)

## Footnotes

1 Spolaore and Wacziarg (2009); Spolaore and Wacziarg (2013); Alesina et al. (2013); Spolaore and Wacziarg (2016); Galor and Özak, (2016); Falk et al. (2018); Galor and Savitskiy, (2018); Becker et al. (2020).

2 For example, patience positively affects the adherence to physical activity advice (Van Der Pol et al., 2017) Recent studies have shown that economic preferences are correlated with compliance with COVID-19-specific policy measures (Chan et al., 2020; Müller & Rau, 2021).

3 We use the terms long-term orientation traits, future-oriented mindsets, and future-oriented outlooks interchangeably.

4 In the US, adverse health shocks are known to be a major source of economic risk resulting in persistent declines in earnings, substantially reduced consumer borrowing and access to credit and, increasing bankruptcy rates (Dobkin et al., 2018).

5 This is explained in Sect. 4.

6 Chan et al. (2020); Müller and Rau (2021).

7 Dohmen et al. (2018).

8 Borghans et al. (2008) suggest that personality traits are predictive of socio-economic success and are more malleable after early childhood years than cognitive skills.

9 Research on the effects of the history of locations is extensive, important examples follow. Hibbs and Olsson (2004) show that geographical factors correlated with the timing of the Neolithic Revolution in a region predicted income and institutions in 1997. Comin et al. (2010) show that contemporary output can be determined by the state of technology in a country 500, 2000, or even 3000 years ago. Bockstette et al. (2002) find that an index of the presence of state-level political institutions from year 1 to 1950 has positive correlations with both 1995 income and 1960–95 income growth. Galor and Moav (2007) provide empirical evidence for a link from the timing of the transition to agriculture to current variations in life expectancy. Michalopoulos (2012) shows that regional variation in land quality and elevation is a key determinant of present-day linguistic diversity. Durante (2009) show that variation in social trust is determined by historical variability in weather patterns.

10 The evolutionary forces that determine time discounting and its impact on human behavior have been examined in Loewenstein and Elster (1992), Frederick et al. (2002), and Rosati et al. (2007).

11 Fernández and Fogli (2009) show that the fertility outcomes of the children of immigrants are determined by the fertility rates of their parents’ country of origin. Fulford et al. (2020) find that the cultural norms and human capital that migrants in the US have passed on to their children are positively correlated with local development. Dohmen et al. (2012) highlight the role of the socialization process in the intergenerational transmission of attitudes.

12 The CSI ensures the comparability of crop yields by measuring the nutritional differences across crops. See Appendix-A.1 for Data details.

13 For illustrative purposes, let us consider the ancestral composition of Afghanistan in the migration matrix as follows:

$$\begin{aligned} \begin{matrix} afg&{}irn&{}tkm&{}uzb\\ 0.87&{}0.01&{}0.03&{}0.09\\ \end{matrix} \end{aligned}$$

The matrix depicts that in 1500 CE, 87% of the present-day population of Afghanistan lived within the present-day borders of Afghanistan, 1% of the present-day population of Afghanistan lived within the present-day borders of Iran, 3% of the present-day population of Afghanistan lived within the present-day borders of Turkmenistan, and 9% of the present-day population of Afghanistan lived within the present-day border of Uzbekistan.

14 For example, the pre-1500 CE crop yield measure (measured in million calories per hectare per year) of Afghanistan adjusted for 1500 CE ancestry is as follows: proportion of people from Afghanistan who lived in Afghanistan in 1500 CE $\times$ pre-1500 CE crop yield in Afghanistan + the proportion of people from Afghanistan who lived in Iran in 1500 CE $\times$ pre-1500 CE crop yield of Iran + the proportion of people from Afghanistan who lived in Turkmenistan in 1500 CE $\times$ pre-1500 CE crop yield of Turkmenistan + proportion of people from Afghanistan who lived in Uzbekistan in 1500 CE $\times$ pre-1500 CE crop yield of Uzbekistan.

15 See Appendix Sect. A.2 for details on the ancestry IV developed in Burchardi et al. (2019).

16 As an example, suppose ancestry IV in Burchardi et al. (2019) predicts that the residents of US county *d* have ancestors from Italy and Argentina. The ancestry-adjusted pre-1500 CE crop yield measure for county *d* is as follows: composition of Italian ancestry in county *d* as predicted by the ancestry IV in Burchardi et al. (2019) $\times$ pre-1500 CE crop yield in Italy adjusted for ancestry in 1500 C.E. using Putterman and Weil (2010) migration matrix $+$ composition of Argentinian ancestry in county *d* as predicted by the ancestry IV in Burchardi et al. (2019) $\times$ pre-1500 CE crop yield in Argentina adjusted for ancestry in 1500 C.E. using Putterman and Weil (2010) migration matrix.

17 Appendix Fig. A.3 depicts the percentage change in testing per 100,000 in the US in 2020 relative to total tests per 100,000 in March 2020.

18 Testing data from January to March 15, 2020 are zero for 95% of observations. Absolute tests performed in each county from the Centers for Disease Control and Prevention (CDC) are available only for selected states and counties. We use tests per 100,000 data from the CDC that are available for most counties.

19 The county-level data on total deaths are available from the CDC by US county of residence and age group from January 1, 2020 to December 31, 2020. The data are reported in age ranges: 0–19, 20–24, 25–29, 30–34, 35–49, 50–54, 55–59, 60–64, 65–69, 70–74, and 75 and above. As a result of the CDC’s suppression policy, whereby deaths fewer than 10 are suppressed and deaths fewer than 20 are reported as unreliable, deaths among younger age cohorts are missing for most counties. Therefore, our excess deaths analysis sample that includes counties reporting total deaths for the 45 and above age group (by aggregating total deaths in five age intervals) is smaller than the analysis sample that includes deaths in the 55 years and above age group, and so on.

20 These data sets have also been used in several published studies, such as in Weill et al. (2020); Bargain and Aminjonov (2020); Cot et al. (2021).

21 See Appendix A.6 for details.

22 The baseline day is taken as the median value of the same day of the week between January 3 and February 6. The data are reported as a positive or negative percentage. See Appendix Sect. A.7 for more details.

23 A national emergency was declared in the US on March 13, 2020. Therefore, our monthly data from the Google Community Mobility Reports were generated with the 15th of every month as the starting date. This was not possible with the SafeGraph data because the monthly data that we collected from SafeGraph begin from the start of each month. Therefore, the analysis of SafeGraph data is until the end of October.

24 Google Community Mobility Reports categories include percentage changes in visits or length of stay in places classified as retail and recreation, grocery stores and pharmacies, parks, transit stations, workplaces, and residential within a geographic area. We do not use mobility data on workplaces, grocery stores and pharmacies, and transits because they measure essential travel and are confounded by the mobility behavior of county non-residents. Further, we do not use park visits because they do not largely capture noncompliance with social distancing given that parks have open spaces where people could potentially maintain social distancing.

25 Jay et al. (2020); Lakbar et al. (2020); Karaca-Mandic et al. (2021); Viner et al. (2021); Monod et al. (2021); Merow and Urban (2020); Grossman et al. (2020); Desmet and Wacziarg (2021).

26 Grossman et al. (2020).

27 Desmet and Wacziarg (2021) also point out that the share of residents in nursing homes is an important correlate for county-level COVID-19 severity. The data on the average number of daily residents in nursing homes are from Provider Info, published by the Centers for Medicare & Medicaid Services (same source as Desmet and Wacziarg 2021). We show that our results are robust to including the share of nursing home residents in Appendix Table B.2.

28 Merow and Urban 2020

29 Conventional methods of inference are not based on estimated treatment variables and hence leads to incorrect standard errors.

30 Colella et al. (2019) use the cluster data structure of Conley (1999) by specifying a circle around each unit that specifies how distance dependence is likely to occur, allowing for possible decay (or not) in interdependence.

31 Cinelli et al. (2020) provide a brief review of the latest developments in the bad controls literature.

32 In all our specifications, we control for COVID-19 cases in neighboring counties. The weekly-level data on COVID-19 cases in the initial phase of the pandemic in 2020 can potentially have significant measurement errors due to inadequate testing technology. Cumulative data at the monthly level can include COVID-19 cases that are likely to be missed in the initial weeks since COVID-19 testing became more widely available over time.

33 These include socio-economic, underlying health conditions and behavior, political partisanship, geographical, contributions to the percent change in real GDP by industry and changes in employment, mobility and infection prevalence in neighboring counties, and time-invariant state-varying effects.

34 Comprising of pre-existing county-level hospital capacity (includes proportion of full-time staff, beds available, hospitals that were short term, hospitals operated by the Catholic Church, critical access hospitals, rural referral centers, and sole community providers; proportion with DNV Healthcare accreditation; and proportion of hospitals with a Center for Improvement in Healthcare Quality accreditation) and types of hospitals reporting (comprising the proportion of critical access hospitals reporting, children’s hospitals reporting, and short-term hospitals).

35 We do not have hospital data for the earlier phases of the pandemic to be able to estimate the trajectory of the causal difference with regard to the hospital utilization variables.

36 Galor and Özak (2016) find that the effect of crop yield on LTO is strongest in the Old World sample, where post-1500 CE migration is not a concern, ruling out sorting as a potential channel.

37 Google Community Mobility Report data are a percentage change relative to the five-week period January 3 to February 6. Therefore, we do not have pre-intervention data at the monthly level in this case. We cannot reliably test for violations in pretrends for data measuring COVID-19 severity because reliable data are unavailable for the months before the declaration of a national emergency due to the pandemic.

38 In the case of the proxy measure of visitors to fitness centers, there is a marginal difference across high and low LTO counties under the post-1500 CE yield change definition.
