# Supplementary material for: The Impact of Long–Term Orientation Traits on Pandemic Fatigue Behavior: Evidence from the Columbian Exchange
Source: J Econ Growth (Boston). 2022 Oct 27:1–42. Online ahead of print. doi: 10.1007/s10887-022-09218-0 (PMC9612631; doi:10.1007/s10887-022-09218-0)
Supplement: Supplementary file 1 — (pdf 386 KB) [file 10887_2022_9218_MOESM1_ESM.pdf]

THE IMPACT OF LONG–TERM ORIENTATION TRAITS ON PANDEMIC FATIGUE BEHAVIOR: EVIDENCE FROM THE COLUMBIAN EXCHANGE (ONLINE APPENDIX)

SUTANUKA ROY[1], SUDHIR GUPTA[2], AND RABEE TOURKY[3]

Contents

Appendix A. 2
A.1. DATA ON CROP YIELD MEASURES 2
A.2. ANCESTRY-IV DATA 3
A.3. COVID-19 Testing Trends 6
A.4. EXCESS DEATHS 7
A.5. HOSPITAL CAPACITY UTILIZATION OUTCOMES 7
A.6. SafeGraph PATTERNS DATA 7
A.7. GOOGLE MOBILITY DATA 8
A.8. ADDITIONAL VARIABLES 8
A.9. COVID CASES AND DEATHS 12
Appendix B. Tables 13
Appendix C. Towards an understanding of the mechanism of evolution of time preferences 50
References 51

[1]Research School of Economics, Australian National University; sutanuka.roy@anu.edu.au
[2]Research School of Economics, Australian National University; sudhir.gupta@anu.edu.au
[3]Research School of Economics, Australian National University; rabee.tourky@anu.edu.au

Appendix A.

A.1. **DATA ON CROP YIELD MEASURES.** The data used to construct the Caloric Suitability Index (CSI) are from the Global Agro-Ecological Zones (GAEZ) project of the Food and Agriculture Organization (FAO), which provides global estimates of crop yield and crop growth cycle for a spectrum of crops in grids with $5' \times 5'$ cell size (i.e., approximately 100 km^2). The measures capture potential crop yield and growth cycle in the early stages of human development, which were marked by low levels of inputs and rain-fed agriculture and occurred under agroclimatic conditions that were independent of human intervention, thus removing potential concerns about agricultural investment reflecting endogenous choices that may be correlated with LTO traits. For further details on the construction of the CSI, please refer to Galor and Özak (2015), Galor and Özak (2016); Galor, Özak and Sarid (2016). We obtain the data on pre-1500 CE and post-1500 CE maximum potential crop yield, crop growth cycle and their changes at the level of $5' \times 5'$ cell size from Galor and Özak (2015); Galor, Özak and Sarid (2016); Galor and Özak (2016).

A.2. **ANCESTRY-IV DATA.** Data used in the construction of the instruments were obtained from the individual files of the Integrated Public Use Microdata Series (IPUMS) samples of the 1880, 1900, 1910, 1920, 1930, 1970, 1980, 1990, and 2000 waves of the U.S. census, and the 2006–2010 five-year sample of the American Community Survey. The residence of the respondents are recorded at the level of historic counties, and at the level of historic county groups or Public Use Microdata Areas (PUMAs) from 1970 onwards. Burchardi, Chaney and Hassan (2019) use contemporaneous population weights, whenever necessary, to transition data from the historic county group or PUMA level to the historic county, and then use area weights to transition data from the historic county level to the 1990 U.S. county level. Further, when respondents reported ancestry cannot be directly mapped to foreign countries in their 1990 borders (e.g. "Basque"), Burchardi, Chaney and Hassan (2019) use population or area weights to map data from the answer-level to the 1990 foreign country level.

A.2.1. *Instruments for Ancestry.* The IV strategy in Burchardi, Chaney and Hassan (2019) is based on a simple reduced-form dynamic model of migration, where the migration from a given origin country o to a given US destination county d in period t depends on the total number of migrants arriving in the US from o (push factor), the relative attractiveness of county d to migrants arriving at time t (pull factor), and the size of the pre-existing local population of ancestry of origin country o in county d at time t. The model shows that the number of contemporary residents in the US county d who are descendants of migrants from origin country o is a function of interactions of the sequence of push-and-pull factors.

Therefore, the IV is constructed from this sequence of interactions by isolating the variation in the push-and-pull factors that is plausibly independent of unobservables that could make a given county within the US more attractive to migrants from a given origin country. The pull factor from origin country o to destination county d is measured as the fraction of migrants who come from countries that are not in the continent of origin country o. Thus, the pull factor is determined by the migrants arriving in the US at time t from the set of continents that excludes the continent of origin country o. The push factor is the total number of migrants arriving in the US from origin country o at time t who settled outside the region of county d. The instrument for the present-day number of residents in county d with ancestors from origin country o is the full set of simple and higher order interactions of push-and-pull factors going back to 1880 (see Section 3 in Burchardi, Chaney and Hassan, 2019 for details about the ancestry-IV).

We use the replication data and codes from Burchardi, Chaney and Hassan (2019) that involve more than 70 million immigrants, including first-generation and second-generation immigrants who were alive in 1880, to predict ancestral composition in US counties in 2010 via the following equation at the county-country level (which is a variant of Equation 4 and corresponds to column (7) of Table-2 in Burchardi, Chaney and Hassan, 2019).

$$LogAncestry_{o,d}^{2010} = \delta_o + \delta_d + \delta_{o,r(d)} + \delta_{c(o),d} + \sum_{t=1880}^{2010} \alpha_t I_{o,-r(d)}^{t} \frac{I_{-c(o),d}^{t}}{I_{-c(o)}^{t}} + \sum_{n=1}^{5} \delta_n PC_n + X_{o,d}'\gamma + \eta_{o,d} \tag{1}$$

where $\sum_{n=1}^{5} \delta_n PC_n$ is the sum of the first five principal components that have the information contained in 758 higher-order terms of push and pull interactions until 2010. δ_o and δ_d are origin and destination fixed effects. $\delta_{o,r(d)}$ is origin-country $\times$ destination-census-region fixed effects. $\delta_{c(o),d}$ is continent-of-origin $\times$ destination-county fixed effects. Standard errors are clustered at the origin-country level. The dependent variable $LogAncestry_{o,d}^{2010}$ is the log one plus the number of individuals in the county in 2010 who report to having ancestors in the origin country

as their first ancestry. $I^t_{o,-r(d)}$ denotes the total number of migrants arriving in the United States (US) from the origin-country o who reside in locations not in the census region of d at time t. $\frac{I^t_{-c(o),d}}{I^t_{-c(o)}}$ denotes the fraction of migrants coming from countries outside the continent-of-origin country o to location d at time t. $X'_{o,d}$ includes controls such as geographic distance between origin and destination and difference in latitude between o and d.

The following section explains the construction of the ancestry-adjusted measures in detail.

A.2.2. *Ancestry-Adjusted Measures Further Details on Implementation.* To construct the ancestry-adjusted measure, we must merge three data sets: 1) country-level agricultural measures from Galor and Özak (2016); 2) predicted ancestral composition for 165 countries from the World Migration Matrix, 1500–2000 in Putterman and Weil (2010); and 3) predicted ancestral composition for US counties from Burchardi, Chaney and Hassan (2019). We use International Standards Organization (ISO) codes as the merging variable for the agricultural data set from Galor and Özak (2015), Galor, Özak and Sarid (2016), Galor and Özak (2016), and the World Migration Matrix, 1500–2000 developed.[1] The data on ancestral composition in US counties in Burchardi, Chaney and Hassan (2019) did not have ISO three-digit alphabetic codes (ISO codes). We use data on numeric and alphabetic country codes from M49 standard developed by the United Nations Statistics Division and merge them with the ancestral composition in the US county data in Burchardi, Chaney and Hassan (2019). We then use the ISO codes to merge the ancestry data predicted by Burchardi, Chaney and Hassan (2019) with the data of agricultural measures that have been adjusted for year 1500 CE ancestry following the method in Galor and Özak (2016).

The ancestry data in Burchardi, Chaney and Hassan (2019) has countries with 1990 year boundaries, and the crop yield data adjusted for ancestry using the migration matrix developed in Putterman and Weil (2010) have post-1990 year country boundaries.[2] We account for boundary changes that occurred because of the splitting of countries post-1990. In the crop yield data, which have been adjusted for the year 1500 CE ancestry following Galor and Özak (2016), we add entries for parent countries that disintegrated after 1990. These countries include Belgium/Luxembourg (BLU), which was disintegrated into Belgium and Luxembourg; Czechoslovakia (CZS), which was disintegrated into the Czech Republic and Slovakia; Socialist Yugoslav (SYV), which was disintegrated into Yugoslavia, Bosnia and Herzegovina, Croatia, North Macedonia, and Slovenia; and the Union of Soviet Socialist Republics (USR), which was disintegrated into Armenia, Azerbaijan, Belarus, Estonia, Georgia, Kazakhstan, Kyrgyzstan, Latvia, Lithuania, Moldova, Russia, Turkmenistan, Tajikistan, Uzbekistan, and Ukraine.

The crop yields for these parent countries are computed as the average crop yield of the individual countries. The crop yield measure is adjusted for ancestry in the year 1500 CE for parent country Belgium/Luxembourg (BLU) = (crop measures adjusted for year 1500 CE ancestry for Belgium + the crop measures adjusted for year 1500 CE ancestry for Luxembourg)/2.

For the entries of the parent countries, the ISO codes are the following: i) for the Union of Soviet Socialist Republics, we add USR as the three-letter code and 810 as the numeric code; ii) for Socialist Yugoslav, we add SYV as the three-letter code and 890 as the numeric code; iii) for Belgium/Luxembourg, we add BLU as the three-letter code and 918 as the numeric code; and iv) for Czechoslovakia, we add CZS as the three-letter code and 200 as the

[1] ISO codes are updated in the migration matrix as follows: ZAR to COD, CIL to COK, TMP to TLS, ROM to ROU, and OAN to TWN.

[2] The main appendix to the World Migration Matrix, 1500–2000 states that the matrix should be interpreted as providing information on where the ancestors of each country's present population were living in 1500, where "present" refers to the 1990s and early 2000s.

numeric code. These data on crop yield measures with ancestry adjustment following Galor and Özak (2016) are merged with data on predicted ancestry composition in US counties in Burchardi, Chaney and Hassan (2019).[3]

[3]The following 46 countries were dropped while merging because they were not available in the World Migration Matrix, 1500–2000 in Putterman and Weil (2010): Andorra, Antigua and Barbuda, the Bahamas, Barbados, Bermuda, Solomon Islands, British Virgin Islands, Brunei, Cayman Islands, Dominica, Faroe Islands, Falkland Islands, French Guiana, French Polynesia, Djibouti, Palestine, Gibraltar, Kiribati, Greenland, Grenada, Guadeloupe, Liechtenstein, Maldives, Martinique, Monaco, Montserrat, Nauru, Aruba, New Caledonia, Vanuatu, Norfolk Island, Pitcairn Islands, Reunion, Saint Helena, Saint Kitts and Nevis, Anguilla, Saint Lucia, Saint Pierre and Miquelon, San Marino, Seychelles, Western Sahara, Suriname, Tokelau, Turks and Caicos Islands, Tuvalu, Wallis, and Futuna.

A.3. **COVID-19 Testing Trends.**

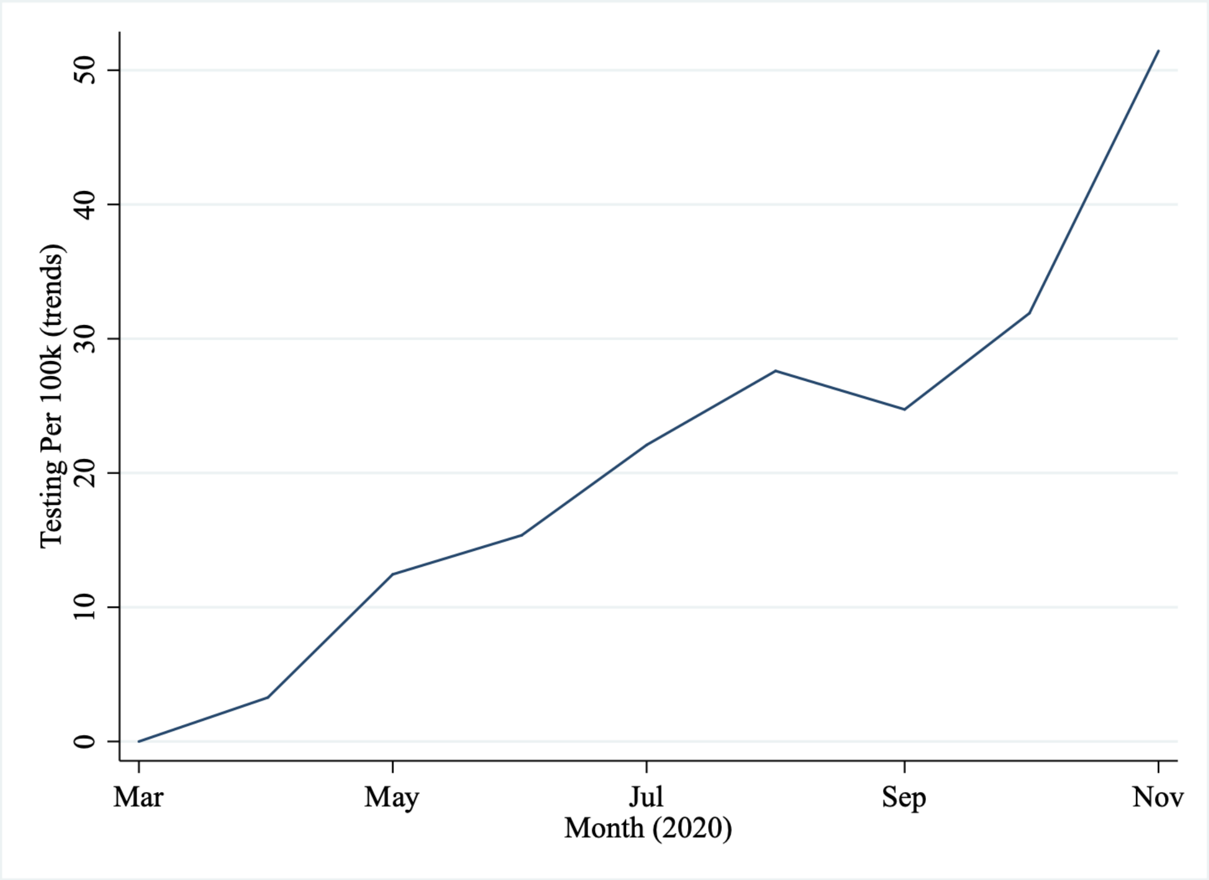

Notes: The figure plots trends in total testing per 100,000, normalized relative to the monthly value of March 2020.

A.4. **EXCESS DEATHS.** To compute excess deaths, we obtain data on the multiple causes of death for the years 2019 and 2018 from the Multiple Cause of Death data from the CDC's Division of Vital Statistics of the National Center for Health Statistics. This database is used to compute expected total deaths in the excess death calculation. In the database, county-level data representing fewer than 10 people (0–9) are suppressed. The entries are marked as "unreliable" when the death count is fewer than 20. The data we use in our analysis contain mortality and population counts for all US counties by age. They are based the on the death certificates for US residents that contain a single underlying cause of death and up to 20 additional multiple causes. We use the count of total deaths in each county and population counts for the years 2018 and 2019. Source link: https://wonder.cdc.gov/mcd-icd10.html.

We obtain data on total deaths in the year 2020 at the county level from January 1, 2020 to December 31, 2020 from the CDC. The database is from the CDC's AH Provisional COVID-19 Deaths by County and Age for 2020. Source link: https://healthdata.gov/dataset/AH-Provisional-COVID-19-Deaths-by-County-an d-Age-f/ump8-fkea.

All entries where data are considered unreliable and all suppressed data are re-coded as missing.

A.5. **HOSPITAL CAPACITY UTILIZATION OUTCOMES.** We obtain facility-level data for hospitalization aggregated on a weekly basis (Friday to Thursday) from the document "COVID-19 Reported Patient Impact and Hospital Capacity by Facility," published by the US Department of Health and Human Services, accessed September 27, 2021. There are two main sources of this data: (1) HHS TeleTracking and (2) reporting provided directly to HHS Protect by state/territorial health departments on behalf of its healthcare facilities. The sample consists of only those counties that have hospitals registered with the Centers for Medicare & Medicaid Services (CMS) as of June 1, 2020. The hospital sample excludes psychiatric, rehabilitation, Indian health service facilities, US Department of Veterans Affairs facilities, defense health agency facilities, and religious non-medical facilities.

Our facility-level data are seven-day sum available at the weekly level for the period July 31, 2020 to November 30, 2020. We first compute the average seven-day sum of inpatient beds used per hospital by taking the average of the seven-day sum for the period July 31, 2020 to November 30, 2020 for each facility. Next, we aggregate the facility-level data to the county level by taking the average over all the facility-level values in the given county. To analyze inpatient bed use at the CZ level, we aggregate the county-level average inpatient bed occupancy to the CZ level by taking the average over all county-level values for a given CZ. In the case of the hospitalization from COVID-19 measure, we first compute total hospitalization from COVID-19 at the facility level by summing up the seven-day sum over the period July 31, 2020 to November 30, 2020. Then, we aggregate the facility-level data to the county level by taking the sum over all the facility-level values in the given county. To obtain total hospitalization from COVID-19 at the CZ level, we aggregate the county-level total to the CZ level by taking the sum over all county-level values for a given CZ.

In the data, suppression is applied to the file for variable entries less than four. In these cases, the field is replaced with "–999,999"; we re-code the –999,999 entries as missing.
Source link: https://healthdata.gov/Hospital/COVID-19-Reported-Patient-Impact-and-Hospital-Capa/ anag-cw7u.

A.6. **SafeGraph PATTERNS DATA.** SafeGraph primarily collects GPS location data such as latitude/longitude coordinates of a device along with a timestamp, and mobile ad identifiers, primarily using Apple iOS IDFAs or

Google Android IDs from third-party data partners such as mobile application developers and companies that aggregate information from those developers' mobile apps. We use the SafeGraph's Patterns dataset that contains information from 45 million mobile devices and various smartphone applications on traffic patterns to various categories of POIs, which allows us to measure the frequency, duration, and direction of visits and the home locations of visitors among many other factors relating to mobility behavior.

We collect monthly level visiting pattern data from SafeGraph's Pattern dataset for POIs under the recreation categories comprising restaurants, clothing centers, hobby centers and gaming stores, fitness and recreational sports centers, and movie theaters (excluding drive-ins). We use the variable visitor_home_cbg, which is defined as the total number of visitors to the POI from each census block group based on the visitor's home location. The home location is determined by SafeGraph through analyzing six weeks of data during nighttime hours (between 6:00 pm and 7:00 am). The sufficient amount of evidence (total data points and distinct days) is collected to assign a home (common nighttime) geohash-7 for the device, which is then mapped to a census block group.

We next measure counts of visitors from each county by summing the total number of visitors from all census block groups within each county. We also calculate alternative measures as suggested by SafeGraph to measure visits by home county, which is a product of total visitors from each county and the average visits per visitor in a particular POI in each county. To compute average visits per visitor at the county level, we use variable raw_visit_counts and raw_visitor_counts aggregated from the census block group level to the county level. The variable raw_visit_counts measures total number of visits to a given POI during a given period, and the variable raw_visitor_counts measures the total number of unique visitors to a given POI during a given period. We aggregate census block group data to the county level for a given POI in a given period by summing all visits and visitors to the given POI in the given period within the county. The county-level average visits per visitor is calculated as the ratio of county-level raw_visit_counts and raw_visitor_counts.

We accessed the data on restaurants on June 7, 2021, clothing stores on July 1, 2021, and hobby centers, gaming stores, fitness centers, and theaters on July 3, 2021.

Source link: https://docs.safegraph.com/docs/monthly-patterns

A.7. **GOOGLE MOBILITY DATA.** We obtain mobility measures at the US county level from Google Community Mobility Reports. Based on anonymized and aggregated data from Google users who have opted into the "Location History" service, each mobility measure records the percentage change in visits or length of stay in places classified as retail and recreation, grocery and pharmacy, parks, transit stations, workplaces, and residential places within a geographic area. The percentage change is compared with the median value of the same day of the week between January 3, 2020 and February 6, 2020. For privacy reasons, Google censors values if traffic volumes are not sufficiently high to ensure anonymity. We obtain data on the percentage change in the length of stay in residential places and percentage change in visits to retail and recreational centers. The data were accessed on September 27, 2021. Source link: https://www.google.com/covid19/mobility/

A.8. **ADDITIONAL VARIABLES.**

A.8.1. *PERCENTAGE OF SMOKERS.* The age-adjusted percentage of adult (aged $\geq$ 18 years) smokers in 2018 provided by County Health Rankings and Roadmaps. Source: https://www.countyhealthra nkings.org/explore-health-rankings.

A.8.2. *PERCENTAGE OF ADULT OBESITY.* The age-adjusted percentage of obesity among adults (aged $\geq$ 18 years) in 2018 provided by County Health Rankings and Roadmaps.
Source link: https://www.countyhealthra nkings.org/explore-health-rankings.

A.8.3. *DIABETES PERCENTAGE.* The estimated age-adjusted percentage of diagnosed diabetes in 2017 among the adult population aged 20 years and older .
Source link: https://gis.cdc.gov/grasp/diabetes/DiabetesAtlas.html.

A.8.4. *HEART DISEASE MORTALITY.* County-level estimates of mortality rates per 100,000 (all ages, all races/ethnicities, both genders, 2017–2019) from all heart diseases. The data are obtained from the CDC. All entries with –1 and –9999 are encoded as missing.
Source link: https://nccd.cdc.gov/DHDSPAtlas/.

A.8.5. *30-DAY RISK-ADJUSTED MORTALITY.* 30-day risk-adjusted mortality rate for heart attacks, heart failure, and pneumonia obtained from Bergeron et al. (2016).
Source link: https://opportunityinsights.org/data/.

A.8.6. *SOCIAL CAPITAL.* Created using principal component analysis (PCA) on voter turnout, census response rate in 2014, and associations and organizations obtained from Rupasingha, Goetz and Freshwater (2006).
Source link: https://aese.psu.edu/nerc rd/community/social-capital-resources.

A.8.7. *RACE.* The five-year (2015–2019) average estimates of the proportion of the White alone population, Black or African American alone population, Native American population, and Hispanic or Latino population, obtained from the US Census Bureau.

A.8.8. *EDUCATION.* The five-year (2015–2019) average estimates of the proportion of the population with an education level higher than or equal to a higher secondary level, obtained from the US Census Bureau.

A.8.9. *INCOME.* The five-year (2015–2019) average estimates of mean income, obtained from the US Census Bureaus' American Community Survey (ACS).

A.8.10. *WORK FROM HOME.* The proportion of the population working from home, obtained from the US Census Bureaus' ACS.

A.8.11. *HOUSING ARRANGEMENT.* The five-year (2015–2019) average estimates of the proportion of family and non-family households living in two or more unit structures and the proportion of family and non-family households with three or more members, obtained from the US Census Bureau.

A.8.12. *HEALTH CARE COVERAGE.* The five-year (2015–2019) average estimates of the proportion of the population with two or more health insurance policies in the age groups younger than 19, 19–34, 35–64, and 65 and older, and the proportion of people older than 65 years of age without any health insurance. The data are obtained from the US Census Bureau.

A.8.13. *POVERTY RATE.* The fraction of the population living below the poverty line as recorded by the 2000 Census SF3 Sample Data Table P087.
Source: https://opportunityinsights.org/data/.

A.8.14. *POPULATION AND AGE.* The five-year (2015–2019) average estimates of the total population and the population in the age groups younger than 19, 19–34, 35–64, and 65 and older. The data are obtained from the US Census Bureau.

A.8.15. *DENSITY.* The five-year (2015–2019) average estimates of the total population divided by the land area, obtained from the US Census Bureau.

A.8.16. *METRO COUNTY.* The classification of a county as a large central metro county, large fringe metro county, medium metro county, or small metro county. Source: National Center for Health Statistics. Urban-Rural Classification Scheme for Counties 2013.

A.8.17. *PUBLIC TRANSPORTATION.* The five-year (2015–2019) average estimates of the proportion of the population using public transport, obtained from the US Census Bureau.

A.8.18. *GINI INDEX WITHIN BOTTOM 99%.* The Gini coefficient minus 1% income share from Tax Records, core sample (Chetty et al., 2014).
Source: https://opportunityinsights.org/data/.

A.8.19. *DEMOCRATS VOTE SHARE IN 2016 GENERAL ELECTIONS.* The proportion of votes for Democrats in counties in the 2016 US presidential election obtained from MIT Election Data and Science Lab (2018).

A.8.20. *MOBILITY NEIGHBOR PROXY INDEX USING PRINCIPAL COMPONENT ANALYSIS.* The mobility proxies for neighboring counties are calculated as follows. Consider a county d. For each county d, we first use the US census to identify its neighboring counties. Then, for each neighboring county, we obtain five mobility proxy measures using SafeGraph Patterns data, which are the total number of residents from a county visiting POIs : 1) restaurants and other eating places, 2) clothing stores, 3) fitness and recreational centers, 4) movie theaters (except drive-ins), and 5) hobby centers and gaming stores. For each of the five mobility proxies, we take the average of all the neighboring counties. This yields five mobility proxies, each of which is averaged over neighboring counties; for example, the average total visitors of neighboring counties to clothing stores, where the average is taken over all of county d's neighboring counties. For county d, we have five neighboring mobility proxies that capture the average mobility behavior of neighboring counties in relation to the five categories of recreational locations. We apply PCA to the five average neighboring county mobility proxies to summarize the mobility behavior of neighboring county residents. We then retain the first four principal components that explain 99% of the variation in the mobility of population in neighboring counties.

We implement PCA using built-in Python class "PCA" from the Python library sci-kit learn. We use the function fit_transform within the PCA class. In the PCA, we first normalize the input variables and then calculate the singular value decomposition of the input. From that, we select the eigenvectors corresponding to the top four eigenvalues. Finally, we multiply these eigenvectors with the original data to obtain the four independent components.

In the analysis of the cumulative COVID-19 case and death prevalence and cumulative excess deaths, we use average measures of mobility proxies from March 1, 2020 to the end date of the period of the corresponding data sample. For the hospitalization and inpatient bed utilization outcomes, the mobility proxies are averages over the

period from July 1, 2020 to November 30, 2020. In the case of the monthly analysis, we use the monthly mean of the mobility proxies of each of the neighboring counties.

A.8.21. *GDP.* We collect county-level gross domestic product (GDP) data from the Bureau of Economic Analysis, US Department of Commerce. Under "Regional Economic Accounts: Download," we select the category "Gross Domestic Product (GDP)" and then download the file "CAGDP11: Contributions to Percent Change." We extract 2019 GDP data for three broad industry categories: private-services-providing industries, private-goods-producing industries, and non-government and government enterprises. In these data, (D) and (NA) are coded as missing data.

A.8.22. *GEOGRAPHIC VARIABLES.* We follow the data sources of Galor and Özak (2016) for the geographic variables used in our analysis. We use the GEcon 4.0 (May 2011) data on terrain roughness and elevation obtained from Nordhaus and Chen (2016), which are at a 1-degree level. We obtain the temperature and precipitation data from Climatic Research Unit (University of East Anglia) and Met Office; these data are at a 0.5-degree level. We obtain the boundaries of the different counties using the "bounds" function from Python's Shapely library. This gives us a start and end range of the latitude and longitude for each county.

For elevation and roughness, we find the point that falls within the county's latitude and longitude range and use the value of elevation and roughness at that point. For precipitation and temperature, we average over all the values that fall inside the boundaries of a particular county.

A.8.23. *COVID TESTING.* The data source for COVID testing is the CDC. We obtain the data from the US COVID Atlas, which is led by a coalition of research partners and contributors at the University of Chicago, accessed on July 9, 2021. We download county-level data on tests per 100,000, which denotes a "7-day rolling average of tests completed per 100,000 population in the county" as defined by the CDC. Initially, we sample the data between the start and end date. We keep only the dates that fall on the last day of the week (i.e., Sunday, which is day number six in Python). We then multiply this value by seven to obtain the total tests performed during that week. Then, for each county, we then take the sum of all the weekly totals to obtain the total tests per 100,000 population. To calculate the monthly tests, we take the sum of weekly totals for the relevant month.
Source link: https://theuscovidatlas.org/data.

A.8.24. *DISTANCE TO AIRPORT.* We follow Desmet and Wacziarg (2021) to compute the geodesic distance to the closest airport. We obtain the data on all incoming US international flights from Table T-100 from the US Department of Transportation's Bureau of Transportation Statistics. The data contain T-100 International Market (all carriers) and T-100 International Segment (all carriers). We include market and segment data given that both types of aircrafts can carry passengers. For each US airport, we take the mean number of monthly passengers on direct flights from the top countries in relation to COVID-19 cases on March 1, 2020 (i.e., China, Italy, Iran, South Korea, and Spain). We then keep the sample of airports for which the average monthly passengers arriving from these countries is at least 250. We then calculate the geodesic distance between each county and its nearest airport that receives at least 250 passengers from these countries. We also obtain longitude and latitude data on US airports from the Bureau of Transportation Statistics, using the file "Master Coordinate."

A.8.25. *HOSPITAL ATTRIBUTES.* For the year 2019, we obtain county-level data on the proportion of Catholic Church Operated Hospitals, the proportion of critical access hospitals, the proportion of rural referral centers, the proportion of Sole Community Providers, the proportion of hospitals with DNV Healthcare accreditation, the proportion of hospitals with Center for Improvement in Healthcare Quality accreditation, total full-time hospital unit personnel per hospital, total hospital bed per hospital, the proportion of short-term hospitals from American Hospital Association.

Further, we collect county-level data on the proportion of children's hospitals, proportion of critical access hospitals, and proportion of short-term hospitals in the facility-level hospitalization data that is available the period July 31, 2020 to November 30, 2020, published by the US Department of Health and Human Services, accessed September 27, 2021.

A.8.26. *PROPORTION OF FEMALES/MALES.* The five-year (2015–2019) average estimates of the proportion of the female or male population, obtained from the US Census Bureau.

A.9. **COVID CASES AND DEATHS.** We obtain the data on COVID-19 cases and deaths from E. Dong, H. Du, L. Gardner (2020), accessed on September 27, 2021. To obtain the cumulative cases and deaths from January 22, 2020 to November 30, 2020, we extract the column named 11/30/20 in the cases and the deaths file. To obtain the monthly cases/deaths, we calculate the data for each month by subtracting the cases/deaths on the last date of the month from the cases/deaths on the first date of the month.

Appendix B. Tables

Table B.1. DESCRIPTIVE STATISTICS

ANALYSIS SAMPLES:	Fitness Centers	Movie Theaters	Restaurants	Clothing Stores	Hobby Centers	Google Recreation
N	1802	825	1826	1754	1735	1038
**Mean Income**	68786.55	74432.9	68747	68879.84	68995.06	73082.24
	(13551.07)	(14922.14)	(13514.19)	(13627.76)	(13609.16)	(14286.42)
**Tot. Pop**	128179.4	250551.2	126559.5	131438.2	132769	210858.9
	(400148.6)	(567160.7)	(397756.8)	(405096.9)	(407108.4)	(511177.3)
**Work from Home (share)**	0.04790	0.04850	0.04830	0.04720	0.04720	0.0469
	(0.0252)	(0.0221)	(0.0261)	(0.0246)	(0.0246)	(0.0210)
**HH with ≥ 2 unit structure (share)**	0.5651	0.7195	0.5621	0.5714	0.5735	0.7198
	(0.3347)	(0.3726)	(0.3352)	(0.3369)	(0.3374)	(0.3368)
**HH with ≥ 3 members (share)**	0.5350	0.5615	0.5339	0.5366	0.5379	0.5565
	(0.0726)	(0.0708)	(0.0733)	(0.0721)	(0.0713)	(0.0679)
**Female (share)**	0.4999	0.5050	0.4998	0.5001	0.5002	0.5037
	(0.0214)	(0.0132)	(0.0215)	(0.0214)	(0.0211)	(0.0156)
**Black pop. (share)**	0.0945	0.0941	0.0934	0.0958	0.0956	0.0884
	(0.1438)	(0.1177)	(0.1432)	(0.1442)	(0.1423)	(0.1185)
**Indian American and Alaska Native pop. (share)**	0.0160	0.0120	0.0159	0.0145	0.0143	0.0134
	(0.0535)	(0.0380)	(0.0532)	(0.0487)	(0.0465)	(0.0428)
**White pop. (share)**	0.8277	0.8161	0.8290	0.8276	0.8276	0.8243
	(0.1560)	(0.1362)	(0.1556)	(0.1556)	(0.1537)	(0.1371)
**Hispanic or Latino* (share)**	0.0983	0.1094	0.0993	0.0999	0.0990	0.1118
	(0.1385)	(0.1249)	(0.1409)	(0.1403)	(0.1393)	(0.1401)
**Public Transport (share)**	0.0079	0.0118	0.0079	0.0079	0.0079	0.0114
	(0.0195)	(0.0255)	(0.0194)	(0.0195)	(0.0194)	(0.0250)
**H.S Graduate or Higher (share)**	0.8681	0.8808	0.8681	0.8672	0.8674	0.8799
	(0.0601)	(0.0503)	(0.0601)	(0.0602)	(0.0595)	(0.0529)
**Under 19 yrs (share)**	0.2489	0.2521	0.2486	0.2490	0.2494	0.2501
	(0.0338)	(0.0306)	(0.0341)	(0.0337)	(0.0334)	(0.0311)
**19-34 yrs (share)**	0.1817	0.1913	0.1813	0.1823	0.1826	0.1911
	(0.0346)	(0.0355)	(0.0348)	(0.0349)	(0.0347)	(0.0349)
**35-64 yrs (share)**	0.3835	0.3829	0.3834	0.3835	0.3838	0.3833
	(0.0267)	(0.0260)	(0.0267)	(0.0268)	(0.0264)	(0.0268)
**Above 65yrs and above (share)**	0.18590	0.17370	0.18660	0.18520	0.1842	0.1755
	(0.0434)	(0.0436)	(0.0440)	(0.0435)	(0.0421)	(0.0427)
**≥ 2 health insurance (under 19)(share)**	0.0623	0.0619	0.0622	0.0621	0.0619	0.0633
	(0.0261)	(0.0235)	(0.0262)	(0.0257)	(0.0253)	(0.0221)
**≥ 2 health insurance (19-34)(share)**	0.0604	0.0594	0.0604	0.0603	0.0603	0.0603
	(0.0253)	(0.0200)	(0.0256)	(0.0252)	(0.0245)	(0.0194)
**≥ 2 health insurance (35-64) (share)**	0.1013	0.0977	0.1014	0.1015	0.1012	0.0987
	(0.0302)	(0.0283)	(0.0309)	(0.0304)	(0.0301)	(0.0278)
**≥ 2 health insurance ($\geq$65) (share)**	0.6979	0.6954	0.6984	0.6977	0.6973	0.6995
	(0.0633)	(0.0602)	(0.0640)	(0.0632)	(0.0628)	(0.0597)
**% Obesity**	35.1338	33.7575	35.1527	35.1491	35.1082	33.8655
	(4.5059)	(4.4884)	(4.4937)	(4.5250)	(4.4870)	(4.5517)
**% Diabetes**	10.6321	10.2285	10.6084	10.6509	10.6654	10.3133
	(3.4543)	(2.8873)	(3.4434)	(3.4657)	(3.4343)	(2.8717)
**% Smokers**	21.2513	19.8560	21.2439	21.2527	21.2291	20.1864
	(4.0485)	(4.0006)	(4.0389)	(4.0437)	(4.0612)	(3.9128)
**Heart Disease Rate per 100k**	185.0323	177.0383	184.9233	185.6389	185.5191	177.1571
	(42.2790)	(39.2199)	(42.1817)	(42.5447)	(42.4669)	(40.8202)
**30-day Risk Adjusted Mortality Rate**	0.4608	0.3061	0.4586	0.4736	0.4620	0.3507
	(1.0606)	(0.8566)	(1.0642)	(1.0385)	(1.0411)	(0.8557)
**Perc. Ch. GDP by Services Industries**	2.2355	1.8990	2.2529	2.2147	2.1919	1.8669
	(2.6245)	(2.1921)	(2.6321)	(2.5632)	(2.5889)	(2.1301)
**Perc. Ch. GDP by Pvt. Goods Industries**	0.6694	0.3823	0.6928	0.6472	0.6275	0.4110
	(4.7748)	(3.1536)	(4.9188)	(4.7655)	(4.6303)	(2.7388)
**Perc. Ch. GDP by Govt. Enterprises**	0.0961	0.1402	0.0963	0.1005	0.0933	0.1328
	(0.5821)	(0.3990)	(0.5836)	(0.5857)	(0.5673)	(0.4537)
**Perc. Ch. in Annual Avg. Empl.**	0.6188	0.9725	0.6323	0.6526	0.6323	0.9710
	(3.0497)	(2.1569)	(3.1403)	(3.1594)	(3.0766)	(2.0327)
**Gini Index**	0.3847	0.4038	0.3840	0.3865	0.3863	0.4021
	(0.0841)	(0.0870)	(0.0844)	(0.0839)	(0.0840)	(0.0823)
**Pop. below Poverty Line (share)**	0.1416	0.1240	0.1418	0.1415	0.1408	0.1270
	(0.0611)	(0.0490)	(0.0611)	(0.0612)	(0.0602)	(0.0537)
**Precipitation (mm/month)**	88.8802	91.5975	88.4532	89.7252	89.9326	91.3583
	(32.4263)	(31.0863)	(32.5071)	(31.9313)	(32.0011)	(31.7494)
**Temperature (celsius)**	13.8491	14.3190	13.8188	13.9880	13.9642	13.6978
	(4.8097)	(4.5723)	(4.8199)	(4.7777)	(4.7555)	(4.8894)
**Elevation (metres)**	458.6612	453.9925	460.8637	448.0047	446.5282	468.9879
	(515.4201)	(532.2192)	(514.3082)	(507.0406)	(508.9881)	(551.2869)

Notes: The table presents summary statistics for our sample analyzed in Table 4, Table 5, and Appendix Table B.14.

Table B.2. EFFECT OF LONG-TERM ORIENTATION ON COVID-19 SEVERITY: ACCOUNTING FOR THE SHARE OF NURSING HOME RESIDENTS

	JAN-NOVEMBER 2020	
	**(1)**	**(2)**
	COVID-19 cases	COVID-19 deaths
Pre-1500 CE Yield (Anc.)	-6.783	-6.593
	[4.736]	[5.321]
Post-1500 CE Yield Ch (Anc.)	-1.044***	-0.975***
	[0.191]	[0.225]
Observations	1,949	1,949
R-squared	0.835	0.821
State F.E	Yes	Yes
Neighboring county COVID-19 prev and mobility	Yes	Yes
All Geographic Controls	Yes	Yes
All Socio-Economic Controls	Yes	Yes
Avg. Nursing Home Residents/day	Yes	Yes

Notes: The table reports the effects of the ancestry-adjusted pre-1500 CE potential crop yield and the ancestry-adjusted post-1500 crop yield change due to the Columbian Exchange on county-level COVID-19 case prevalence in column (1) and on county-level COVID-19 death prevalence in column (2), based on OLS estimates of Equation 1. COVID-19 case prevalence in a county is measured as the total county-level COVID-19 confirmed cases per test per 100,000. The prevalence of deaths from COVID-19 in a county is measured as total county-level COVID-19 deaths per test per 100,000. All variables are normalized by subtracting their mean and dividing by their standard deviation. Therefore, all coefficients are comparable and estimate the effect of a one standard deviation increase in the independent variable. All columns have state fixed effects; share of nursing home residents; geographic factors that include average roughness of terrain, precipitation, temperature, and elevation; county-level COVID-19 prevalence in neighboring counties, which is measured as the confirmed COVID-19 cases per test per 100,000 averaged over all neighboring counties, mobility proxies in neighboring counties; dummy variables for the urban status of each county comprising large central or large fringe metro counties and medium metro and small metro counties; mean income; proportion of males; population density; proportion of population from a Black or African American, Native American, White, and Hispanic or Latino background; proportion of population using public transport; proportion of family and non-family households living in two or more unit structures; proportion of family and non-family households with three or more members; proportion of population in age groups younger than 19, 19–34, and 35–64 (with 65 and older as the omitted group); proportion working from home; proportion of population with an education level higher than or equal to the higher secondary level; healthcare coverage measured as the proportion of the population with two or more health insurance policies in the age groups younger than 19, 19–34, 35–64, and 65 and older, and the proportion of the population older than age 65 without any health insurance; distance to an airport with direct international flights to high-severity countries; Gini index and proportion of population below the poverty line; Social Capital Index; percentage of adult population with obesity; percentage of population who smoke as adults; 30-day risk-adjusted mortality rate; heart disease death rate; percentage of population diagnosed with diabetes among adults older than 20 years of age; contribution to the percentage change in GDP by private-services-providing industries, private-goods-providing industries, and government enterprises, and government enterprises; percentage change in annual average employment for a given year; and proportion of votes for Democrats in counties in the 2016 US presidential election. Predicted ancestral compositions in each county using the instruments from Burchardi, Chaney and Hassan (2019) and the post-1500 World Migration Matrix of Putterman and Weil (2010) were used to adjust crop yield measures. Bootstrap standard errors account for clustering at the commuting zone level using the bootstrap command with the cluster option in STATA.

Table B.3. DYNAMIC CAUSAL EFFECT OF LONG-TERM ORIENTATION ON COVID-19 SEVERITY

	APRIL-NOVEMBER 2020							
	(1)	(2)	(3)	(4)	(5)	(6)	(7)	(8)
Per Test per 100k	Cases	Cases	Deaths	Deaths	Cases	Cases	Deaths	Deaths
High Pre-1500 CE Yield (Anc.)*May	-0.337***	-0.366***	-0.210*	-0.195**				
	(0.0010)	(0.0010)	(0.0621)	(0.0370)				
High Pre-1500 CE Yield (Anc.)*Jun	-0.313***	-0.374***	-0.199*	-0.200**				
	(0.0060)	(0.0030)	(0.0601)	(0.0350)				
High Pre-1500 CE Yield (Anc.)*Jul	-0.299***	-0.399**	-0.187*	-0.198**				
	(0.0060)	(0.0120)	(0.0591)	(0.0460)				
High Pre-1500 CE Yield (Anc.)*Aug	-0.301***	-0.455***	-0.192**	-0.226**				
	(0.0040)	(0.0090)	(0.0480)	(0.0380)				
High Pre-1500 CE Yield (Anc.)*Sep	-0.314***	-0.478**	-0.201**	-0.213*				
	(0.0020)	(0.0110)	(0.0440)	(0.0611)				
High Pre-1500 CE Yield (Anc.)*Oct	-0.313***	-0.490**	-0.204**	-0.210*				
	(0.0040)	(0.0120)	(0.0380)	(0.0731)				
High Pre-1500CE Yield (Anc.)*Nov	-0.302***	-0.511**	-0.193*	-0.197				
	(0.0040)	(0.0290)	(0.0631)	(0.1401)				
High Post-1500 CE Yield Ch. (Anc.)*May					-0.201*	-0.177	-0.248**	-0.225**
					(0.0731)	(0.1071)	(0.0230)	(0.0110)
High Post-1500 CE Yield Ch. (Anc.)*Jun					-0.171	-0.159	-0.217**	-0.208**
					(0.1351)	(0.1421)	(0.0310)	(0.0110)
High Post-1500 CE Yield Ch. (Anc.)*Jul					-0.153	-0.147	-0.200**	-0.194***
					(0.1391)	(0.1401)	(0.0260)	(0.0100)
High Post-1500 CE Yield Ch. (Anc.)*Aug					-0.159	-0.182*	-0.205**	-0.217***
					(0.1371)	(0.0661)	(0.0270)	(0.0070)
High Post-1500 CE Yield Ch. (Anc.)*Sep					-0.175*	-0.175**	-0.219**	-0.194***
					(0.0971)	(0.0420)	(0.0220)	(0.0090)
High Post-1500 CE Yield Ch. (Anc.)*Oct					-0.171	-0.152*	-0.226**	-0.181***
					(0.1161)	(0.0611)	(0.0130)	(0.0030)
High Post-1500 CE Yield Ch. (Anc.)*Nov					-0.166	-0.138*	-0.231***	-0.165***
					(0.1522)	(0.0881)	(0.0090)	(0.0050)
Observations	17,945	17,945	17,945	17,945	17,945	17,945	17,945	17,945
R-squared	0.125	0.190	0.234	0.320	0.123	0.188	0.234	0.320
Number of counties	2,371	2,371	2,371	2,371	2,371	2,371	2,371	2,371
State*Trends	No	Yes	No	Yes	No	Yes	No	Yes
Geographic Controls	Yes	Yes	Yes	Yes	Yes	Yes	Yes	Yes
Mobility Neighboring County	Yes	Yes	Yes	Yes	Yes	Yes	Yes	Yes

Notes:"()" reports the p-values of the wild cluster bootstrap method using the post-estimation command boottest, clustered at the commuting zone level. Stars $^{***(**)[*]}$ indicate significance at the $0.01(0.05)[0.1]$ level. This table shows the effect of the ancestry-adjusted high pre-1500 CE potential crop yield (where ancestry-adjusted high pre-1500 CE potential crop yield is a dummy variable that equals 1 if the ancestry-adjusted potential crop yield is above the median value of the regression sample) and the ancestry-adjusted high post-1500 potential crop yield change during the Columbian Exchange (where ancestry-adjusted high potential yield change is a dummy variable that equals 1 if the potential crop yield change is above the median value of the regression sample) on COVID-19 case prevalence and COVID-19 death prevalence at the county level for every month from April 1, 2020 to November 30, 2020. The reported effects are relative to the baseline COVID-19 case prevalence and death prevalence in the month of April 2020, based on Equation 2. COVID-19 monthly case prevalence is measured as monthly total COVID-19 cases per test per 100,000. COVID-19 monthly death prevalence is measured as monthly total COVID-19 deaths per test per 100,000. State trends are added as controls in columns (2), (4), (6), and (8). All columns control for the average COVID-19 case prevalence in neighboring counties, monthly mobility in neighboring counties, and geographic factors consisting of temperature and precipitation. Predicted ancestral compositions in each county using the instruments from Burchardi, Chaney and Hassan (2019) and the post-1500 World Migration Matrix of Putterman and Weil (2010) were used to adjust crop yield measures. All variables are normalized by subtracting their mean and dividing by their standard deviation. Therefore, all coefficients are comparable and estimate the effect of a one standard deviation increase in the independent variable.

Table B.4. CAUSAL EFFECT OF LONG-TERM ORIENTATION ON HOSPITAL USE

JULY 31, 2020 to NOVEMBER 30, 2020				
**PANEL A: TOTAL HOSPITALIZATION WITH COVID-19 PER TESTS PER 100k**	**(1)**	**(2)**	**(3)**	**(4)**
Pre-1500 CE Yield (Anc.)	-6.727<br>[6.882]	-9.636<br>[7.193]	-10.07<br>[6.651]	-6.596<br>[5.694]<br>$\langle p = 0.2402 \rangle$
Post-1500 Yield Ch. (Anc.)	-1.143<br>[0.245]***	-1.042<br>[0.239]***	-0.993<br>[0.228]***	-0.960<br>[0.236]***<br>$\langle p = 0.0050 \rangle$
Pre-1500 Cycle (Anc.)	4.554<br>[7.045]	7.069<br>[7.194]	7.306<br>[6.657]	4.006<br>[5.652]
Post-1500 Cycle Ch. (Anc.)	3.049***<br>[0.572]	3.290***<br>[0.621]	3.396***<br>[0.563]	3.105***<br>[0.559]
Observations	1,693	1,693	1,693	1,693
R-squared	0.681	0.696	0.722	0.796
**PANEL B: AVG WEEKLY TOTAL INPATIENT BED USED PER HOSPITAL**	**(1)**	**(2)**	**(3)**	**(4)**
Pre-1500 CE Yield (Anc.)	-10.78<br>[6.596]	-15.00<br>[6.560]**	-12.81<br>[6.349]**	-3.443<br>[1.540]**<br>$\langle p = 0.0110 \rangle$
Post-1500 Yield Ch. (Anc.)	-0.305<br>[0.226]	-0.156<br>[0.228]	-0.117<br>[0.235]	0.00005<br>[0.0769]<br>$\langle p = 1.00 \rangle$
Pre-1500 Cycle (Anc.)	11.16<br>[6.775]*	14.89<br>[6.565]**	12.74<br>[6.368]**	3.215<br>[1.552]**
Post-1500 Cycle Ch. (Anc.)	0.307<br>[0.586]	0.600<br>[0.635]	0.487<br>[0.638]	0.196<br>[0.203]
Observations	1,702	1,702	1,702	1,702
R-squared	0.254	0.277	0.288	0.818
Geographic	N	Y	Y	Y
Neighbor Mob.& COVID-19 prev.	N	N	Y	Y
Socio-Economic, Pol., Health	N	N	N	Y
State Fixed Effects	Y	Y	Y	Y

Standard errors are denoted as follows: [bootstrap standard errors account for clustering at the commuting zone level using the bootstrap command with the cluster option in STATA; that is, in this method, the sample drawn during each replication is a bootstrap sample of commuting zones] and <*p*-values are generated by the wild cluster bootstrap method using the boottest command written by Roodman et al. (2019), clustered at the commuting zone level>. Stars $^{***(**)[*]}$ indicate significance at the $0.01(0.05)[0.1]$ level. This table shows the effect of the ancestry-adjusted pre-1500 CE potential crop yield and post-1500 crop yield change due to the Columbian Exchange on county-level hospital use during the period July 31 to November 30. The outcome variable in panel A is the sum of reports of patients hospitalized in an adult inpatient bed who have laboratory-confirmed COVID-19 per test per 100,000. The outcome variable in panel B is the average weekly sum of reports of total staffed inpatient beds that are reported to be occupied per hospital in each county. The sample includes all hospitals registered with CMS as of June 1, 2020 and includes non–CMS hospitals that have reported since July 1, 2020. The sample does not include psychiatric facilities, rehabilitation facilities, Indian health service facilities, US Department of Veterans Affairs facilities, Defense health agency facilities, and religious non-medical facilities. All columns have state fixed effects. All variables are normalized by subtracting their mean and dividing by their standard deviation. Therefore, all coefficients are comparable and estimate the effect of a one standard deviation increase in the independent variable. Column (1) includes only ancestry-adjusted pre-1500 CE crop yields, crop growth cycles, and their changes. Column (2) adds geographic controls such as mean roughness of terrain, mean precipitation, average temperature, and average elevation. Column (3) adds controls for county-level COVID-19 prevalence in neighboring counties, which is measured as the confirmed COVID-19 cases per test per 100,000 averaged over all neighboring counties. Further, for each county, we generate controls for mobility in neighboring counties by applying PCA on five mobility proxies that measure the average number of total visitors from neighboring counties to the following five POIs: hobby centers, restaurants, clothing stores, fitness centers, and movie theaters (except drive-ins). The first four components (PCA) that explain 99% of variation are added in column (3). Column (4) adds dummy variables for the urban status of each county (composed of large central metro, large fringe metro, medium metro, or small metro county, as defined by the National Center for Health Statistics); mean income; proportion of males; population density; proportion of population from a Black or African American, Native American, White, and Hispanic or Latino background; proportion of population working from home; proportion of population using public transport; proportion of family and non-family households living in two or more unit structures; proportion of family and non-family households with three or more members; proportion of population in age groups younger than 19, 19–34, and 35–64 (with 65 and older as the omitted group); proportion of population with an education level higher than or equal to higher secondary level; healthcare coverage measured as the proportion of the population with two or more health insurance policies in the age groups younger than 19, 19–34, 35–64, and 65 and older; and proportion of population older than age 65 without any health insurance. Column (4) further adds distance to an airport with direct international flights to high-severity countries measured following the method in Desmet and Wacziarg (2021); Gini index and proportion of population below the poverty line; Social Capital Index; percentage of adult population with obesity; percentage of population who smoke as adults; 30-day risk-adjusted mortality rate; heart disease death rate; percentage of population diagnosed with diabetes among adults older than 20 years of age; contribution to the percentage change in real GDP by private-services-providing industries, private-goods-providing industries, and government enterprises, and government enterprises; percentage change in the annual average employment for a given year; and the proportion of votes for Democrats in counties in the 2016 US presidential election as a proxy for pre-crisis political orientation. Additionally, column (4) adds controls measuring pre-crisis county-level hospital capacity (includes proportion of full-time staff, beds available, hospitals that were short term, hospitals operated by the Catholic Church, critical access hospitals, rural referral centers, and sole community providers; proportion with DNV Healthcare accreditation; and proportion of hospitals with a Center for Improvement in Healthcare Quality accreditation) and types of hospital reporting (comprising the proportion of critical access hospitals reporting, children's hospitals reporting, and short-term hospitals). In Panel B, column (4) also adds county-level COVID-19 tests per 100,000. Predicted ancestral compositions in each county using the instruments from Burchardi, Chaney and Hassan (2019) and post-1500 World Migration Matrix of Putterman and Weil (2010) were used to adjust crop yield measures.

Table B.5. OMITTED VARIABLE BIAS TESTS

	(1) COVID-19 Cases	(2) COVID-19 Deaths	(3) Excess (>=45)	(4) Excess (>=55)	(5) Excess (>=65)
Pre-1500 CE Yield (Anc.)	-7.135	-6.731	-0.451	-0.886	-0.223
	[4.686]	[5.249]	[4.416]	[4.996]	[4.965]
Post-1500 CE Yield Ch. (Anc.)	-1.042***	-0.981***	-0.812***	-0.858***	-0.863***
	[0.192]	[0.221]	[0.216]	[0.256]	[0.279]
Pre-1500 CE Cycle (Anc.)	5.607	6.243	0.772	1.194	0.653
	[4.738]	[5.466]	[4.669]	[5.264]	[5.111]
Post-1500 CE Cycle Ch. (Anc.)	2.551***	1.926***	1.281**	1.397**	1.337**
	[0.454]	[0.512]	[0.613]	[0.659]	[0.690]
Geographic	Y	Y	Y	Y	Y
Socio-Economic, Pol., Health	Y	Y	Y	Y	Y
State Fixed Effects	Y	Y	Y	Y	Y
**(CHANGE IN YIELD)**					
β	-5.29	-5.38	-2.91	-3.64	-4.01
Observations	2,034	2,036	897	1,482	1,753
R-squared	0.825	0.816	0.903	0.883	0.872

Notes: Standard errors are denoted as follows: [bootstrap standard errors account for clustering at the commuting zone level using the bootstrap command with the cluster option in STATA; that is, in this method, the sample drawn during each replication is a bootstrap sample of commuting zones]. Stars $^{***(**)[*]}$ indicate significance at the $0.01(0.05)[0.1]$ level. This table reports the effects of the ancestry-adjusted pre-1500 CE crop yield and the ancestry-adjusted post-1500 crop yield change on county-level COVID-19 case prevalence, death prevalence, and excess deaths in age groups 45 and older, 55 and older, and 65 and older. All columns include state fixed effects; geographic factors that include average roughness of terrain, precipitation, temperature, and elevation; COVID-19 prevalence in neighboring counties; mobility proxies in neighboring counties; dummy variables for the urban status of each county comprising large central or large fringe metro counties and medium metro and small metro counties; mean income; proportion of males; population density; proportion of population from a Black or African American, Native American, White, and Hispanic or Latino background; proportion of population using public transport; proportion of family and non-family households living in two or more unit structures; proportion of family and non-family households with three or more members; proportion of population in age groups younger than 19, 19–34, and 35–64 (with 65 and older as the omitted group); proportion working from home; proportion of population with an education level higher than or equal to the higher secondary level; healthcare coverage measured as the proportion of the population with two or more health insurance policies in the age groups younger than 19, 19–34, 35–64, and 65 and older, and the proportion of the population older than age 65 without any health insurance; distance to an airport with direct international flights to high-severity countries; Gini index and proportion of population below the poverty line; Social Capital Index; percentage of adult population with obesity; percentage of population who smoke as adults; 30-day risk-adjusted mortality rate; heart disease death rate; percentage of population diagnosed with diabetes among adults older than 20 years of age; contribution to the percentage change in GDP by private-services-providing industries, private-goods-providing industries, and government enterprises, and government enterprises; percentage change in annual average employment for a given year; and proportion of votes for Democrats in counties in the 2016 US presidential election. Columns (3)-(5) also controls for county-level COVID-19 tests per 100,000. The dependent variables in columns (1) and (2) account for COVID-19 testing. All variables are normalized by subtracting their mean and dividing by their standard deviation. Therefore, all coefficients are comparable and estimate the effect of a one standard deviation increase in the independent variable. $\beta(1,1)$ statistics suggested by Oster (2019) are presented.

Table B.6. CAUSAL EFFECT OF MEASURES OF CROP YIELDS ON COVID-19 SEVERITY

	JANUARY 22, 2020 to NOVEMBER 30, 2020							
Per test per 100K	Cases	Cases	Cases	Cases	Deaths	Deaths	Deaths	Deaths
	**(1)**	**(2)**	**(3)**	**(4)**	**(5)**	**(6)**	**(7)**	**(8)**
Post-1500 CE Yield	-0.0852			-1.516	-0.106			-2.191
	[0.0865]			[5.351]	[0.0908]			[5.745]
				$\langle p = 0.741\rangle$				$\langle p = 0.649\rangle$
Post-1500 CE Cycle	0.0138			0.0802	0.0399			0.0551
	[0.0549]			[0.230]	[0.0609]			[0.243]
Pre-1500 CE Yield		-0.0799	-0.0592	1.774		-0.0940	-0.0593	2.508
		[0.0928]	[0.0794]	[6.023]		[0.0990]	[0.0812]	[6.473]
				$\langle p = 0.756\rangle$				$\langle p = 0.681\rangle$
Pre-1500 CE Cycle			-0.0133	-0.0622			-0.00772	-0.0280
			[0.0683]	[0.210]			[0.0813]	[0.224]
Post-1500 CE Yield Ch.		0.0368	0.0424	0.285		0.0324	0.0468	0.415
		[0.0379]	[0.0412]	[0.988]		[0.0439]	[0.0489]	[1.060]
				$\langle p = 0.755\rangle$				$\langle p = 0.690\rangle$
Post-1500 CE Cycle Ch.			0.0240	-0.0311			0.0540	-0.0022
			[0.0344]	[0.0995]			[0.0366]	[0.106]
Pre-1500 CE Yield (Anc.)				-7.729				-6.007
				[6.136]				[6.758]
				$\langle p = 0.279\rangle$				$\langle p = 0.513\rangle$
Post-1500 CE Yield Ch. (Anc.)				-1.096				-0.977
				[0.201]***				[0.235]***
				$\langle p = 0.000\rangle$				$\langle p = 0.000\rangle$
Pre-1500 CE Cycle (Anc.)				6.267				5.350
				[6.357]				[7.119]
Post-1500 CE Cycle Ch. (Anc.)				2.521				2.018
				[0.491]***				[0.554]***
Observations	2,026	2,026	2,026	2,026	2,026	2,026	2,026	2,026
R-squared	0.544	0.544	0.545	0.826	0.504	0.504	0.506	0.818
Geographic	Y	Y	Y	Y	Y	Y	Y	Y
Neighbor Mob.& COVID-19 prev.	Y	Y	Y	Y	Y	Y	Y	Y
Socio-Economic, Pol., Health	Y	Y	Y	Y	Y	Y	Y	Y
State Fixed Effects	Y	Y	Y	Y	Y	Y	Y	Y

Notes: Standard errors are denoted as follows: [bootstrap standard errors account for clustering at the commuting zone level using the bootstrap command with the cluster option in STATA; that is, in this method, the sample drawn during each replication is a bootstrap sample of commuting zones]. <*p*-values are generated by the wild cluster bootstrap method using the boottest command written by Roodman et al. (2019), clustered at the commuting zone level>. Stars $^{***(**)[*]}$ indicate significance at the $0.01(0.05)[0.1]$ level. This table presents the effect of a county's unadjusted and ancestry-adjusted pre-1500 CE potential crop yield, post-1500 CE potential crop yield and its change due to the Columbian Exchange, and ancestry-adjusted post-1500 crop yield change on its COVID-19 cases and deaths per test per 100,000. All columns include state fixed effects; geographic factors that include average roughness of terrain, precipitation, temperature, and elevation; COVID-19 prevalence in neighboring counties; mobility proxies in neighboring counties; dummy variables for the urban status of each county comprising large central or large fringe metro counties and medium metro and small metro counties; mean income; proportion of males; population density; proportion of population from a Black or African American, Native American, White, and Hispanic or Latino background; proportion of population using public transport; proportion of family and non-family households living in two or more unit structures; proportion of family and non-family households with three or more members; proportion of population in age groups younger than 19, 19–34, and 35–64 (with 65 and older as the omitted group); proportion working from home; proportion of population with an education level higher than or equal to the higher secondary level; healthcare coverage measured as the proportion of the population with two or more health insurance policies in the age groups younger than 19, 19–34, 35–64, and 65 and older, and the proportion of the population older than age 65 without any health insurance; distance to an airport with direct international flights to high-severity countries; Gini index and proportion of population below the poverty line; Social Capital Index; percentage of adult population with obesity; percentage of population who smoke as adults; 30-day risk-adjusted mortality rate; heart disease death rate; percentage of population diagnosed with diabetes among adults older than 20 years of age; contribution to the percentage change in GDP by private-services-providing industries, private-goods-providing industries, and government enterprises, and government enterprises; percentage change in annual average employment for a given year; and proportion of votes for Democrats in counties in the 2016 US presidential election. All variables are normalized by subtracting their mean and dividing by their standard deviation. Therefore, all coefficients are comparable and estimate the effect of a one standard deviation increase in the independent variable.

Table B.7. CAUSAL EFFECT OF MEASURES OF CROP YIELDS ON EXCESS DEATHS (AGE 45 AND OLDER)

	JANUARY 1, 2020 to DECEMBER 31, 2020			
**PANEL A: 45 years and above**	**(1)**	**(2)**	**(3)**	**(4)**
	Excess Deaths	Excess Deaths	Excess Deaths	Excess Deaths
Post-1500 CE Yield	-0.129<br>[0.116]			2.664<br>[5.523]<br>$\langle p = 0.429 \rangle$
Post-1500 CE Cycle	0.153<br>[0.102]			-0.138<br>[0.394]
Pre-1500 CE Yield		-0.0788<br>[0.138]	-0.0813<br>[0.128]	-3.082<br>[6.268]<br>$\langle p = 0.434 \rangle$
Pre-1500 CE Cycle			0.107<br>[0.114]	0.234<br>[0.388]
Post-1500 CE Yield Ch.		0.195<br>[0.182]	0.208<br>[0.187]	-0.504<br>[1.040]<br>$\langle p = 0.429 \rangle$
Post-1500 CE Cycle Ch.			0.0923<br>[0.0753]	0.0989<br>[0.187]
Pre-1500 CE Yield (Anc.)				-1.771<br>[5.283]<br>$\langle p = 0.798 \rangle$
Post-1500 CE Yield Ch. (Anc.)				-0.807<br>[0.238]***<br>$\langle p = 0.0030 \rangle$
Pre-1500 CE Cycle (Anc.)				2.191<br>[5.780]
Post-1500 CE Cycle Ch. (Anc.)				1.215<br>[0.675]*
Observations	894	894	894	894
R-squared	0.537	0.538	0.541	0.903
Geographic	Y	Y	Y	Y
Neighbor Mob.& COVID-19 prev.	Y	Y	Y	Y
Socio-Economic, Pol., Health	Y	Y	Y	Y
State Fixed Effects	Y	Y	Y	Y

Notes: Standard errors are denoted as follows: [bootstrap standard errors account for clustering at the commuting zone level using the bootstrap command with the cluster option in STATA (i.e., in this method, the sample drawn during each replication is a bootstrap sample of commuting zones)]. Stars $^{***(**)[*]}$ indicate significance at the $0.01(0.05)[0.1]$ level. <*p*-values are generated by the wild cluster bootstrap method using the boottest command written by Roodman et al. (2019), clustered at the commuting zone level>. This table presents the effect of a county's unadjusted and ancestry-adjusted pre-1500 CE potential crop yield, post-1500 CE potential crop yield, and ancestry-adjusted and unadjusted post-1500 crop yield change due to the Columbian Exchange on its excess deaths. Excess deaths for the age group "x" and older is measured as total deaths in 2020 in the age group "x" and older minus the expected deaths in 2020 in the age group "x" and older, where the expected deaths is measured by multiplying total population in the age group "x" and older in 2019 with the death rate in the age group "x" and older in 2018. All columns include state fixed effects; geographic factors that include average roughness of terrain, precipitation, temperature, and elevation; COVID-19 prevalence in neighboring counties; mobility proxies in neighboring counties; dummy variables for the urban status of each county comprising large central or large fringe metro counties and medium metro and small metro counties; mean income; proportion of males; county-level COVID-19 tests per 100,000; population density; proportion of population from a Black or African American, Native American, White, and Hispanic or Latino background; proportion of population using public transport; proportion of family and non-family households living in two or more unit structures; proportion of family and non-family households with three or more members; proportion of population in age groups younger than 19, 19–34, and 35–64 (with 65 and older as the omitted group); proportion working from home; proportion of population with an education level higher than or equal to the higher secondary level; healthcare coverage measured as the proportion of the population with two or more health insurance policies in the age groups younger than 19, 19–34, 35–64, and 65 and older, and the proportion of the population older than age 65 without any health insurance; distance to an airport with direct international flights to high-severity countries; Gini index and proportion of population below the poverty line; Social Capital Index; percentage of adult population with obesity; percentage of population who smoke as adults; 30-day risk-adjusted mortality rate; heart disease death rate; percentage of population diagnosed with diabetes among adults older than 20 years of age; contribution to the percentage change in GDP by private-services-providing industries, private-goods-providing industries, and government enterprises, and government enterprises; percentage change in annual average employment for a given year; and proportion of votes for Democrats in counties in the 2016 US presidential election. All variables are normalized by subtracting their mean and dividing by their standard deviation. Therefore, all coefficients are comparable and estimate the effect of a one standard deviation increase in the independent variable.

Table B.8. CAUSAL EFFECT OF MEASURES OF CROP YIELDS ON EXCESS DEATHS (AGE 55 AND OLDER)

	JANUARY 1, 2020 to DECEMBER 31, 2020			
**PANEL A: 55 years and above**	**(1)**	**(2)**	**(3)**	**(4)**
	Excess Deaths	Excess Deaths	Excess Deaths	Excess Deaths
Post-1500 CE Yield	-0.135<br>[0.0916]			1.576<br>[3.719]<br>$\langle p = 0.614 \rangle$
Post-1500 CE Cycle	0.132<br>[0.0875]			0.0227<br>[0.172]
Pre-1500 CE Yield		-0.107<br>[0.103]	-0.0866<br>[0.0833]	-1.830<br>[4.235]<br>$\langle p = 0.643 \rangle$
Pre-1500 CE Cycle			0.0538<br>[0.103]	0.0780<br>[0.168]
Post-1500 CE Yield Ch.		0.0538<br>[0.0781]	0.0876<br>[0.0949]	-0.320<br>[0.715]<br>$\langle p = 0.618 \rangle$
Post-1500 CE Cycle Ch.			0.0880<br>[0.0630]	0.0245<br>[0.0771]
Pre-1500 CE Yield (Anc.)				-2.145<br>[5.902]<br>$\langle p = 0.799 \rangle$
Post-1500 CE Yield Ch. (Anc.)				-0.879<br>[0.283]***<br>$\langle p = 0.030 \rangle$
Pre-1500 CE Cycle (Anc.)				2.572<br>[6.420]
Post-1500 CE Cycle Ch. (Anc.)				1.329<br>[0.709]*
Observations	1,478	1,478	1,478	1,478
R-squared	0.504	0.502	0.506	0.884
Geographic	Y	Y	Y	Y
Neighbor Mob.& COVID-19 prev.	Y	Y	Y	Y
Socio-Economic, Pol., Health	Y	Y	Y	Y
State Fixed Effects	Y	Y	Y	Y

Standard errors are denoted as follows: [bootstrap standard errors account for clustering at the commuting zone level using the bootstrap command with the cluster option in STATA; that is, in this method, the sample drawn during each replication is a bootstrap sample of commuting zones]. Stars $^{***(**)[*]}$ indicate significance at the $0.01(0.05)[0.1]$ level. <*p*-values are generated by the wild cluster bootstrap method using the boottest command written by Roodman et al. (2019), clustered at the commuting zone level>. This table presents the effect of a county's unadjusted and ancestry-adjusted pre-1500 CE potential crop yield, post-1500 CE potential crop yield, and ancestry-adjusted and unadjusted post-1500 crop yield change due to the Columbian Exchange on its excess deaths. Excess deaths for the age group "x" and older is measured as total deaths in 2020 in the age group "x" and older minus the expected deaths in 2020 in the age group "x" and older, where the expected deaths is measured by multiplying total population in the age group "x" and older in 2019 with the death rate in the age group "x" and older in 2018. All columns include state fixed effects; geographic factors that include average roughness of terrain, precipitation, temperature, and elevation; COVID-19 prevalence in neighboring counties; mobility proxies in neighboring counties; dummy variables for the urban status of each county comprising large central or large fringe metro counties and medium metro and small metro counties; mean income; proportion of males; county-level COVID-19 tests per 100,000; population density; proportion of population from a Black or African American, Native American, White, and Hispanic or Latino background; proportion of population using public transport; proportion of family and non-family households living in two or more unit structures; proportion of family and non-family households with three or more members; proportion of population in age groups younger than 19, 19–34, and 35–64 (with 65 and older as the omitted group); proportion working from home; proportion of population with an education level higher than or equal to the higher secondary level; healthcare coverage measured as the proportion of the population with two or more health insurance policies in the age groups younger than 19, 19–34, 35–64, and 65 and older, and the proportion of the population older than age 65 without any health insurance; distance to an airport with direct international flights to high-severity countries; Gini index and proportion of population below the poverty line; Social Capital Index; percentage of adult population with obesity; percentage of population who smoke as adults; 30-day risk-adjusted mortality rate; heart disease death rate; percentage of population diagnosed with diabetes among adults older than 20 years of age; contribution to the percentage change in GDP by private-services-providing industries, private-goods-providing industries, and government enterprises, and government enterprises; percentage change in annual average employment for a given year; and proportion of votes for Democrats in counties in the 2016 US presidential election. All variables are normalized by subtracting their mean and dividing by their standard deviation. Therefore, all coefficients are comparable and estimate the effect of a one standard deviation increase in the independent variable.

Table B.9. CAUSAL EFFECT OF MEASURES OF CROP YIELDS ON EXCESS DEATHS (AGE 65 AND OLDER)

	1 JANUARY 2020 - 31 DECEMBER 2020			
	Excess Deaths	Excess Deaths	Excess Deaths	Excess Deaths
**PANEL C: 65 years and above**	**(1)**	**(2)**	**(3)**	**(4)**
Post-1500 CE Yield	-0.134<br>[0.0837]			2.168<br>[3.844]<br>$\langle p = 0.624 \rangle$
Post-1500 CE Cycle	0.120<br>[0.0758]			0.0366<br>[0.173]
Pre-1500 CE Yield		-0.105<br>[0.0944]	-0.0960<br>[0.0731]	-2.508<br>[4.386]<br>$\langle p = 0.586 \rangle$
Pre-1500 CE Cycle			0.0499<br>(0.0894)	0.0653<br>(0.167)
Post-1500 CE Yield Ch.		0.0282<br>[0.0608]	0.0556<br>[0.0724]	-0.432<br>[0.747]<br>$\langle p = 0.553 \rangle$
Post-1500 CE Cycle Ch.			0.0760<br>(0.0546)	0.0217<br>(0.0758)
Pre-1500 CE Yield (Anc.)				-1.450<br>[5.919]<br>$\langle p = 0.864 \rangle$
Post-1500 CE Yield Ch. (Anc.)				-0.889<br>[0.308]***<br>$\langle p = 0.0270 \rangle$
Pre-1500 CE Cycle (Anc.)				2.015<br>[6.338]
Post-1500 CE Cycle Ch. (Anc.)				1.261<br>[0.737]*
Observations	1,749	1,749	1,749	1,749
R-squared	0.495	0.493	0.496	0.873
Geographic	Y	Y	Y	Y
Neighbor Mob.& COVID-19 prev.	Y	Y	Y	Y
Socio-Economic, Pol., Health	Y	Y	Y	Y
State Fixed Effects	Y	Y	Y	Y

Standard errors are denoted as follows: [bootstrap standard errors account for clustering at the commuting zone level using the bootstrap command with the cluster option in STATA; that is, in this method, the sample drawn during each replication is a bootstrap sample of commuting zones]. Stars ***(**)[*] indicate significance at the 0.01(0.05)[0.1] level. <*p*-values are generated by the wild cluster bootstrap method using the boottest command written by Roodman et al. (2019), clustered at the commuting zone level>. This table presents the effect of a county's unadjusted and ancestry-adjusted pre-1500 CE potential crop yield, post-1500 CE potential crop yield, and ancestry-adjusted and unadjusted post-1500 crop yield change due to the Columbian Exchange on its excess deaths. Excess deaths for the age group "x" and older is measured as total deaths in 2020 in the age group "x" and older minus the expected deaths in 2020 in the age group "x" and older, where the expected deaths is measured by multiplying total population in the age group "x" and older in 2019 with the death rate in the age group "x" and older in 2018. All columns include state fixed effects; geographic factors that include average roughness of terrain, precipitation, temperature, and elevation; COVID-19 prevalence in neighboring counties; mobility proxies in neighboring counties; dummy variables for the urban status of each county comprising large central or large fringe metro counties and medium metro and small metro counties; mean income; proportion of males; county-level COVID-19 tests per 100,000; population density; proportion of population from a Black or African American, Native American, White, and Hispanic or Latino background; proportion of population using public transport; proportion of family and non-family households living in two or more unit structures; proportion of family and non-family households with three or more members; proportion of population in age groups younger than 19, 19–34, and 35–64 (with 65 and older as the omitted group); proportion working from home; proportion of population with an education level higher than or equal to the higher secondary level; healthcare coverage measured as the proportion of the population with two or more health insurance policies in the age groups younger than 19, 19–34, 35–64, and 65 and older, and the proportion of the population older than age 65 without any health insurance; distance to an airport with direct international flights to high-severity countries; Gini index and proportion of population below the poverty line; Social Capital Index; percentage of adult population with obesity; percentage of population who smoke as adults; 30-day risk-adjusted mortality rate; heart disease death rate; percentage of population diagnosed with diabetes among adults older than 20 years of age; contribution to the percentage change in GDP by private-services-providing industries, private-goods-providing industries, and government enterprises, and government enterprises; percentage change in annual average employment for a given year; and proportion of votes for Democrats in counties in the 2016 US presidential election. All variables are normalized by subtracting their mean and dividing by their standard deviation. Therefore, all coefficients are comparable and estimate the effect of a one standard deviation increase in the independent variable.

TABLE B.10. CAUSAL EFFECT OF MEASURES OF CROP YIELDS ON COVID-19 HOSPITALIZATION

	JULY 31, 2020 to NOVEMBER 30, 2020			
**HOSPITALIZATION PER TEST PER 100k**	**(1)**	**(2)**	**(3)**	**(4)**
Post-1500 CE Yield	-0.0949<br>[0.0848]			-0.637<br>[3.405]<br>$\langle p = 0.795 \rangle$
Post-1500 CE Cycle	-0.0236<br>[0.0509]			0.0503<br>[0.178]
Pre-1500 CE Yield		-0.103<br>[0.0898]	-0.0611<br>[0.0870]	0.816<br>[3.853]<br>$\langle p = 0.758 \rangle$
Pre-1500 CE Cycle			-0.0603<br>[0.0597]	-0.0888<br>[0.172]
Post-1500 CE Yield Ch.		0.0412<br>[0.0405]	0.0396<br>[0.0439]	0.118<br>[0.652]<br>$\langle p = 0.784 \rangle$
Post-1500 CE Cycle Ch.			0.0144<br>[0.0329]	-0.0126<br>[0.0809]
Pre-1500 CE Yield (Anc.)				-7.014<br>[7.480]<br>$\langle p = 0.441 \rangle$
Post-1500 CE Yield Ch. (Anc.)				-1.012<br>[0.256]***<br>$\langle p = 0.017 \rangle$
Pre-1500 CE Cycle (Anc.)				4.450<br>[7.690]
Post-1500 CE Cycle Ch. (Anc.)				3.101<br>[0.603]***
Observations	1,685	1,685	1,685	1,685
R-squared	0.555	0.555	0.556	0.796
Geographic	Y	Y	Y	Y
Neighbor Mob.& COVID-19 prev.	Y	Y	Y	Y
Socio-Economic, Pol., Health	Y	Y	Y	Y
State Fixed Effects	Y	Y	Y	Y

Notes: Standard errors are denoted as follows: [bootstrap standard errors account for clustering at the commuting zone level using the bootstrap command with the cluster option in STATA; that is, in this method, the sample drawn during each replication is a bootstrap sample of commuting zones]. Stars $^{***(**)[*]}$ indicate significance at the 0.01(0.05)[0.1] level. <*p*-values are generated by the wild cluster bootstrap method using the boottest command written by Roodman et al. (2019), clustered at the commuting zone level>. This table shows the effect of the unadjusted and adjusted pre-1500 CE potential crop yield, post-1500 potential crop yield, and the ancestry-adjusted and unadjusted post-1500 crop yield change due to the Columbian Exchange on COVID-19 hospitalization rate at the county level. The COVID-19 hospitalization rate is measured as the sum of reports of patients hospitalized in an adult inpatient bed who have laboratory-confirmed COVID-19 per test per 100,000 conducted in each county during the period July 31 to November 30. All columns include state fixed effects; geographic factors that include average roughness of terrain, precipitation, temperature, and elevation; COVID-19 prevalence in neighboring counties; mobility proxies in neighboring counties; dummy variables for the urban status of each county comprising large central or large fringe metro counties and medium metro and small metro counties; mean income; proportion of males; population density; proportion of population from a Black or African American, Native American, White, and Hispanic or Latino background; proportion of population using public transport; proportion of family and non-family households living in two or more unit structures; proportion of family and non-family households with three or more members; proportion of population in age groups younger than 19, 19–34, and 35–64 (with 65 and older as the omitted group); proportion working from home; proportion of population with an education level higher than or equal to the higher secondary level; healthcare coverage measured as the proportion of the population with two or more health insurance policies in the age groups younger than 19, 19–34, 35–64, and 65 and older, and the proportion of the population older than age 65 without any health insurance; distance to an airport with direct international flights to high-severity countries (2019); Gini index and proportion of population below the poverty line; Social Capital Index; percentage of adult population with obesity; percentage of population who smoke as adults (2018); 30-day risk-adjusted mortality rate; heart disease death rate (2017–2019); percentage of population diagnosed with diabetes among adults older than 20 years of age in 2017; contribution to the percentage change in GDP by private-services-providing industries (2019), private-goods-providing industries (2019), and government enterprises, and government enterprises (2019); percentage change in annual average employment for a given year (2019); and proportion of votes for Democrats in counties in the 2016 US presidential election. Additionally, there are controls measuring pre-crisis county-level hospital capacity (includes proportion of full-time staff, beds available, hospitals that were short term, hospitals operated by the Catholic Church, critical access hospitals, rural referral centers, and sole community providers; proportion with DNV Healthcare accreditation; and proportion of hospitals with a Center for Improvement in Healthcare Quality accreditation) and types of hospital reporting (comprising the proportion of critical access hospitals reporting, children's hospitals reporting, and short-term hospitals). All variables are normalized by subtracting their mean and dividing by their standard deviation. Therefore, all coefficients are comparable and estimate the effect of a one standard deviation increase in the independent variable.

Table B.11. CAUSAL EFFECT OF MEASURES OF CROP YIELDS ON INPATIENT BED USE

	**31 JULY 2020 - 30 NOVEMBER 2020**			
**AVG WEEKLY TOTAL INPATIENT BEDS USED PER HOSPITAL**	**(1)**	**(2)**	**(3)**	**(4)**
Post-1500 CE Yield	0.0335			-0.446
	[0.0346]			[1.807]
				$\langle p = 0.634 \rangle$
Post-1500 CE Cycle	0.00566			0.0417
	[0.0251]			[0.0950]
Pre-1500 CE Yield		0.0460	0.0554	0.552
		[0.0389]	[0.0422]	[2.053]
				$\langle p = 0.606 \rangle$
Pre-1500 CE Cycle			-0.00352	-0.0368
			[0.0311]	[0.0929]
Post-1500 CE Yield Ch.		0.0384*	0.0413*	0.132
		[0.0229]	[0.0235]	[0.353]
				$\langle p = 0.470 \rangle$
Post-1500 CE Cycle Ch.			0.0124	0.000880
			[0.0118]	[0.0412]
Pre-1500 CE Yield (Anc.)				-3.517
				[1.851]*
				$\langle p = 0.027 \rangle$
Post-1500 CE Yield Ch. (Anc.)				0.00608
				[0.0859]
				$\langle p = 0.923 \rangle$
Pre-1500 CE Cycle (Anc.)				3.258
				[1.928]*
Post-1500 CE Cycle Ch. (Anc.)				0.212
				[0.220]
Observations	1,694	1,694	1,694	1,694
R-squared	0.817	0.817	0.817	0.818
Geographic	Y	Y	Y	Y
Neighbor Mob.& COVID-19 prev.	Y	Y	Y	Y
Socio-Economic, Pol., Health	Y	Y	Y	Y
State Fixed Effects	Y	Y	Y	Y

Notes: Standard errors are denoted as follows: [bootstrap standard errors account for clustering at the commuting zone level using the bootstrap command with the cluster option in STATA; that is, in this method, the sample drawn during each replication is a bootstrap sample of commuting zones]. Stars $^{***(**)[*]}$ indicate significance at the $0.01(0.05)[0.1]$ level. <*p*-values are generated by the wild cluster bootstrap method using the boottest command written by Roodman et al. (2019), clustered at the commuting zone level>. This table shows the effect of the unadjusted and adjusted pre-1500 CE potential crop yield, post-1500 potential crop yield, and the ancestry-adjusted and unadjusted post-1500 crop yield change due to the Columbian Exchange on inpatient bed utilization rate at the county level. The inpatient bed utilization rate is the average weekly sum of reports of total staffed inpatient beds that are reported to be occupied per hospital in each county during the period July 31 to November 30. All columns include state fixed effects; geographic factors that include average roughness of terrain, precipitation, temperature, and elevation; COVID-19 prevalence in neighboring counties; mobility proxies in neighboring counties; dummy variables for the urban status of each county comprising large central or large fringe metro counties and medium metro and small metro counties; mean income; proportion of males; county-level COVID-19 tests per 100,000; population density; proportion of population from a Black or African American, Native American, White, and Hispanic or Latino background; proportion of population using public transport; proportion of family and non-family households living in two or more unit structures; proportion of family and non-family households with three or more members; proportion of population in age groups younger than 19, 19–34, and 35–64 (with 65 and older as the omitted group); proportion working from home; proportion of population with an education level higher than or equal to the higher secondary level; healthcare coverage measured as the proportion of the population with two or more health insurance policies in the age groups younger than 19, 19–34, 35–64, and 65 and older, and the proportion of the population older than age 65 without any health insurance; distance to an airport with direct international flights to high-severity countries; Gini index and proportion of population below the poverty line; Social Capital Index; percentage of adult population with obesity; percentage of population who smoke as adults; 30-day risk-adjusted mortality rate; heart disease death rate; percentage of population diagnosed with diabetes among adults older than 20 years of age; contribution to the percentage change in GDP by private-services-providing industries, private-goods-providing industries, and government enterprises, and government enterprises; percentage change in annual average employment for a given year; and proportion of votes for Democrats in counties in the 2016 US presidential election. Additionally, there are controls measuring pre-crisis county-level hospital capacity (includes proportion of full-time staff, beds available, hospitals that were short term, hospitals operated by the Catholic Church, critical access hospitals, rural referral centers, and sole community providers; proportion with DNV Healthcare accreditation; and proportion of hospitals with a Center for Improvement in Healthcare Quality accreditation) and types of hospital reporting (comprising the proportion of critical access hospitals reporting, children's hospitals reporting, and short-term hospitals). All variables are normalized by subtracting their mean and dividing by their standard deviation. Therefore, all coefficients are comparable and estimate the effect of a one standard deviation increase in the independent variable.

Table B.12. CAUSAL EFFECT OF LONG-TERM ORIENTATION ON MOBILITY PROXIES: VISITS TO HOBBY CENTERS, RESTAURANTS, AND MOVIE THEATERS (EXCEPT DRIVE INS)

TOTAL VISITS BY COUNTY RESIDENTS (MARCH-NOVEMBER 2020)								
**PANEL A: HOBBY CENTERS AND GAMING STORES**	**(1)**	**(2)**	**(3)**	**(4)**	**(5)**	**(6)**	**(7)**	**(8)**
	March	April	May	June	July	August	September	October
Pre-1500 CE Yield (Anc.)	-10.46	-10.90	-11.09	-11.16	-12.27	-11.89	-11.02	-10.56
	[5.534]*	[5.383]**	[6.770]	[5.588]**	[5.603]**	[5.819] **	[5.647]*	[5.837]*
	$\langle p = 0.110\rangle$	$\langle p = 0.078\rangle$	$\langle p = 0.141\rangle$	$\langle p = 0.093\rangle$	$\langle p = 0.068\rangle$	$\langle p = 0.082\rangle$	$\langle p = 0.103\rangle$	$\langle p = 0.123\rangle$
Post-1500 CE Yield Ch.(Anc.)	-0.856	-0.896	-0.869	-0.881	-0.873	-0.894	-0.828	-0.864
	[0.196]***	[0.205]***	[0.289]***	[0.202]***	[0.196]***	[0.204]***	[0.197]***	[0.208]***
	$\langle p = 0.003\rangle$	$\langle p = 0.001\rangle$	$\langle p = 0.020\rangle$	$\langle p = 0.001\rangle$	$\langle p = 0.000\rangle$	$\langle p = 0.002\rangle$	$\langle p = 0.003\rangle$	$\langle p = 0.003\rangle$
Observations	1,017	1,017	1,017	1,017	1,017	1,017	1,017	1,017
R-squared	0.888	0.846	0.833	0.880	0.881	0.876	0.879	0.872
**PANEL B: RESTAURANTS**	**(1)**	**(2)**	**(3)**	**(4)**	**(5)**	**(6)**	**(7)**	**(8)**
Pre-1500 CE Yield (Anc.)	-5.342	-5.599	-7.015	-7.795	-9.032	-8.621	-7.887	-7.827
	[4.408]	[5.110]	[5.602]	[4.998]	[4.660]*	[4.741]*	[4.814]	[4.877]
	$\langle p = 0.335\rangle$	$\langle p = 0.367\rangle$	$\langle p = 0.298\rangle$	$\langle p = 0.229\rangle$	$\langle p = 0.132\rangle$	$\langle p = 0.159\rangle$	$\langle p = 0.199\rangle$	$\langle p = 0.204\rangle$
Post-1500 CE Yield Ch. (Anc.)	-1.034	-1.016	-1.061	-1.051	-1.025	-1.044	-1.003	-1.054
	[0.181]***	[0.214]***	[0.243]***	[0.201]***	[0.183]***	[0.192]***	[0.193]***	[0.204]***
	$\langle p = 0.000\rangle$	$\langle p = 0.001\rangle$	$\langle p = 0.001\rangle$	$\langle p = 0.000\rangle$	$\langle p = 0.000\rangle$	$\langle p = 0.000\rangle$	$\langle p = 0.00\rangle$	$\langle p = 0.000\rangle$
Observations	1,822	1,822	1,822	1,822	1,822	1,822	1,822	1,822
R-squared	0.923	0.904	0.887	0.899	0.903	0.901	0.901	0.895
**PANEL C: MOVIE THEATERS (EXCEPT DRIVE INS)**	**(1)**	**(2)**	**(3)**	**(4)**	**(5)**	**(6)**	**(7)**	**(8)**
Pre-1500 CE Yield (Anc.)	-1.447	6.964	5.012	-0.0700	1.836	-0.887	-4.860	-4.179
	[6.398]	[6.179]	[5.540]	[5.188]	[4.575]	[4.525]	[5.447]	[4.806]
	$\langle p = 0.830\rangle$	$\langle p = 0.346\rangle$	$\langle p = 0.423\rangle$	$\langle p = 0.995\rangle$	$\langle p = 0.719\rangle$	$\langle p = 0.826\rangle$	$\langle p = 0.386\rangle$	$\langle p = 0.386\rangle$
Post-1500 CE Yield Ch. (Anc.)	-0.476	-0.554	-0.472	-0.359	-0.483	-0.576	-0.461	-0.468
	[0.247]*	[0.255]**	[0.225]**	[0.242]	[0.211]**	[0.181]***	[0.211]**	[0.192]**
	$\langle p = 0.129\rangle$	$\langle p = 0.062\rangle$	$\langle p = 0.053\rangle$	$\langle p = 0.236\rangle$	$\langle p = 0.073\rangle$	$\langle p = 0.006\rangle$	$p = 0.0561$	$\langle p = 0.025\rangle$
Observations	630	630	630	630	630	630	630	630
R-squared	0.826	0.815	0.820	0.805	0.822	0.800	0.774	0.764
Geographic	Y	Y	Y	Y	Y	Y	Y	Y
Neighbor Mob.& COVID-19 prev.	Y	Y	Y	Y	Y	Y	Y	Y
Socio-Economic, Pol., Health	Y	Y	Y	Y	Y	Y	Y	Y
State-Fixed Effects	Y	Y	Y	Y	Y	Y	Y	Y

Notes: Standard errors are denoted as follows: [bootstrap standard errors account for clustering at the commuting zone level using the bootstrap command with the cluster option in STATA; that is, in this method, the sample drawn during each replication is a bootstrap sample of commuting zones]. Stars $^{***(**)[*]}$ indicate significance at the $0.01(0.05)[0.1]$ level. <p-values are generated by the wild cluster bootstrap method using the boottest command written by Roodman et al. (2019), clustered at the commuting zone level>. Each column reports monthly estimates from separate regressions, following Equation 1, which includes the ancestry-adjusted pre-1500 CE crop yield and ancestry-adjusted post-1500 CE yield change and accounts for ancestry-adjusted crop growth cycle and its changes; unobserved state-varying attributes via state fixed effects; geographic factors that include average roughness of terrain, precipitation, temperature, and elevation, and county-level COVID-19 prevalence in neighboring counties in the month when the regression is estimated, which is measured as the confirmed COVID-19 cases per test per 100,000 averaged over all neighboring counties; COVID-19 tests per 100,000 conducted in the county in the month when the regression is estimated; mobility proxies in neighboring counties; dummy variables for the urban status of each county comprising large central or large fringe metro counties and medium metro and small metro counties; mean income; proportion of males; population density; proportion of population from a Black or African American, Native American, White, and Hispanic or Latino background; proportion of population using public transport; proportion of family and non-family households living in two or more unit structures; proportion of family and non-family households with three or more members; proportion of population in age groups younger than 19, 19–34, and 35–64 (with 65 and older as the omitted group); proportion working from home; proportion of population with an education level higher than or equal to the higher secondary level; healthcare coverage measured as the proportion of the population with two or more health insurance policies in the age groups younger than 19, 19–34, 35–64, and 65 and older, and the proportion of the population older than age 65 without any health insurance; distance to an airport with direct international flights to high-severity countries; Gini index and proportion of population below the poverty line; Social Capital Index; percentage of adult population with obesity; percentage of population who smoke as adults; 30-day risk-adjusted mortality rate; heart disease death rate; percentage of population diagnosed with diabetes among adults older than 20 years of age; contribution to the percentage change in GDP by private-services-providing industries, private-goods-providing industries, and government enterprises, and government enterprises; percentage change in annual average employment for a given year; and proportion of votes for Democrats in counties in the 2016 US presidential election. Predicted ancestral compositions in each county using the instruments from Burchardi, Chaney and Hassan (2019) and the post-1500 World Migration Matrix of Putterman and Weil (2010) were used to adjust crop yield measures. Our outcome variables are the total number of visits in each county to three POIs obtained from SafeGraph's Patterns data. The POIs are hobby, toy, and games stores in panel A; restaurants and other eating places (i.e., full-service restaurants, limited-service restaurants, cafeterias, grill buffets and buffets, and snacks and non-alcoholic beverage bars) in panel B; and motion picture theaters (excluding drive-ins) in panel C. All variables are normalized by subtracting their mean and dividing by their standard deviation. Therefore, all coefficients are comparable and estimate the effect of a one standard deviation increase in the independent variable.

Table B.13. CAUSAL EFFECT OF LONG-TERM ORIENTATION ON MOBILITY PROXIES: VISITS TO CLOTHING STORES AND FITNESS CENTERS

	TOTAL VISITS BY COUNTY RESIDENTS (MARCH-NOVEMBER 2020)							
**PANEL A: CLOTHING STORES**	**(1)**	**(2)**	**(3)**	**(4)**	**(5)**	**(6)**	**(7)**	**(8)**
	March	April	May	June	July	August	September	October
Pre-1500 CE Yield (Anc.)	-8.658	-8.482	-11.33	-10.67	-10.76	-10.87	-10.47	-10.38
	[3.746]**	[3.908]**	[4.114]***	[3.190]***	[2.998]***	[3.227]***	[3.400]***	[3.497]***
	$\langle p = 0.024\rangle$	$\langle p = 0.0581\rangle$	$\langle p = 0.029\rangle$	$\langle p = 0.008\rangle$	$\langle p = 0.000\rangle$	$\langle p = 0.005\rangle$	$\langle p = 0.007\rangle$	$\langle p = 0.006\rangle$
Post-150 CE Yield Ch. (Anc.)	-1.127	-1.180	-1.246	-1.154	-1.113	-1.111	-1.082	-1.139
	[0.134]***	[0.142]***	[0.201]***	[0.122]***	[0.119]***	[0.130]***	[0.133]***	[0.137]***
	$\langle p = 0.000\rangle$	$\langle p = 0.000\rangle$	$\langle p = 0.000\rangle$	$\langle p = 0.000\rangle$	$\langle p = 0.000\rangle$	$\langle p = 0.000\rangle$	$\langle p = 0.000\rangle$	$\langle p = 0.000\rangle$
Observations	1,404	1,404	1,404	1,404	1,404	1,404	1,404	1,404
R-squared	0.925	0.914	0.904	0.933	0.933	0.930	0.926	0.922
**PANEL B: FITNESS CENTERS**	**(1)**	**(2)**	**(3)**	**(4)**	**(5)**	**(6)**	**(7)**	**(8)**
Pre-1500 CE Yield (Anc.)	-7.009	-7.594	-10.57	-11.19	-12.37	-12.65	-12.53	-12.58
	[3.240]**	[3.319]**	[4.466]**	[4.508]**	[4.251]***	[4.606]***	[5.173]**	[5.142]**
	$\langle p = 0.009\rangle$	$\langle p = 0.000\rangle$	$\langle p = 0.010\rangle$	$\langle p = 0.007\rangle$	$\langle p = 0.000\rangle$	$\langle p = 0.000\rangle$	$\langle p = 0.001\rangle$	$\langle p = 0.005\rangle$
Post-1500 Yield Ch. (Anc.)	-0.962	-0.970	-1.034	-1.031	-1.013	-1.013	-0.965	-1.008
	[0.126]***	[0.130]***	[0.174]***	[0.160]***	[0.144]***	[0.154]***	[0.168]***	[0.174]***
	$\langle p = 0.000\rangle$	$\langle p = 0.000\rangle$	$\langle p = 0.000\rangle$	$\langle p = 0.000\rangle$	$\langle p = 0.000\rangle$	$\langle p = 0.000\rangle$	$\langle p = 0.000\rangle$	$\langle p = 0.000\rangle$
Observations	1,460	1,460	1,460	1,460	1,460	1,460	1,460	1,460
R-squared	0.926	0.927	0.889	0.883	0.906	0.900	0.886	0.882
Geographic	Y	Y	Y	Y	Y	Y	Y	Y
Neighbor Mob.& COVID-19 prev.	Y	Y	Y	Y	Y	Y	Y	Y
Socio-Economic, Pol., Health	Y	Y	Y	Y	Y	Y	Y	Y
State Fixed Effects	Y	Y	Y	Y	Y	Y	Y	Y

Notes: Standard errors are denoted as follows: [bootstrap standard errors account for clustering at the commuting zone level using the bootstrap command with the cluster option in STATA; that is, in this method, the sample drawn during each replication is a bootstrap sample of commuting zones]. Stars $^{***(**)[*]}$ indicate significance at the 0.01(0.05)[0.1] level. <*p*-values are generated by the wild cluster bootstrap method using the boottest command written by Roodman et al. (2019), clustered at the commuting zone level>. Each column reports monthly estimates from separate regressions, following Equation 1, which includes the ancestry-adjusted pre-1500 CE crop yield and ancestry-adjusted post-1500 CE yield change and accounts for ancestry-adjusted crop growth cycle and its changes; unobserved state-varying attributes via state fixed effects; geographic factors that include average roughness of terrain, precipitation, temperature, and elevation, and county-level COVID-19 prevalence in neighboring counties in the month when the regression is estimated, which is measured as the confirmed COVID-19 cases per test per 100,000 averaged over all neighboring counties; COVID-19 tests per 100,000 conducted in the county in the month when the regression is estimated; mobility proxies in neighboring counties; dummy variables for the urban status of each county comprising large central or large fringe metro counties and medium metro and small metro counties; mean income; proportion of males; population density; proportion of population from a Black or African American, Native American, White, and Hispanic or Latino background; proportion of population using public transport; proportion of family and non-family households living in two or more unit structures; proportion of family and non-family households with three or more members; proportion of population in age groups younger than 19, 19–34, and 35–64 (with 65 and older as the omitted group); proportion working from home; proportion of population with an education level higher than or equal to the higher secondary level; healthcare coverage measured as the proportion of the population with two or more health insurance policies in the age groups younger than 19, 19–34, 35–64, and 65 and older, and the proportion of the population older than age 65 without any health insurance; distance to an airport with direct international flights to high-severity countries; Gini index and proportion of population below the poverty line; Social Capital Index; percentage of adult population with obesity; percentage of population who smoke as adults; 30-day risk-adjusted mortality rate; heart disease death rate; percentage of population diagnosed with diabetes among adults older than 20 years of age; contribution to the percentage change in GDP by private-services-providing industries, private-goods-providing industries, and government enterprises, and government enterprises; percentage change in annual average employment for a given year; and proportion of votes for Democrats in counties in the 2016 US presidential election. Predicted ancestral compositions in each county using the instruments from Burchardi, Chaney and Hassan (2019) and the post-1500 World Migration Matrix of Putterman and Weil (2010) were used to adjust crop yield measures. Our outcome variables are the total number of visitors from each county to two POIs from the beginning of every month to the end of the month obtained from SafeGraph's Patterns data. The POIs are clothing stores in panel A and fitness and recreational centers in panel B. All variables are normalized by subtracting their mean and dividing by their standard deviation. Therefore, all coefficients are comparable and estimate the effect of a one standard deviation increase in the independent variable.

Table B.14. CAUSAL EFFECT OF LONG-TERM ORIENTATION ON MOBILITY PROXIES: GOOGLE MOBILITY REPORTS

GOOGLE MOBILITY REPORTS MARCH-OCTOBER 2020								
**PANEL A: VISITS TO RECREATION CENTERS**	**(1)**	**(2)**	**(3)**	**(4)**	**(5)**	**(6)**	**(7)**	**(8)**
	March	April	May	June	July	August	September	October
Pre-1500 CE Yield (Anc.)	-0.911	-1.352	-3.212	-4.193	-2.653	-3.405	-4.625	-3.778
	[1.905]	[1.512]	[2.287]	[2.899]	[2.881]	[2.945]	[2.694]*	[2.503]
	$\langle p = 0.585 \rangle$	$\langle p = 0.290 \rangle$	$\langle p = 0.139 \rangle$	$\langle p = 0.111 \rangle$	$\langle p = 0.304 \rangle$	$\langle p = 0.215 \rangle$	$\langle p = 0.0561 \rangle$	$\langle p = 0.083 \rangle$
Post-1500 CE Yield Ch. (Anc.)	-0.180	-0.0995	-0.0288	0.0552	0.0223	0.0326	0.0463	-0.0164
	[0.103]*	[0.0846]	[0.0997]	[0.133]	[0.137]	[0.140]	[0.125]	[0.126]
	$\langle p = 0.025 \rangle$	$\langle p = 0.118 \rangle$	$\langle p = 0.739 \rangle$	$\langle p = 0.661 \rangle$	$\langle p = 0.858 \rangle$	$\langle p = 0.811 \rangle$	$\langle p = 0.691 \rangle$	$\langle p = 0.877 \rangle$
Observations	1,037	1,037	1,037	1,037	1,037	1,037	1,037	1,037
R-squared	0.781	0.805	0.728	0.563	0.507	0.514	0.569	0.602
**PANEL B: HOURS SPENT AWAY FROM HOME**	**(1)**	**(2)**	**(3)**	**(4)**	**(5)**	**(6)**	**(7)**	**(8)**
Pre-1500 CE Yield (Anc.)	-1.159	-1.498	-3.008	-3.614	-2.962	-0.884	-2.086	-4.853
	[1.803]	[1.814]	[2.240]	[2.339]	[2.535]	[2.661]	[2.141]	[2.257]**
	$\langle p = 0.423 \rangle$	$\langle p = 0.315 \rangle$	$\langle p = 0.141 \rangle$	$\langle p = 0.059 \rangle$	$\langle p = 0.152 \rangle$	$p = 0.718$	$\langle p = 0.249 \rangle$	$\langle p = 0.040 \rangle$
Post-1500 CE Yield Ch. (Anc.)	-0.0183	-0.0434	-0.0107	0.0348	0.0898	0.0259	-0.00334	0.00482
	[0.0848]	[0.0851]	[0.0955]	[0.0976]	[0.0988]	[0.0917]	[0.0824]	[0.0916]
	$\langle p = 0.771 \rangle$	$\langle p = 0.554 \rangle$	$\langle p = 0.907 \rangle$	$\langle p = 0.666 \rangle$	$\langle p = 0.235 \rangle$	$\langle p = 0.698 \rangle$	$\langle p = 0.971 \rangle$	$\langle p = 0.939 \rangle$
Observations	890	890	890	890	890	890	890	890
R-squared	0.846	0.892	0.878	0.867	0.859	0.838	0.893	0.894
Geographic	Y	Y	Y	Y	Y	Y	Y	Y
Neighbor Mob.& COVID-19 prev.	Y	Y	Y	Y	Y	Y	Y	Y
Socio-Economic, Pol., Health	Y	Y	Y	Y	Y	Y	Y	Y
State Fixed Effects	Y	Y	Y	Y	Y	Y	Y	Y

Notes: Standard errors are denoted as follows: [bootstrap standard errors account for clustering at the commuting zone level using the bootstrap command with the cluster option in STATA; that is, in this method, the sample drawn during each replication is a bootstrap sample of commuting zones]. Stars $^{***(**)[*]}$ indicate significance at the $0.01(0.05)[0.1]$ level. <p-values are generated by the wild cluster bootstrap method using the boottest command written by Roodman et al. (2019), clustered at the commuting zone level>. This table presents the effect of a county's ancestry-adjusted pre-1500 CE potential crop yield and ancestry-adjusted post-1500 crop yield change due to the Columbian Exchange on its mobility proxies in Google Community Mobility Reports. In panel A, the outcome variable is the percentage change in visits to places such as restaurants, cafes, shopping centers, theme parks, museums, libraries, and movie theaters; in panel B, the outcome variable is the percentage change in the duration of stays in places outside of residence. Both outcome variables are measured as percentage changes relative to the pre-crisis level (i.e., between January 3, 2020 and February 6, 2020). The monthly data are from the 15th of every month to the 14th of the following month. Each column reports monthly estimates from separate regressions, following Equation 1, which includes the ancestry-adjusted pre-1500 CE crop yield and ancestry-adjusted post-1500 CE yield change and accounts for ancestry-adjusted crop growth cycle and its changes; unobserved state-varying attributes via state fixed effects; geographic factors that include average roughness of terrain, precipitation, temperature, and elevation, and county-level COVID-19 prevalence in neighboring counties in the month when the regression is estimated, which is measured as the confirmed COVID-19 cases per test per 100,000 averaged over all neighboring counties; COVID-19 tests per 100,000 conducted in the county in the month when the regression is estimated; the percentage change in hours spent away from the home averaged across neighboring counties in the month when the regression is estimated; dummy variables for the urban status of each county comprising large central or large fringe metro counties and medium metro and small metro counties; mean income; proportion of males; population density; proportion of population from a Black or African American, Native American, White, and Hispanic or Latino background; proportion of population using public transport; proportion of family and non-family households living in two or more unit structures; proportion of family and non-family households with three or more members; proportion of population in age groups younger than 19, 19–34, and 35–64 (with 65 and older as the omitted group); proportion working from home; proportion of population with an education level higher than or equal to the higher secondary level; healthcare coverage measured as the proportion of the population with two or more health insurance policies in the age groups younger than 19, 19–34, 35–64, and 65 and older, and the proportion of the population older than age 65 without any health insurance; distance to an airport with direct international flights to high-severity countries; Gini index and proportion of population below the poverty line; Social Capital Index; percentage of adult population with obesity; percentage of population who smoke as adults; 30-day risk-adjusted mortality rate; heart disease death rate; percentage of population diagnosed with diabetes among adults older than 20 years of age; contribution to the percentage change in GDP by private-services-providing industries, private-goods-providing industries, and government enterprises, and government enterprises; percentage change in annual average employment for a given year; and proportion of votes for Democrats in counties in the 2016 US presidential election. Predicted ancestral compositions in each county using the instruments from Burchardi, Chaney and Hassan (2019) and the post-1500 World Migration Matrix of Putterman and Weil (2010) were used to adjust crop yield measures. All variables are normalized by subtracting their mean and dividing by their standard deviation. Therefore, all coefficients are comparable and estimate the effect of a one standard deviation increase in the independent variable.

Table B.15. DYNAMIC CAUSAL EFFECT OF LONG-TERM ORIENTATION ON VISITORS/VISITS TO RESTAURANTS

	MOBILITY BEHAVIOR OF COUNTY RESIDENTS (FEB-OCTOBER 2020)							
**RESTAURANTS AND OTHER EATING PLACES**	**(1)** Visitors	**(2)** Visitors	**(3)** Visits	**(4)** Visits	**(5)** Visitors	**(6)** Visitors	**(7)** Visits	**(8)** Visits
High Pre-1500 CE Yield (Anc.)*Mar	-0.127***	-0.150***	-0.146***	-0.175***				
	(0.0000)	(0.0000)	(0.0000)	(0.0000)				
High Pre-1500 CE Yield (Anc.)*Apr	-0.261***	-0.307***	-0.275***	-0.330***				
	(0.0000)	(0.0000)	(0.0000)	(0.0000)				
High Pre-1500 CE Yield (Anc.)*May	-0.197***	-0.267***	-0.214***	-0.298***				
	(0.0000)	(0.0000)	(0.0000)	(0.0000)				
High Pre-1500 CE Yield (Anc.)*Jun	-0.155***	-0.247***	-0.175***	-0.285***				
	(0.0000)	(0.0000)	(0.0000)	(0.0000)				
High Pre-1500 CE Yield (Anc.)*Jul	-0.150***	-0.264***	-0.167***	-0.304***				
	(0.0010)	(0.0000)	(0.0000)	(0.0000)				
High Pre-1500 CE Yield (Anc.)*Aug	-0.127***	-0.260***	-0.147***	-0.306***				
	(0.0000)	(0.0000)	(0.0000)	(0.0000)				
High Pre-1500 CE Yield (Anc.)*Sep	-0.155***	-0.312***	-0.173***	-0.362***				
	(0.0000)	(0.0000)	(0.0000)	(0.0000)				
High Pre-1500 CE Yield (Anc.)*Oct	-0.140***	-0.321***	-0.157***	-0.374***				
	(0.0000)	(0.0000)	(0.0000)	(0.0000)				
High Post-1500 CE Yield Ch. (Anc.)*Mar					-0.0479	-0.0742**	-0.0656**	-0.0956***
					(0.1041)	(0.0280)	(0.0400)	(0.0080)
High Post-1500 CE Yield Ch. (Anc.)*Apr					-0.0859	-0.140**	-0.105*	-0.166***
					(0.1241)	(0.0320)	(0.0651)	(0.0220)
High Post-1500 CE Yield Ch. (Anc.)*May					-0.0745**	-0.155***	-0.0922**	-0.184**
					(0.0470)	(0.0030)	(0.0170)	(0.0010)
High Post-1500 CE Yield Ch. (Anc.)*Jun					-0.0897**	-0.197***	-0.108***	-0.230***
					(0.0120)	(0.0010)	(0.0050)	((0.0000))
High Post-1500 CE Yield Ch. (Anc.)*Jul					-0.0990***	-0.231***	-0.117***	-0.266***
					(0.0090)	(0.0010)	(0.0070)	(0.0010)
High Post-1500 CE Yield Ch. (Anc.)*Aug					-0.0763**	-0.230***	-0.0956***	-0.270***
					(0.0120)	(0.0000)	(0.0040)	(0.0000)
High Post-1500 CE Yield Ch. (Anc.)*Sep					-0.0810**	-0.263***	-0.0995***	-0.306***
					(0.0140)	(0.0010)	(0.0080)	(0.0010)
High Post-1500 CE Yield Ch. (Anc.)*Oct					-0.0774***	-0.286***	-0.0948***	-0.331***
					(0.0090)	(0.0000)	(0.0040)	(0.0000)
Observations	6,515	6,515	6,515	6,515	6,515	6,515	6,515	6,515
R-squared	0.258	0.267	0.253	0.264	0.247	0.256	0.242	0.252
Number of counties	769	769	769	769	769	769	769	769
State*Trends	No	Yes	No	Yes	No	Yes	No	Yes
Geographic Controls	Yes	Yes	Yes	Yes	Yes	Yes	Yes	Yes
Mobility Neighboring County	Yes	Yes	Yes	Yes	Yes	Yes	Yes	Yes

Notes:"()" reports the p-values of the wild cluster bootstrap method using the post-estimation command boottest, clustered at the commuting zone level. Stars $^{***(**)[*]}$ indicate significance at the $0.01(0.05)[0.1]$ level. This table presents the effect of the ancestry-adjusted high pre-1500 CE potential crop yield (where ancestry-adjusted high pre-1500 CE potential crop yield is a dummy variable that equals 1 if the ancestry-adjusted potential crop yield is above the sample median) and the ancestry-adjusted high post-1500 CE potential crop yield change during the Columbian Exchange (where ancestry-adjusted high post-1500 potential yield change is a dummy variable that equals 1 if the potential crop yield change is above the sample median) on monthly total county visitors and total county visits to restaurants and other eating places (i.e., full-service restaurants, limited-service restaurants, cafeterias, grill buffets and buffets, and snacks and non-alcoholic beverage bars) obtained from SafeGraph's Patterns data. The outcome variables in columns (1), (2), (5), and (6) are the total number of visitors from each county visiting restaurants and other eating places. The outcome variables in columns (3), (4), (7), and (8) are the total number of visits by county residents to county restaurants and other eating places, which are calculated by multiplying the average visits per visitor to restaurants and other eating places in a given county with total residents of the given county visiting restaurants and other eating places. The reported effects are relative to the baseline variable in February 2020, based on Equation 2. All columns control for COVID-19 cases in neighboring counties, monthly mobility proxies in neighboring counties, temperature, and precipitation. Columns (2), (4), (6), and (8) control for state-specific trends. Predicted ancestral compositions in each county using the instruments from Burchardi, Chaney and Hassan (2019) and the post-1500 World Migration Matrix of Putterman and Weil (2010) were used to adjust crop yield measures. All variables are normalized by subtracting their mean and dividing by their standard deviation. Therefore, all coefficients are comparable and estimate the effect of a one standard deviation increase in the independent variable.

Table B.16. DYNAMIC CAUSAL EFFECT OF LONG-TERM ORIENTATION ON VISITORS/VISITS TO MOVIE THEATERS (EXCEPT DRIVE-INS)

	MOBILITY BEHAVIOR OF COUNTY RESIDENTS (FEB-OCTOBER 2020)							
**MOVIE THEATERS (EXCEPT DRIVE-INS)**	**(1)** Visitors	**(2)** Visitors	**(3)** Visits	**(4)** Visits	**(5)** Visitors	**(6)** Visitors	**(7)** Visits	**(8)** Visits
High Pre-1500 CE Yield (Anc.)*Mar	-0.377***	-0.579***	-0.346***	-0.541***				
	(0.0000)	(0.0000)	(0.0000)	(0.0000)				
High Pre-1500 CE Yield (Anc.)*Apr	-0.545***	-0.956***	-0.502***	-0.893***				
	(0.0010)	(0.0000)	(0.0000)	(0.0000)				
High Pre-1500 CE Yield (Anc.)*May	-0.554***	-1.166***	-0.504***	-1.091***				
	(0.0000)	(0.0000)	(0.0010)	(0.0000)				
High Pre-1500 CE Yield (Anc.)*Jun	-0.577***	-1.381***	-0.523***	-1.292***				
	(0.0000)	(0.0000)	(0.0000)	(0.0000)				
High Pre-1500 CE Yield (Anc.)*Jul	-0.599***	-1.595***	-0.540***	-1.492***				
	(0.0010)	(0.0000)	(0.0010)	(0.0000)				
High Pre-1500 CE Yield (Anc.)*Aug	-0.555***	-1.748***	-0.499***	-1.635***				
	(0.0000)	(0.0000)	(0.0000)	(0.0000)				
High Pre-1500 CE Yield (Anc.)*Sep	-0.556***	-1.958***	-0.500***	-1.838				
	(0.0000)	(0.0000)	(0.0000)	(0.0000)				
High Pre-1500 CE Yield (Anc.)*Oct	-0.506***	-2.107***	-0.459***	-1.989***				
	(0.0000)	(0.0000)	(0.0000)	(0.0000)				
High Post-1500 CE Yield Ch. (Anc.)*Mar					-0.192**	-0.356***	-0.162*	-0.321***
					(0.0290)	(0.0010)	(0.0621)	(0.0030)
High Post-1500 CE Yield Ch. (Anc.)*Apr					-0.177	-0.524**	-0.142	-0.472**
					(0.2272)	(0.0120)	(0.3273)	(0.0180)
High Post-1500 CE Yield Ch. (Anc.)*May					-0.204	-0.709***	-0.164	-0.649***
					(0.1692)	(0.0020)	(0.2442)	(0.0040)
High Post-1500 CE Yield Ch. (Anc.)*Jun					-0.314**	-0.974**	-0.278*	-0.911***
					(0.0300)	(0.0030)	(0.0531)	(0.0030)
High Post-1500 CE Yield Ch. (Anc.)*Jul					-0.378**	-1.190***	-0.344**	-1.120***
					(0.0130)	(0.0000)	(0.0170)	(0.0000)
High Post-1500 CE Yield Ch. (Anc.)*Aug					-0.329***	-1.313***	-0.297**	-1.231***
					(0.0090)	(0.0000)	(0.0160)	(0.0000)
High Post-1500 CE Yield Ch. (Anc.)*Sep					-0.321**	-1.482***	-0.294**	-1.399***
					(0.0180)	(0.0010)	(0.0230)	(0.0010)
High Post-1500 CE Yield Ch. (Anc.)*Oct					-0.280**	-1.609***	-0.251**	-1.518***
					(0.0150)	(0.0000)	(0.0350)	(0.0000)
Observations	6,515	6,515	6,515	6,515	6,515	6,515	6,515	6,515
R-squared	0.262	0.311	0.254	0.301	0.254	0.288	0.247	0.280
Number of counties	769	769	769	769	769	769	769	769
State*Trends	No	Yes	No	Yes	No	Yes	No	Yes
Geographic Controls	Yes	Yes	Yes	Yes	Yes	Yes	Yes	Yes
Mobility Neighboring County	Yes	Yes	Yes	Yes	Yes	Yes	Yes	Yes

Notes: "()" reports the p-values of the wild cluster bootstrap method using the post-estimation command boottest, clustered at the commuting zone level. Stars $^{***(**)[*]}$ indicate significance at the $0.01(0.05)[0.1]$ level. This table presents the effect of the ancestry-adjusted high pre-1500 CE potential crop yield (where ancestry-adjusted high pre-1500 CE potential crop yield is a dummy variable that equals 1 if the ancestry-adjusted potential crop yield is above the sample median) and the ancestry-adjusted high post-1500 potential crop yield change during the Columbian Exchange (where ancestry-adjusted high post-1500 potential yield change is a dummy variable that equals 1 if the potential crop yield change is above the sample median) on monthly total county visitors and total county visits to movie theaters obtained from SafeGraph's Patterns data. The outcome variables in columns (1), (2), (5), and (6) are the total number of visitors from each county visiting movie theaters. The outcome variables in columns (3), (4), (7), and (8) are the total number of visits by county residents to movie theaters, which is calculated by multiplying the average visits per visitor to movie theaters in each county with the total visitors by county to movie theaters. The reported effects are relative to the baseline variable in February 2020, based on Equation 2. All columns control for COVID-19 cases in neighboring counties, monthly mobility proxies in neighboring counties, temperature, and precipitation. Columns (2), (4), (6), and (8) control for state-specific time trends. Predicted ancestral compositions in each county using the instruments from Burchardi, Chaney and Hassan (2019) and the post-1500 World Migration Matrix of Putterman and Weil (2010) were used to adjust crop yield measures. All variables are normalized by subtracting their mean and dividing by their standard deviation. Therefore, all coefficients are comparable and estimate the effect of a one standard deviation increase in the independent variable.

TABLE B.17. DYNAMIC CAUSAL EFFECT OF LONG-TERM ORIENTATION ON VISITORS/VISITS TO CLOTHING STORES

	MOBILITY BEHAVIOR OF COUNTY RESIDENTS (FEB-OCTOBER 2020)							
**CLOTHING STORES**	**(1)** Visitors	**(2)** Visitors	**(3)** Visits	**(4)** Visits	**(5)** Visitors	**(6)** Visitors	**(7)** Visits	**(8)** Visits
High Pre-1500 CE Yield (Anc.)*Mar	-0.182***	-0.179***	-0.174***	-0.180***				
	(0.000)	(0.0000)	(0.0010)	(0.0010)				
High Pre-1500 CE Yield (Anc.)*Apr	-0.362***	-0.356***	-0.332***	-0.342***				
	(0.000)	(0.0000)	((0.0000)	(0.0000)				
High Pre-1500 CE Yield (Anc.)*May	-0.296***	-0.286***	-0.276***	-0.292***				
	(0.000)	(0.0000)	(0.0000)	(0.0000)				
High Pre-1500 CE Yield (Anc.)*Jun	-0.186***	-0.177***	-0.186***	-0.209***				
	(0.000)	(0.0000)	(0.0000)	(0.0000)				
High Pre-1500 CE Yield (Anc.)*Jul	-0.172***	-0.162***	-0.174***	-0.204***				
	(0.000)	(0.0020)	(0.0010)	(0.0010)				
High Pre-1500 CE Yield (Anc.)*Aug	-0.147***	-0.134***	-0.155***	-0.188***				
	(0.000)	(0.0000)	(0.0000)	(0.0000)				
High Pre-1500 CE Yield (Anc.)*Sep	-0.174***	-0.158***	-0.177***	-0.217***				
	(0.000)	(0.0000)	(0.0000)	(0.0000)				
High Pre-1500 CE Yield (Anc.)*Oct	-0.152***	-0.135**	-0.154***	-0.201***				
	(0.000)	(0.0090)	(0.0000)	(0.0000)				
High Post-1500 CE Yield Ch. (Anc.)*Mar					-0.0766*	-0.0782*	-0.0719*	-0.0791*
					(0.0521)	(0.0541)	(0.0751)	(0.0561)
High Post-1500 CE Yield Ch. (Anc.)*Apr					-0.122	-0.125	-0.107	-0.121
					(0.1081)	(0.1031)	(0.1331)	(0.1111)
High Post-1500 CE Yield Ch. (Anc.)*May					-0.122**	-0.128**	-0.106**	-0.128**
					(0.0200)	(0.0240)	(0.0420)	(0.0290)
High Post-1500 CE Yield Ch. (Anc.)*Jun					-0.112**	-0.124**	-0.107**	-0.139**
					(0.0140)	(0.0240)	(0.0220)	(0.0180)
High Post-1500 CE Yield Ch. (Anc.)*Jul					-0.122**	-0.139**	-0.119**	-0.160**
					(0.0080)	(0.0160)	(0.0140)	(0.0120)
High Post-1500 CE Yield Ch. (Anc.)*Aug					-0.101**	-0.118**	-0.0980**	-0.142**
					(0.0090)	(0.0320)	(0.0160)	(0.0110)
High Post-1500 CE Yield Ch. (Anc.)*Sep					-0.0946**	-0.112*	-0.0941**	-0.145**
					(0.0130)	(0.0731)	(0.0220)	(0.0330)
High Post-1500 CE Yield Ch. (Anc.)*Oct					-0.0874**	-0.109	-0.0863**	-0.146**
					(0.0160)	(0.1481)	(0.0320)	(0.0531)
Observations	6,515	6,515	6,515	6,515	6,515	6,515	6,515	6,515
R-squared	0.256	0.257	0.236	0.237	0.243	0.244	0.224	0.226
Number of counties	769	769	769	769	769	769	769	769
State*Trends	No	Yes	No	Yes	No	Yes	No	Yes
Geographic Controls	Yes	Yes	Yes	Yes	Yes	Yes	Yes	Yes
Mobility Neighboring County	Yes	Yes	Yes	Yes	Yes	Yes	Yes	Yes

Notes:"()" reports the p-values of the wild cluster bootstrap method using the post-estimation command boottest. Stars$^{***(**)[*]}$ indicate significance at the 0.01(0.05)[0.1] level. This table presents the effect of the ancestry-adjusted high pre-1500 CE potential crop yield (where ancestry-adjusted high pre-1500 CE potential crop yield is a dummy variable that equals 1 if the ancestry-adjusted potential crop yield is above the sample median) and the ancestry-adjusted high post-1500 potential crop yield change during the Columbian Exchange (where ancestry-adjusted high post-1500 potential yield change is a dummy variable that equals 1 if the potential crop yield change is above the sample median) on monthly total county visitors and total county visits to clothing stores obtained from SafeGraph's Patterns data. The outcome variables are columns (1), (2), (5), and (6) are the total number of visitors from each county visiting clothing stores. The outcome variables in columns (3), (4), (7), and (8) are the total number of visits by county residents to clothing stores, which are calculated by multiplying the average visits per visitor to clothing stores in each county with the total number of county residents visiting clothing stores. The reported effects are relative to the baseline variable in February 2020, based on Equation-2. All columns control for COVID-19 cases in neighboring counties, monthly mobility proxies in neighboring counties, temperature, and precipitation. Columns (2), (4), (6) and (8) control for state-specific time trends. Predicted ancestral compositions in each county using the instruments from Burchardi, Chaney and Hassan (2019) and the post-1500 World Migration Matrix of Putterman and Weil (2010) were used to adjust crop yield measures. All variables are normalized by subtracting their mean and dividing by their standard deviation. Therefore, all coefficients are comparable and estimate the effect of a one standard deviation increase in the independent variable. Standard errors are clustered at the commuting zone level.

Table B.18. DYNAMIC CAUSAL EFFECT OF LONG-TERM ORIENTATION ON VISITORS/VISITS TO FITNESS AND RECREATION CENTERS

	MOBILITY BEHAVIOR OF COUNTY RESIDENTS (FEB-OCTOBER 2020)							
**FITNESS AND RECREATION CENTERS**	**(1)** Visitors	**(2)** Visitors	**(3)** Visits	**(4)** Visits	**(5)** Visitors	**(6)** Visitors	**(7)** Visits	**(8)** Visits
High Pre-1500 CE Yield (Anc.)*Mar	-0.197***	-0.249***	-0.256***	-0.312***				
	(0.0000)	(0.0000)	(0.0000)	(0.0000)				
High Pre-1500 CE Yield (Anc.)*Apr	-0.458***	-0.559***	-0.490***	-0.595***				
	(0.0000)	(0.0000)	(0.0000)	(0.0000)				
High Pre-1500 CE Yield (Anc.)*May	-0.384***	-0.539***	-0.428***	-0.591***				
	(0.0000)	(0.0000)	(0.0000)	(0.0000)				
High Pre-1500 CE Yield (Anc.)*Jun	-0.326***	-0.530***	-0.374***	-0.589***				
	(0.0000)	(0.0000)	(0.0000)	(0.0000)				
High Pre-1500 CE Yield (Anc.)*Jul	-0.318***	-0.571***	-0.352***	-0.619***				
	(0.0000)	(0.0000)	(0.0000)	(0.0000)				
High Pre-1500 CE Yield (Anc.)*Aug	-0.290***	-0.586***	-0.335***	-0.644***				
	(0.0000)	(0.0000)	(0.0000)	(0.0000)				
High Pre-1500 CE Yield (Anc.)*Sep	-0.288***	-0.639***	-0.312***	-0.680				
	(0.0000)	(0.0000)	(0.0000)	(0.0000)				
High Pre-1500 CE Yield (Anc.)*Oct	-0.266***	-0.668***	-0.286***	-0.708***				
	(0.0000)	(0.0000)	(0.0000)	(0.0000)				
High Post-1500 CE Yield Ch. (Anc.)*Mar					-0.0932**	-0.135***	-0.124**	-0.167***
					(0.0170)	(0.0040)	(0.0230)	(0.0050)
High Post-1500 CE Yield Ch. (Anc.)*Apr					-0.182**	-0.269***	-0.187*	-0.272**
					(0.0390)	(0.0100)	(0.0531)	(0.0200)
High Post-1500 CE Yield Ch. (Anc.)*May					-0.162***	-0.292***	-0.174**	-0.304***
					(0.0090)	(0.0000)	(0.0160)	(0.0020)
High Post-1500 CE Yield Ch. (Anc.)*Jun					-0.207***	-0.381***	-0.239***	-0.414***
					(0.0000)	(0.0000)	(0.0010)	(0.0000)
High Post-1500 CE Yield Ch. (Anc.)*Jul					-0.227***	-0.440***	-0.251***	-0.465***
					(0.0010)	(0.0000)	(0.0020)	(0.0000)
High Post-1500 CE Yield Ch. (Anc.)*Aug					-0.195***	-0.442***	-0.226***	-0.469***
					(0.0000)	(0.0000)	(0.0000)	(0.0000)
High Post-1500 CE Yield Ch. (Anc.)*Sep					-0.174***	-0.467***	-0.188***	-0.477***
					(0.0010)	(0.0010)	(0.0030)	(0.0010)
High Post-1500 CE Yield Ch. (Anc.)*Oct					-0.155***	-0.490***	-0.167***	-0.498***
					(0.0030)	(0.0000)	(0.0050)	(0.0000)
Observations	6,515	6,515	6,515	6,515	6,515	6,515	6,515	6,515
R-squared	0.276	0.291	0.276	0.292	0.261	0.273	0.262	0.275
Number of counties	769	769	769	769	769	769	769	769
State*Trends	No	Yes	No	Yes	No	Yes	No	Yes
Geographic Controls	Yes	Yes	Yes	Yes	Yes	Yes	Yes	Yes
Mobility Neighboring County	Yes	Yes	Yes	Yes	Yes	Yes	Yes	Yes

Notes:"()" reports the p-values of the wild cluster bootstrap method using the post-estimation command boottest. Stars $^{***(**)[*]}$ indicate significance at the $0.01(0.05)[0.1]$ level. This table shows the effect of the ancestry-adjusted high pre-1500 CE potential crop yield (where ancestry-adjusted high pre-1500 CE potential crop yield is a dummy variable that equals 1 if the ancestry-adjusted potential crop yield is above the sample median) and the ancestry-adjusted high post-1500 potential crop yield change during the Columbian Exchange (where ancestry-adjusted high potential yield change is a dummy variable that equals 1 if the potential crop yield change is above the sample median) on monthly total county visitors and total county visits to fitness centers obtained from SafeGraph's Patterns data. The outcome variables in columns (1), (2), (5), and (6) are the total number of visitors from each county visiting fitness centers. The outcome variables in columns (3), (4), (7), and (8) are the total number of visits by county residents to fitness centers, which are calculated by multiplying the average visits per visitor to fitness centers in each county with the total visitors by county to fitness centers. The reported effects are relative to the baseline variable in the month of February 2020, based on Equation 2. All columns control for COVID-19 cases in neighboring counties, monthly mobility proxies in neighboring counties, temperature, and precipitation. Columns (2), (4), (6) and (8) control for state-specific time trends. Predicted ancestral compositions in each county using the instruments from Burchardi, Chaney and Hassan (2019) and the post-1500 World Migration Matrix of Putterman and Weil (2010) were used to adjust crop yield measures. All variables are normalized by subtracting their mean and dividing by their standard deviation. Therefore, all coefficients are comparable and estimate the effect of a one standard deviation increase in the independent variable. Standard errors are clustered at the commuting zone level.

TABLE B.19. DYNAMIC CAUSAL EFFECT OF LONG-TERM ORIENTATION ON VISITORS/VISITS TO HOBBY CENTERS AND GAMING STORES

	MOBILITY BEHAVIOR OF COUNTY RESIDENTS (FEB-OCTOBER 2020)							
**HOBBY CENTERS AND GAMING STORES**	**(1)** Visitors	**(2)** Visitors	**(3)** Visits	**(4)** Visits	**(5)** Visitors	**(6)** Visitors	**(7)** Visits	**(8)** Visits
High Pre-1500 CE Yield (Anc.)*Mar	-0.103***	-0.0771***	-0.109***	-0.0906***				
	(0.0000)	(0.0050)	(0.0000)	(0.0030)				
High Pre-1500 CE Yield (Anc.)*Apr	-0.245***	-0.195***	-0.246***	-0.212***				
	(0.0000)	(0.0020)	(0.0000)	(0.0010)				
High Pre-1500 CE Yield (Anc.)*May	-0.164***	-0.0865**	-0.173***	-0.118***				
	(0.0010)	(0.0310)	(0.0000)	(0.0100)				
High Pre-1500 CE Yield (Anc.)*Jun	-0.0938***	0.00149	-0.106***	-0.0411				
	(0.0000)	(0.9489)	(0.0000)	(0.1862)				
High Pre-1500 CE Yield (Anc.)*Jul	-0.0981***	0.0175	-0.110***	-0.0320				
	(0.0000)	(0.5516)	(0.0000)	(0.3293)				
High Pre-1500 CE Yield (Anc.)*Aug	-0.0697***	0.0682*	-0.0842***	0.00934				
	(0.0000)	(0.0631)	(0.0000)	(0.7908)				
High Pre-1500 CE Yield (Anc.)*Sep	-0.0996***	0.0658	-0.111***	0.00151				
	(0.0000)	(0.1011)	(0.0000)	(0.9620)				
High Pre-1500 CE Yield (Anc.)*Oct	-0.0826***	0.105**	-0.0958***	0.0316				
	(0.0000)	(0.0180)	(0.0000)	(0.4515)				
High Post-1500 CE Yield Ch. (Anc.)*Mar					-0.0256	-0.0100	-0.0304	-0.0204
					(0.3173)	(0.6917)	(0.2533)	(0.4565)
High Post-1500 CE Yield Ch. (Anc.)*Apr					-0.0463	-0.0158	-0.0499	-0.0306
					(0.4444)	(0.7848)	(0.4014)	(0.6016)
High Post-1500 CE Yield Ch. (Anc.)*May					-0.0772**	-0.0325	-0.0799**	-0.0520
					(0.013)	(0.3343)	(0.0140)	(0.1502)
High Post-1500 CE Yield Ch. (Anc.)*Jun					-0.0478*	0.00560	-0.0570**	-0.0256
					(0.0641)	(0.8619)	(0.0350)	(0.4274)
High Post-1500 CE Yield Ch. (Anc.)*Jul					-0.0624***	0.000379	-0.0722***	-0.0366
					(0.0050)	(0.9880)	(0.0020)	(0.3093)
High Post-1500 CE Yield Ch. (Anc.)*Aug					-0.0473**	0.0311	-0.0561**	-0.00951
					(0.0200)	(0.4034)	(0.0180)	(0.7938)
High Post-1500 CE Yield Ch. (Anc.)*Sep					-0.0562***	0.0406	-0.0665***	-0.00736
					(0.0100)	(0.3443)	(0.0040)	(0.8839)
High Post-1500 CE Yield Ch. (Anc.)*Oct					-0.0568***	0.0530	-0.0669***	-0.000384
					(0.0060)	(0.3063)	(0.0040)	(0.9900)
Observations	6,515	6,515	6,515	6,515	6,515	6,515	6,515	6,515
R-squared	0.355	0.364	0.347	0.353	0.347	0.353	0.338	0.343
Number of counties	769	769	769	769	769	769	769	769
State*Trends	No	Yes	No	Yes	No	Yes	No	Yes
Geographic Controls	Yes	Yes	Yes	Yes	Yes	Yes	Yes	Yes
Mobility Neighboring County	Yes	Yes	Yes	Yes	Yes	Yes	Yes	Yes

Notes:"()" reports the p-values of the wild cluster bootstrap method using the post-estimation command boottest. Stars $^{***(**)[*]}$ indicate significance at the 0.01(0.05)[0.1] level. This table presents the effect of the ancestry-adjusted high pre-1500 CE potential crop yield (where ancestry-adjusted high pre-1500 CE potential crop yield is a dummy variable that equals 1 if the ancestry-adjusted potential crop yield is above the sample median) and the ancestry-adjusted high post-1500 potential crop yield change during the Columbian Exchange (where high ancestry-adjusted potential yield change is a dummy variable that equals 1 if the potential crop yield change is above the sample median) on monthly total county visitors and total county visits to hobby centers and gaming stores obtained from SafeGraph's Patterns data. The outcome variables in columns (1), (2), (5), and (6) are the total visitors from each county visiting hobby centers and gaming stores. The outcome variables in columns (3), (4), (7), and (8) are the total visits by county residents to hobby centers and gaming stores, which are calculated by multiplying the average visits per visitor to hobby centers and gaming stores in each county with the total visitors by county to hobby centers and gaming stores. The reported effects are relative to the baseline variable in the month of February 2020, based on Equation 2. All columns control for COVID-19 cases in neighboring counties, monthly mobility proxies in neighboring counties, temperature, and precipitation. Columns (2), (4), (6), and (8) control for state-specific time trends. Predicted ancestral compositions in each county using the instruments from Burchardi, Chaney and Hassan (2019) and the post-1500 World Migration Matrix of Putterman and Weil (2010) were used to adjust crop yield measures. All variables are normalized by subtracting their mean and dividing by their standard deviation. Therefore, all coefficients are comparable and estimate the effect of a one standard deviation increase in the independent variable. Standard errors are clustered at the commuting zone level.

Table B.20. DYNAMIC CAUSAL EFFECT OF LONG-TERM ORIENTATION ON MOBILITY: GOOGLE MOBILITY REPORT

MOBILITY BEHAVIOR OF COUNTY RESIDENTS (FEB-OCTOBER 2020)				
**PANEL A GOOGLE MOBILITY**	**(1)**<br>Away from Home	**(2)**<br>Visits Recreation	**(3)**<br>Away from Home	**(4)**<br>Visits Recreation
High Pre-1500 CE Yield (Anc.)*Mar	-0.525***	-2.175***		
	(0.0050)	(0.0020)		
High Pre-1500 CE Yield (Anc.)*Apr	-0.584***	-3.838***		
	(0.0010)	(0.0000)		
High Pre-1500 CE Yield (Anc.)*May	-0.695***	-5.324***		
	(0.0000)	(0.0000)		
High Pre-1500 CE Yield (Anc.)*Jun	-0.676***	-4.904***		
	(0.0000)	(0.0000)		
High Pre-1500 CE Yield (Anc.)*Jul	-0.746***	-5.596***		
	(0.0000)	(0.0000)		
High Pre-1500 CE Yield (Anc.)*Aug	-0.793***	-6.193***		
	(0.0000)	(0.0000)		
High Pre-1500 CE Yield (Anc.)*Sep	-0.908***	-7.722***		
	(0.0000)	(0.0000)		
High Pre-1500 CE Yield (Anc.)*Oct	-0.867***	-9.022***		
	(0.0000)	(0.0000)		
High Post-1500 CE Yield Ch. (Anc.)*Mar			-0.563***	-1.429**
			(0.0030)	(0.0220)
High Post-1500 CE Yield Ch. (Anc.)*Apr			-0.892***	-2.010**
			(0.0000)	(0.0250)
High Post-1500 CE Yield Ch. (Anc.)*May			-0.675***	-1.868**
			(0.0000)	(0.0420)
High Post-1500 CE Yield Ch. (Anc.)*Jun			-0.550***	-0.472
			(0.0000)	(0.6426)
High Post-1500 CE Yield Ch. (Anc.)*Jul			-0.626***	-1.020
			(0.0000)	(0.3213)
High Post-1500 CE Yield Ch. (Anc.)*Aug			-0.626***	-0.599
			(0.0010)	(0.5716)
High Post-1500 CE Yield Ch. (Anc.)*Sep			-0.620***	-1.013
			(0.0000)	(0.3253)
High Post-1500 CE Yield Ch. (Anc.)*Oct			-0.689***	-3.147**
			(0.0010)	(0.0150)
Observations	9,966	9,966	9,966	9,966
R-squared	0.931	0.763	0.932	0.762
Number of counties	1,277	1,277	1,277	1,277
State*Trends	Yes	Yes	Yes	Yes
Geographic Controls	Yes	Yes	Yes	Yes
Mobility and Cases Neighboring County	Yes	Yes	Yes	Yes

Notes:"()" reports the p-values of the wild cluster bootstrap method using the post-estimation command boottest. Stars $^{***(**)[*]}$ indicate significance at the 0.01(0.05)[0.1] level. This table presents the effect of the ancestry-adjusted high pre-1500 CE potential crop yield (where ancestry-adjusted high pre-1500 CE potential crop yield is a dummy variable that equals 1 if the ancestry-adjusted potential crop yield is above the sample median) and the ancestry-adjusted high post-1500 CE potential crop yield change during the Columbian Exchange (where ancestry-adjusted high post-1500 potential yield change is a dummy variable that equals 1 if the potential crop yield change is above the sample median) on monthly county-level data on the percentage change in hours spent away from residential places and the percentage change in visits to recreation centers such as restaurants, cafes, shopping centers, theme parks, museums, libraries, and movie theaters. The outcome variables are measured relative to the pre-crisis level (i.e., between January 3, 2020 and February 6, 2020). The monthly data are from 15th of each month to the 14th of the following month. The reported effects are relative to the outcome variable measured in the month of February 2020, based on Equation 2. All columns control for the average COVID-19 case prevalence in neighboring counties, the percentage change in hours spent away from the home averaged across neighboring counties, temperature, precipitation, and state-specific time trends. Predicted ancestral compositions in each county using the instruments from Burchardi, Chaney and Hassan (2019) and the post-1500 World Migration Matrix of Putterman and Weil (2010) were used to adjust crop yield measures. All variables are normalized by subtracting their mean and dividing by their standard deviation. Therefore, all coefficients are comparable and estimate the effect of a one standard deviation increase in the independent variable. Standard errors are clustered at the commuting zone level.

Table B.21. DYNAMIC CAUSAL EFFECT OF LONG-TERM ORIENTATION

	JANUARY-OCTOBER 2020				
	**(1)**	**(2)**	**(3)**	**(4)**	**(5)**
Total Visitors	Hobby Centers	Clothing Stores	Fitness Centers	Restaurants	Movie Theaters
High Pre-1500 CE (Anc.)*Jan	0.0404	0.0464	0.0547	0.0441	0.155
	[0.0144]***	[0.0155]***	[0.0162]***	[0.0147]***	[0.0530]***
	{0.0070}***	{0.0040}***	{0.0000}***	{0.0040}***	{0.0040}***
High Pre-1500 CE (Anc.)*Mar	-0.0959	-0.163	-0.170	-0.113	-0.238
	[0.0272]***	[0.0404]***	[0.0362]***	[0.0283]***	[0.0490]***
	{0.0000}***	{0.0000}***	{0.0000}***	{0.0000}***	{0.0000}***
High Pre-1500 CE (Anc.)*Apr	-0.224	-0.320	-0.387	-0.231	-0.359
	[0.0602]***	[0.0775]***	[0.0856]***	[0.0582]***	[0.0964]***
	{0.0000}***	{0.0000}***	{0.0000}***	{0.0000}***	{0.0000}***
High Pre-1500 CE (Anc.)*May	-0.146	-0.254	-0.323	-0.169	-0.363
	[0.0392]***	[0.0630]***	[0.0732]***	[0.0440]***	[0.0958]***
	{0.0000}***	{0.0000}***	{0.0000}***	{0.0000}***	{0.0000}***
High Pre-1500 CE (Anc.)*June	-0.0877	-0.164	-0.284	-0.141	-0.405
	[0.0249]***	[0.0440]***	[0.0624]***	[0.0372]***	[0.0973]***
	{0.0010}***	{0.0000}***	{0.0000}***	{0.0000}***	{0.0010}***
High Pre-1500 CE (Anc.)*Jul	-0.0945	-0.154	-0.281	-0.140	-0.431
	[0.0268]***	[0.0454]***	[0.0667]***	[0.0401]***	[0.104]***
	{0.0000}***	{0.0000}***	{0.0000}***	{0.0000}***	{0.0000}***
High Pre-1500 CE (Anc.)*Aug	-0.0690	-0.135	-0.260	-0.122	-0.413
	[0.0210]***	[0.0345]***	[0.0610]***	[0.0337]***	[0.0997]***
	{0.0000}***	{0.0000}***	{0.0000}***	{0.0000}***	{0.0000}***
High Pre-1500 CE (Anc.)*Sep	-0.0981	-0.161	-0.257	-0.147	-0.402
	[0.0240]***	[0.0414]***	[0.0640]***	[0.0396]***	[0.0914]***
	{0.0000}***	{0.0000}***	{0.0000}***	{0.0000}***	{0.0000}***
High Pre-1500 CE (Anc.)*Oct	-0.0831	-0.143	-0.242	-0.134	-0.357
	[0.0239]***	[0.0428]***	[0.0632]***	[0.0403]***	[0.0895]***
	{0.0000}***	{0.0000}***	{0.0000}***	{0.0000}***	{0.0000}***
Observations	7,261	7,261	7,261	7,261	7,261
R-squared	0.328	0.227	0.273	0.238	0.275
Number of counties	771	771	771	771	771
High Post-1500 CE Yield Ch. (Anc.)*Jan	0.0117	0.0198	0.0309	0.0211	0.0160
	[0.0173]	[0.0155]	[0.0154]**	[0.0141]	[0.0540]
	{0.6036}	{0.2643 }	{0.0911}*	{0.1912}	{0.8018}
High Post-1500 CE Yield Ch. (Anc.)*Mar	-0.0221	-0.0669	-0.0783	-0.0403	-0.115
	[0.0239]	[0.0334]**	[0.0322]**	[0.0244]*	[0.0559]**
	{0.3283}	{0.0541}*	{0.0270}**	{0.1041}	{0.0490}**
High Post-1500 CE Yield Ch. (Anc.)*Apr	-0.0343	-0.0977	-0.136	-0.0656	-0.101
	[0.0562]	[0.0672]	[0.0706]*	[0.0504]	[0.101]
	{0.5315 }	{0.1231}	{0.0541}*	{0.1832}	{0.3443}
High Post-1500 CE Yield Ch. (Anc.)*May	-0.0664	-0.0989	-0.126	-0.0586	-0.134
	[0.0301]**	[0.0512]*	[0.0598]**	[0.0368]	[0.100]
	{0.0350}**	{0.0501}*	{0.0280}**	{0.0941}*	{0.2022}
High Post-1500 CE Yield Ch. (Anc.)*Jun	-0.0529	-0.107	-0.188	-0.0898	-0.246
	[0.0232]**	[0.0422]**	[0.0553]***	[0.0342]***	[0.101]**
	{0.0150}**	{0.0090}***	{0.0010}***	{0.0080}***	{0.0170}**
High Post-1500 CE Yield Ch. (Anc.)*Jul	-0.0751	-0.127	-0.217	-0.109	-0.313
	[0.0268]***	[0.0494]**	[0.0668]***	[0.0416]***	[0.115]***
	{0.0010}***	{0.0010}**	{0.0000}***	{0.0030}***	{0.0040}***
High Post-1500 CE Yield Ch. (Anc.)*Aug	-0.0524	-0.0973	-0.181	-0.0806	-0.268
	[0.0220]**	[0.0391]**	[0.0578]***	[0.0341]**	[0.105]**
	{0.0120}**	{0.0060}***	{0.0010}***	{0.0080}***	{0.0070}***
High Post-1500 CE Yield Ch. (Anc.)*Sep	-0.0603	-0.0922	-0.159	-0.0825	-0.251
	[0.0224]***	[0.0396]**	[0.0543]***	[0.0347]**	[0.0923]***
	{0.0030}***	{0.0080}***	{0.0000}***	{0.0140}**	{0.0040}***
High Post-1500 CE Yield Ch. (Anc.)*Oct	-0.0583	-0.0834	-0.139	-0.0761	-0.207
	[0.0207]***	[0.0373]**	[0.0494]***	[0.0320]**	[0.0845]**
	{0.0040}***	{0.0140}**	{0.0030}***	{0.0120}**	{0.0210}**
Observations	7,261	7,261	7,261	7,261	7,261
R-squared	0.319	0.216	0.259	0.227	0.265
Number of counties	771	771	771	771	771
Geo Controls	Y	Y	Y	Y	Y
Neighboring COVID-19 cases	Y	Y	Y	Y	Y
Neighboring Mobility	Y	Y	Y	Y	Y

Notes: Bootstrap standard errors using vce(bootstrap) command in STATA are reported in "[]"; *p*-values of the wild cluster bootstrap method using the post-estimation command boottest are reported in "{}." Stars $^{***(**)[*]}$ indicate significance at the 0.01(0.05)[0.1] level. This table presents the effect of the ancestry-adjusted high pre-1500 CE potential crop yield (where ancestry-adjusted high pre-1500 CE potential crop yield is a dummy variable that equals 1 if the ancestry-adjusted potential crop yield is above the sample median) and the ancestry-adjusted high post-1500 CE potential crop yield change during the Columbian Exchange (where ancestry-adjusted high post-1500 potential yield change is a dummy variable that equals 1 if the potential crop yield change is above the sample median) on the total number of county visitors to hobby centers, restaurants, clothing stores, fitness centers, and movie theaters. All columns control for the average COVID-19 case prevalence in neighboring counties, monthly mobility in neighboring counties, temperature, and precipitation. Predicted ancestral compositions in each county using the instruments from Burchardi, Chaney and Hassan (2019) and the post-1500 World Migration Matrix of Putterman and Weil (2010) were used to adjust crop yield measures. All variables are normalized by subtracting their mean and dividing by their standard deviation. Therefore, all coefficients are comparable and estimate the effect of a one standard deviation increase in the independent variable. Standard errors are clustered at the commuting zone level.

Table B.22. SENSITIVITY TO DEVIATIONS FROM PARALLEL TRENDS: VISITORS TO FITNESS CENTERS

	Lower Bound	Upper Bound	Method	DeltaSDPB	M
March					
	-0.20	-0.08	C-F	DeltaSDPB	0.00
	-0.28	-0.10	C-F	DeltaSDPB	0.10
	-0.38	-0.10	C-F	DeltaSDPB	0.20
	-0.48	-0.10	C-F	DeltaSDPB	0.30
April					
	-0.47	-0.18	C-F	DeltaSDPB	0.00
	-0.72	-0.22	C-F	DeltaSDPB	0.10
	-1.01	-0.23	C-F	DeltaSDPB	0.20
	-1.31	-0.23	C-F	DeltaSDPB	0.30
May					
	-0.36	-0.15	C-F	DeltaSDPB	0.00
	-0.88	-0.18	C-F	DeltaSDPB	0.10
	-1.48	-0.19	C-F	DeltaSDPB	0.20
	-2.15	-0.19	C-F	DeltaSDPB	0.30
June					
	-0.24	-0.13	C-F	DeltaSDPB	0.00
	-1.18	-0.16	C-F	DeltaSDPB	0.10
	-2.18	2.11	C-F	DeltaSDPB	0.20
	-3.17	3.11	C-F	DeltaSDPB	0.30
July					
	-0.23	-0.12	C-F	DeltaSDPB	0.00
	-1.64	-0.15	C-F	DeltaSDPB	0.10
	-3.13	-0.16	C-F	DeltaSDPB	0.20
	-4.64	-0.16	C-F	DeltaSDPB	0.30
August					
	-0.15	-0.12	C-F	DeltaSDPB	0.00
	-2.16	-0.15	C-F	DeltaSDPB	0.10
	-4.26	-0.16	C-F	DeltaSDPB	0.20
	-6.36	-0.16	C-F	DeltaSDPB	0.30
September					
	-0.15	-0.10	C-F	DeltaSDPB	0.00
	-2.83	-0.13	C-F	DeltaSDPB	0.10
	-5.64	-0.15	C-F	DeltaSDPB	0.20
	-8.42	8.78	C-F	DeltaSDPB	0.30
October					
	-0.13	-0.08	C-F	DeltaSDPB	0.00
	-3.70	-0.12	C-F	DeltaSDPB	0.10
	-7.30	-0.13	C-F	DeltaSDPB	0.20
	-10.90	-0.13	C-F	DeltaSDPB	0.30

This table constructs confidence sets for post-period effects of culturally embodied LTO traits that are robust to violations of the parallel trends assumption following Rambachan and Roth (2019). M denotes the upper bound of the change in the slope of the underlying trend in each consecutive period. The choice of M=0 restricts violations of parallel trends to be linear. The choice of $M > 0$ allows violations of parallel trends to be non-linear. DeltaSDPB indicates the analysis that combines deviations from parallel trends with positive bias post-periods.

Table B.23. SENSITIVITY TO DEVIATIONS FROM PARALLEL TRENDS: VISITORS TO HOBBY CENTERS

	Lower Bound	Upper Bound	Method	DeltaSDPB	M
March					
	-0.12	-0.03	C-F	DeltaSDPB	0.00
	-0.20	-0.04	C-F	DeltaSDPB	0.10
	-0.30	-0.04	C-F	DeltaSDPB	0.20
	-0.40	0.32	C-F	DeltaSDPB	0.30
April					
	-0.27	-0.09	C-F	DeltaSDPB	0.00
	-0.53	-0.12	C-F	DeltaSDPB	0.10
	-0.87	-0.12	C-F	DeltaSDPB	0.20
	-1.13	-0.12	C-F	DeltaSDPB	0.30
May					
	-0.16	-0.05	C-F	DeltaSDPB	0.00
	-0.70	-0.07	C-F	DeltaSDPB	0.10
	-1.35	-0.07	C-F	DeltaSDPB	0.20
	-1.95	-0.07	C-F	DeltaSDPB	0.30
June					
	-0.07	-0.02	C-F	DeltaSDPB	0.00
	-1.06	-0.04	C-F	DeltaSDPB	0.10
	-2.01	-0.04	C-F	DeltaSDPB	0.20
	-3.01	3.21	C-F	DeltaSDPB	0.30
July					
	-0.08	-0.02	C-F	DeltaSDPB	0.00
	-1.56	-0.04	C-F	DeltaSDPB	0.10
	-3.00	-0.04	C-F	DeltaSDPB	0.20
	-4.56	-0.05	C-F	DeltaSDPB	0.30
August					
	-0.03	0.38	C-F	DeltaSDPB	0.00
	-2.05	2.47	C-F	DeltaSDPB	0.10
	-4.15	-0.03	C-F	DeltaSDPB	0.20
	-6.24	-0.04	C-F	DeltaSDPB	0.30
September					
	-0.07	0.44	C-F	DeltaSDPB	0.00
	-2.76	-0.06	C-F	DeltaSDPB	0.10
	-5.56	-0.05	C-F	DeltaSDPB	0.20
	-8.37	8.82	C-F	DeltaSDPB	0.30
October					
	-0.06	-0.02	C-F	DeltaSDPB	0.00
	-3.53	-0.04	C-F	DeltaSDPB	0.10
	-7.14	-0.04	C-F	DeltaSDPB	0.20
	-10.73	-0.04	C-F	DeltaSDPB	0.30

This table constructs confidence sets for post-period effects of culturally embodied LTO traits that are robust to violations of the parallel trends assumption following Rambachan and Roth (2019). M denotes the upper bound of the change in the slope of the underlying trend in each consecutive period. The choice of M=0 restricts violations of parallel trends to be linear. The choice of $M > 0$ allows violations of parallel trends to be non-linear. DeltaSDPB indicates the analysis that combines deviations from parallel trends with positive bias post-periods.

Table B.24. SENSITIVITY TO DEVIATIONS FROM PARALLEL TRENDS: VISITORS TO CLOTHING STORES

	Lower Bound	Upper Bound	Method	DeltaSDPB	M
March					
	-0.20	-0.07	C-F	DeltaSDPB	0.00
	-0.28	-0.08	C-F	DeltaSDPB	0.10
	-0.38	-0.09	C-F	DeltaSDPB	0.20
	-0.51	-0.09	C-F	DeltaSDPB	0.30
April					
	-0.38	-0.14	C-F	DeltaSDPB	0.00
	-0.64	-0.17	C-F	DeltaSDPB	0.10
	-0.93	-0.18	C-F	DeltaSDPB	0.20
	-1.29	-0.18	C-F	DeltaSDPB	0.30
May					
	-0.26	-0.11	C-F	DeltaSDPB	0.00
	-0.85	-0.13	C-F	DeltaSDPB	0.10
	-1.45	-0.14	C-F	DeltaSDPB	0.20
	-2.00	-0.14	C-F	DeltaSDPB	0.30
June					
	-0.11	-0.06	C-F	DeltaSDPB	0.00
	-1.05	-0.08	C-F	DeltaSDPB	0.10
	-2.10	-0.08	C-F	DeltaSDPB	0.20
	-3.05	3.14	C-F	DeltaSDPB	0.30
July					
	-0.09	-0.05	C-F	DeltaSDPB	0.00
	-1.52	-0.07	C-F	DeltaSDPB	0.10
	-3.02	-0.07	C-F	DeltaSDPB	0.20
	-4.58	-0.08	C-F	DeltaSDPB	0.30
August					
	0.34	0.34	C-F	DeltaSDPB	0.00
	-2.07	-0.07	C-F	DeltaSDPB	0.10
	-4.17	4.53	C-F	DeltaSDPB	0.20
	-6.34	6.63	C-F	DeltaSDPB	0.30
September					
	0.40	0.40	C-F	DeltaSDPB	0.00
	-2.77	-0.08	C-F	DeltaSDPB	0.10
	-5.66	5.98	C-F	DeltaSDPB	0.20
	-8.37	8.78	C-F	DeltaSDPB	0.30
October					
	-0.06	0.51	C-F	DeltaSDPB	0.00
	-3.54	-0.06	C-F	DeltaSDPB	0.10
	-7.13	-0.06	C-F	DeltaSDPB	0.20
	-10.73	-0.07	C-F	DeltaSDPB	0.30

This table constructs confidence sets for post-period effects of culturally embodied LTO traits that are robust to violations of the parallel trends assumption following Rambachan and Roth (2019). M denotes the upper bound of the change in the slope of the underlying trend in each consecutive period. The choice of M=0 restricts violations of parallel trends to be linear. The choice of $M > 0$ allows violations of parallel trends to be non-linear. DeltaSDPB indicates the analysis that combines deviations from parallel trends with positive bias post-periods.

Table B.25. SENSITIVITY TO DEVIATIONS FROM PARALLEL TRENDS: VISITORS TO RESTAURANTS

	Lower Bound	Upper Bound	Method	DeltaSDPB	M
March					
	-0.13	-0.04	C-F	DeltaSDPB	0.00
	-0.21	-0.06	C-F	DeltaSDPB	0.10
	-0.31	0.20	C-F	DeltaSDPB	0.20
	-0.43	-0.06	C-F	DeltaSDPB	0.30
April					
	-0.26	-0.09	C-F	DeltaSDPB	0.00
	-0.52	-0.12	C-F	DeltaSDPB	0.10
	-0.86	0.57	C-F	DeltaSDPB	0.20
	-1.12	0.87	C-F	DeltaSDPB	0.30
May					
	-0.14	-0.06	C-F	DeltaSDPB	0.00
	-0.70	-0.08	C-F	DeltaSDPB	0.10
	-1.30	-0.09	C-F	DeltaSDPB	0.20
	-1.90	1.86	C-F	DeltaSDPB	0.30
June					
	-0.08	0.15	C-F	DeltaSDPB	0.00
	-1.03	-0.07	C-F	DeltaSDPB	0.10
	-2.07	-0.07	C-F	DeltaSDPB	0.20
	-3.07	-0.07	C-F	DeltaSDPB	0.30
July					
	-0.07	0.23	C-F	DeltaSDPB	0.00
	-1.56	1.72	C-F	DeltaSDPB	0.10
	-3.00	-0.07	C-F	DeltaSDPB	0.20
	-4.50	4.72	C-F	DeltaSDPB	0.30
August					
	0.31	0.32	C-F	DeltaSDPB	0.00
	-2.06	-0.06	C-F	DeltaSDPB	0.10
	-4.16	4.51	C-F	DeltaSDPB	0.20
	-6.26	6.61	C-F	DeltaSDPB	0.30
September					
	0.37	0.38	C-F	DeltaSDPB	0.00
	-2.76	3.16	C-F	DeltaSDPB	0.10
	-5.56	5.96	C-F	DeltaSDPB	0.20
	-8.44	8.76	C-F	DeltaSDPB	0.30
October					
	0.48	0.48	C-F	DeltaSDPB	0.00
	-3.53	4.06	C-F	DeltaSDPB	0.10
	-7.14	7.66	C-F	DeltaSDPB	0.20
	-10.71	11.26	C-F	DeltaSDPB	0.30

This table constructs confidence sets for post-period effects of culturally embodied LTO traits that are robust to violations of the parallel trends assumption following Rambachan and Roth (2019). M denotes the upper bound of the change in the slope of the underlying trend in each consecutive period. The choice of M=0 restricts violations of parallel trends to be linear. The choice of $M > 0$ allows violations of parallel trends to be non-linear. DeltaSDPB indicates the analysis that combines deviations from parallel trends with positive bias post-periods.

Table B.26. SENSITIVITY TO DEVIATIONS FROM PARALLEL TRENDS: VISITORS TO MOVIE THEATERS (EXCEPT DRIVE-INS)

	**Lower Bound**	**Upper Bound**	**Method**	**DeltaSDPB**	**M**
March					
	-0.18	-0.10	C-F	DeltaSDPB	0.00
	-0.26	-0.14	C-F	DeltaSDPB	0.10
	-0.35	0.22	C-F	DeltaSDPB	0.20
	-0.45	0.32	C-F	DeltaSDPB	0.30
April					
	-0.19	-0.11	C-F	DeltaSDPB	0.00
	-0.47	0.39	C-F	DeltaSDPB	0.10
	-0.74	-0.19	C-F	DeltaSDPB	0.20
	-1.04	-0.19	C-F	DeltaSDPB	0.30
May					
	-0.18	0.38	C-F	DeltaSDPB	0.00
	-0.66	0.96	C-F	DeltaSDPB	0.10
	-1.36	1.56	C-F	DeltaSDPB	0.20
	-1.86	-0.20	C-F	DeltaSDPB	0.30
June					
	0.59	0.61	C-F	DeltaSDPB	0.00
	-1.07	1.58	C-F	DeltaSDPB	0.10
	-2.02	2.57	C-F	DeltaSDPB	0.20
	-3.02	3.58	C-F	DeltaSDPB	0.30
July					
	0.86	0.89	C-F	DeltaSDPB	0.00
	-1.57	2.34	C-F	DeltaSDPB	0.10
	-2.98	3.84	C-F	DeltaSDPB	0.20
	-4.47	5.34	C-F	DeltaSDPB	0.30
August					
	1.16	1.19	C-F	DeltaSDPB	0.00
	-2.15	3.23	C-F	DeltaSDPB	0.10
	-4.07	-0.25	C-F	DeltaSDPB	0.20
	-6.17	-0.25	C-F	DeltaSDPB	0.30
September					
	1.50	1.53	C-F	DeltaSDPB	0.00
	-2.86	4.26	C-F	DeltaSDPB	0.10
	-5.40	7.06	C-F	DeltaSDPB	0.20
	-8.50	-0.25	C-F	DeltaSDPB	0.30
October					
	1.86	1.89	C-F	DeltaSDPB	0.00
	-3.64	5.41	C-F	DeltaSDPB	0.10
	-6.90	9.01	C-F	DeltaSDPB	0.20
	-10.49	12.61	C-F	DeltaSDPB	0.30

This table constructs confidence sets for post-period effects of culturally embodied LTO traits that are robust to violations of the parallel trends assumption following Rambachan and Roth (2019). M denotes the upper bound of the change in the slope of the underlying trend in each consecutive period. The choice of M=0 restricts violations of parallel trends to be linear. The choice of $M > 0$ allows violations of parallel trends to be non-linear. DeltaSDPB indicates the analysis that combines deviations from parallel trends with positive bias post-periods.

Table B.27. EFFECTS OF LONG-TERM ORIENTATION TRAITS ON COVID-19 SEVERITY AT THE COMMUTING ZONE LEVEL

	(1)<br>Cases	(2)<br>Deaths	(3)<br>Excess (>=45)	(4)<br>Excess (>=55)	(5)<br>Excess (>=65)	(6)<br>Hospitalized	(7)<br>Inpatient Bed Used
Pre-1500 CE Yield (Anc.)	-9.801*	-5.094	0.824	-7.757	-8.278	-3.057	-5.906**
	(0.0941)	(0.412)	(0.892)	(0.208)	(0.246)	(0.715)	(0.045)
Post-1500 CE Yield Ch. (Anc.)	-1.705***	-1.242***	-1.184***	-1.458***	-1.463***	-1.277**	-0.163
	(0.000)	(0.000)	(0.002)	(0.002)	(0.000)	(0.018)	(0.360)
Observations	673	673	452	579	624	669	668
R-squared	0.923	0.904	0.928	0.939	0.938	0.698	0.868
Geographic	Y	Y	Y	Y	Y	Y	Y
Socio-Economic, Pol., Health	Y	Y	Y	Y	Y	Y	Y
State Dummies	Y	Y	Y	Y	Y	Y	Y

Notes: "()" reports the p-values of the wild cluster bootstrap method using the post-estimation command boottest. Stars $^{***(**)[*]}$ indicate significance at the 0.01(0.05)[0.1] level. This table reports the effect of the ancestry-adjusted pre-1500 CE crop yield and ancestry-adjusted post-1500 crop yield change on average COVID-19 case prevalence, death prevalence, and excess deaths in age groups 45 and older, 55 and older, and 65 and older; and total COVID-19 hospitalizations per tests per 100,000 and average inpatient bed utilization rates at the level of commuting zone. All columns include state dummies; geographic factors that include average roughness of terrain, precipitation, temperature, and elevation; average number of large central or large fringe metro counties and medium metro and small metro counties; mean income; proportion of males; population density; proportion of population from a Black or African American, Native American, White, and Hispanic or Latino background; proportion of population using public transport; proportion of family and non-family households living in two or more unit structures; proportion of family and non-family households with three or more members; proportion of population in age groups younger than 19, 19–34, and 35–64 (with 65 and older as the omitted group); proportion working from home; proportion of population with an education level higher than or equal to the higher secondary level; healthcare coverage measured as the proportion of the population with two or more health insurance policies in the age groups younger than 19, 19–34, 35–64, and 65 and older, and the proportion of the population older than age 65 without any health insurance; distance to an airport with direct international flights to high-severity countries; Gini index and proportion of population below the poverty line; Social Capital Index; percentage of adult population with obesity; percentage of population who smoke as adults; 30-day risk-adjusted mortality rate; heart disease death rate; percentage of population diagnosed with diabetes among adults older than 20 years of age; contribution to the percentage change in GDP by private-services-providing industries, private-goods-providing industries, and government enterprises, and government enterprises; percentage change in annual average employment for a given year; and proportion of votes for Democrats in the 2016 US presidential election. Columns (3), (4), (5), (7) controls for average COVID-19 tests per 100,000 at the commuting zone level. Additionally, columns (6) and (7) add controls measuring pre-crisis hospital capacity (includes proportion of full-time staff, beds available, hospitals that were short term, hospitals operated by the Catholic Church, critical access hospitals, rural referral centers, and sole community providers; proportion with DNV Healthcare accreditation; and proportion of hospitals with a Center for Improvement in Healthcare Quality accreditation) and types of hospital reporting (comprising the proportion of critical access hospitals reporting, children's hospitals reporting, and short-term hospitals). All variables are normalized by subtracting their mean and dividing by their standard deviation. Therefore, all coefficients are comparable and estimate the effect of a one standard deviation increase in the independent variable.

Table B.28. EFFECT OF LONG-TERM ORIENTATION TRAITS ON MOBILITY PROXIES AT THE COMMUTING ZONE LEVEL: VISITORS

TOTAL VISITORS FROM COUNTY TO POI								
**PANEL A: HOBBY CENTERS**	**(1)**	**(2)**	**(3)**	**(4)**	**(5)**	**(6)**	**(7)**	**(8)**
	March	April	May	June	July	August	September	October
Pre-1500 CE Yield (Anc.)	-2.707	-1.567	-1.765	-3.325	-3.105	-2.762	-2.020	-2.103
	(0.787)	(0.904)	(0.867)	(0.754)	(0.764)	(0.782)	(0.847)	(0.842)
Post-1500 CE Yield Ch. (Anc.)	-1.107	-1.118	-0.945	-1.137	-1.066	-1.048	-1.027	-1.064
	(0.108)	(0.155)	(0.240)	(0.112)	(0.146)	(0.150)	(0.154)	(0.146)
Observations	647	647	647	647	647	647	647	647
R-squared	0.762	0.721	0.674	0.734	0.747	0.746	0.746	0.744
**PANEL B RESTAURANTS**	**(1)**	**(2)**	**(3)**	**(4)**	**(5)**	**(6)**	**(7)**	**(8)**
Pre-1500 CE Yield (Anc.)	1.314	3.460	2.084	0.185	0.0630	0.262	0.732	0.504
	(0.893)	(0.752)	(0.851)	(0.983)	(0.992)	(0.967)	(0.929)	(0.952)
Post-1500 CE Yield Ch. (Anc.)	-1.117*	-1.064	-1.035	-1.092*	-1.036	-1.041	-1.032	-1.062*
	(0.074)	(0.124)	(0.133)	(0.097)	(0.115)	(0.114)	(0.105)	(0.096)
Observations	675	675	675	675	675	675	675	675
R-squared	0.787	0.755	0.743	0.762	0.774	0.778	0.777	0.773
**PANEL C CLOTHING STORES**	**(1)**	**(2)**	**(3)**	**(4)**	**(5)**	**(6)**	**(7)**	**(8)**
Pre-1500 CE Yield (Anc.)	-5.061	-5.238	-4.572	-6.535	-5.295	-4.762	-4.645	-5.035
	(0.551)	(0.574)	(0.604)	(0.464)	(0.534)	(0.565)	(0.573)	(0.555)
Post-1500 CE Yield Ch. (Anc.)	-1.372***	-1.453***	-1.279**	-1.407***	-1.239**	-1.218**	-1.261**	-1.324**
	(0.011)	(0.012)	(0.037)	(0.013)	(0.025)	(0.024)	(0.016)	(0.015)
Observations	662	662	662	662	662	662	662	662
R-squared	0.799	0.768	0.704	0.768	0.800	0.802	0.801	0.787
**PANEL D MOVIE THEATERS (EXCEPT DRIVE-INS)**	**(1)**	**(2)**	**(3)**	**(4)**	**(5)**	**(6)**	**(7)**	**(8)**
Pre-1500 CE Yield (Anc.)	6.993	7.531	9.445	7.884	5.235	3.888	4.416	4.561
	(0.598)	(0.595)	(0.520)	(0.585)	(0.686)	(0.757)	(0.738)	(0.719)
Post-1500 CE Yield Ch. (Anc.)	-0.928	-1.031*	-0.797	-0.962	-1.049	-1.029	-1.091	-0.962
	(0.189)	(0.058)	(0.189)	(0.137)	(0.118)	(0.127)	(0.108)	(0.132)
Observations	490	490	490	490	490	490	490	490
R-squared	0.733	0.742	0.718	0.697	0.696	0.713	0.740	0.727
**PANEL E FITNESS/RECREATION CENTERS**	**(1)**	**(2)**	**(3)**	**(4)**	**(5)**	**(6)**	**(7)**	**(8)**
Pre-1500 CE Yield (Anc.)	-4.867	-4.216	-5.655	-7.177	-6.758	-6.634	-5.760	-6.353
	(0.555)	(0.613)	(0.453)	(0.346)	(0.402)	(0.378)	(0.448)	(0.4094)
Post-1500 CE Yield Ch. (Anc.)	-1.239**	-1.254**	-1.247*	-1.319**	-1.233**	-1.201**	-1.138**	-1.185**
	(0.0270)	(0.0290)	(0.0280)	(0.0200)	(0.037)	(0.039)	(0.041)	(0.032)
Observations	668	668	668	668	668	668	668	668
R-squared	0.825	0.812	0.788	0.800	0.802	0.810	0.819	0.817
Geographic	Y	Y	Y	Y	Y	Y	Y	Y
Socio-Economic, Pol., Health	Y	Y	Y	Y	Y	Y	Y	Y
State Dummies	Y	Y	Y	Y	Y	Y	Y	Y

Notes: "()" reports the *p*-values of the wild cluster bootstrap method using the post-estimation command boottest. Stars $^{***(**)[*]}$ indicate significance at the 0.01(0.05)[0.1] level. This table reports the effect of the ancestry-adjusted pre-1500 CE crop yield and ancestry-adjusted post-1500 crop yield change on the total number of visitors by commuting zone to each of five POIs (which is calculated by summing up total visitors from all counties in each commuting zones for each POI): 1) hobby centers, 2) restaurants, 3) clothing stores, 4) movie theaters, and) fitness centers. All columns include state dummies; geographic factors that include average roughness of terrain, precipitation, temperature, and elevation; average number of large central or large fringe metro counties and medium metro and small metro counties; mean income; proportion of males; average COVID-19 tests per 100,000 conducted in the commuting zone in the month when the regression is estimated; population density; proportion of population from a Black or African American, Native American, White, and Hispanic or Latino background; proportion of population using public transport; proportion of family and non-family households living in two or more unit structures; proportion of family and non-family households with three or more members; proportion of population in age groups younger than 19, 19–34, and 35–64 (with 65 and older as the omitted group); proportion working from home; proportion of population with an education level higher than or equal to the higher secondary level; healthcare coverage measured as the proportion of the population with two or more health insurance policies in the age groups younger than 19, 19–34, 35–64, and 65 and older, and the proportion of the population older than age 65 without any health insurance; distance to an airport with direct international flights to high-severity countries; Gini index and proportion of population below the poverty line; Social Capital Index; percentage of adult population with obesity; percentage of population who smoke as adults; 30-day risk-adjusted mortality rate; heart disease death rate; percentage of population diagnosed with diabetes among adults older than 20 years of age; contribution to the percentage change in GDP by private-services-providing industries, private-goods-providing industries, and government enterprises, and government enterprises; percentage change in annual average employment for a given year; and proportion of votes for Democrats in the 2016 US presidential election. All variables are normalized by subtracting their mean and dividing by their standard deviation. Therefore, all coefficients are comparable and estimate the effect of a one standard deviation increase in the independent variable.

Table B.29. EFFECT OF LONG-TERM ORIENTATION TRAITS ON MOBILITY PROXIES AT THE COMMUTING ZONE LEVEL: VISITS

TOTAL VISITS BY COUNTY RESIDENTS, MARCH-NOVEMBER 2020								
**PANEL A HOBBY CENTERS**	**(1)**	**(2)**	**(3)**	**(4)**	**(5)**	**(6)**	**(7)**	**(8)**
	March	April	May	June	July	August	September	October
Pre-1500 CE Yield (Anc.)	-3.390	-1.562	-2.336	-3.873	-3.669	-3.671	-2.801	-2.250
	(0.721)	(0.869)	(0.812)	(0.700)	(0.720)	(0.709)	(0.770)	(0.811)
Post-1500 CE Yield Ch. (Anc.)	-1.108	-1.099	-0.955	-1.153	-1.085	-1.082	-1.042	-1.051
	(0.119)	(0.149)	(0.226)	(0.117)	(0.146)	(0.147)	(0.151)	(0.154)
Observations	556	556	556	556	556	556	556	556
R-squared	0.771	0.735	0.690	0.743	0.754	0.754	0.755	0.751
**PANEL B RESTAURANTS**	**(1)**	**(2)**	**(3)**	**(4)**	**(5)**	**(6)**	**(7)**	**(8)**
Pre-1500 CE Yield (Anc.)	1.018	3.011	1.780	-0.0759	-0.111	0.0359	0.335	0.177
	(0.905)	(0.773)	(0.854)	(0.992)	(0.987)	(0.997)	(0.966)	(0.979)
Post-1500 CE Yield Ch. (Anc.)	-1.100*	-1.059	-1.026	-1.074	-1.015	-1.019	-1.011	-1.044*
	(0.070)	(0.120)	(0.131)	(0.101)	(0.112)	(0.109)	(0.104)	(0.097)
Observations	675	675	675	675	675	675	675	675
R-squared	0.793	0.761	0.750	0.768	0.780	0.784	0.782	0.777
**PANEL C CLOTHING STORES**	**(1)**	**(2)**	**(3)**	**(4)**	**(5)**	**(6)**	**(7)**	**(8)**
Pre-1500 CE Yield (Anc.)	-5.552	-6.251	-5.520	-6.983	-5.577	-5.331	-5.113	-5.405
	(0.514)	(0.481)	(0.496)	(0.397)	(0.501)	(0.506)	(0.522)	(0.513)
Post-1500 CE Yield Ch. (Anc.)	-1.408***	-1.533***	-1.390***	-1.446***	-1.288***	-1.265**	-1.284**	-1.336***
	(0.006)	(0.009)	(0.010)	(0.008)	(0.010)	(0.012)	(0.011)	(0.01)
Observations	640	640	640	640	640	640	640	640
R-squared	0.786	0.755	0.723	0.768	0.795	0.797	0.794	0.781
**PANEL D MOVIE THEATERS (EXCEPT DRIVE-INS)**	**(1)**	**(2)**	**(3)**	**(4)**	**(5)**	**(6)**	**(7)**	**(8)**
Pre-1500 CE Yield (Anc.)	7.097	5.479	6.233	6.431	4.019	2.132	3.262	3.544
	(0.589)	(0.693)	(0.681)	(0.684)	(0.796)	(0.871)	(0.795)	(0.779)
Post-1500 CE Yield Ch. (Anc.)	-0.935	-1.070*	-0.915	-1.039	-1.060	-1.091*	-1.089*	-0.986
	(0.144)	(0.074)	(0.158)	(0.116)	(0.106)	(0.067)	(0.067)	(0.103)
Observations	431	431	431	431	431	431	431	431
R-squared	0.747	0.754	0.740	0.716	0.718	0.728	0.739	0.730
**PANEL E FITNESS/RECREATION CENTERS**	**(1)**	**(2)**	**(3)**	**(4)**	**(5)**	**(6)**	**(7)**	**(8)**
Pre-1500 CE Yield (Anc.)	-4.320	-3.946	-4.796	-7.065	-7.498	-7.153	-6.406	-6.412
	(0.618)	(0.656)	(0.545)	(0.357)	(0.379)	(0.365)	(0.423)	(0.432)
Post-1500 CE Yield Ch. (Anc.)	-1.214**	-1.230**	-1.189**	-1.284**	-1.251**	-1.205**	-1.142**	-1.167**
	(0.015)	(0.015)	(0.015)	(0.011)	(0.023)	(0.028)	(0.0390)	(0.029)
Observations	640	640	640	640	640	640	640	640
R-squared	0.824	0.814	0.785	0.789	0.791	0.795	0.810	0.810
Geographic	Y	Y	Y	Y	Y	Y	Y	Y
Socio-Economic, Pol., Health	Y	Y	Y	Y	Y	Y	Y	Y
State Dummies	Y	Y	Y	Y	Y	Y	Y	Y

Notes: "()" reports the *p*-values of the wild cluster bootstrap method using the post-estimation command boottest. Stars $^{***(**)[*]}$ indicate significance at the 0.01(0.05)[0.1] level. This table reports the effect of the ancestry-adjusted pre-1500 CE crop yield and ancestry-adjusted post-1500 crop yield change on the total number of visitors by commuting zone to each of five POIs (which is calculated by summing up total visits from all counties in each commuting zones for each POI): 1) hobby centers, 2) restaurants, 3) clothing stores, 4) movie theaters, and 5) fitness centers. All columns include state dummies; geographic factors that include average roughness of terrain, precipitation, temperature, and elevation; average number of large central or large fringe metro counties and medium metro and small metro counties; mean income; proportion of males; average COVID-19 tests per 100,000 conducted in the commuting zone in the month when the regression is estimated; population density; proportion of population from a Black or African American, Native American, White, and Hispanic or Latino background; proportion of population using public transport; proportion of family and non-family households living in two or more unit structures; proportion of family and non-family households with three or more members; proportion of population in age groups younger than 19, 19–34, and 35–64 (with 65 and older as the omitted group); proportion working from home; proportion of population with an education level higher than or equal to the higher secondary level; healthcare coverage measured as the proportion of the population with two or more health insurance policies in the age groups younger than 19, 19–34, 35–64, and 65 and older, and the proportion of the population older than age 65 without any health insurance; distance to an airport with direct international flights to high-severity countries; Gini index and proportion of population below the poverty line; Social Capital Index; percentage of adult population with obesity; percentage of population who smoke as adults; 30-day risk-adjusted mortality rate; heart disease death rate; percentage of population diagnosed with diabetes among adults older than 20 years of age; contribution to the percentage change in GDP by private-services-providing industries, private-goods-providing industries, and government enterprises, and government enterprises; percentage change in annual average employment for a given year; and proportion of votes for Democrats in the 2016 US presidential election. All variables are normalized by subtracting their mean and dividing by their standard deviation. Therefore, all coefficients are comparable and estimate the effect of a one standard deviation increase in the independent variable.

Table B.30. EFFECT ON MOBILITY PROXY AT THE COMMUTING ZONE LEVEL: GOOGLE DATA

GOOGLE MOBILITY REPORT MARCH-OCTOBER 2020								
	March	April	May	June	July	August	September	October
**PANEL A: PERC. CH. VISITS TO RECREATION CENTERS**	**(1)**	**(2)**	**(3)**	**(4)**	**(5)**	**(6)**	**(7)**	**(8)**
Pre-1500 CE Yield (Anc.)	-2.681	0.653	1.509	0.135	0.814	0.978	1.184	1.938
	(0.5526)	(0.8789)	(0.7948)	(0.9790)	(0.8529)	(0.8418)	(0.8068)	(0.6837)
Post-1500 CE Yield Ch. (Anc.)	-0.473**	-0.418*	-0.325	-0.179	-0.0951	-0.147	-0.0903	-0.295
	(0.0430)	(0.0851)	(0.1702)	(0.4324)	(0.7047)	(0.5495)	(0.6947)	(0.230)
Observations	552	552	552	552	552	552	552	552
R-squared	0.758	0.742	0.682	0.624	0.624	0.623	0.617	0.616
**PANEL B: PERC. CH. IN HOURS AWAY FROM HOME**	**(1)**	**(2)**	**(3)**	**(4)**	**(5)**	**(6)**	**(7)**	**(8)**
Pre-1500 CE Yield (Anc.)	-0.662	-0.645	-2.901	-7.674*	-6.239	-2.297	-5.925*	-3.842
	(0.8779)	(0.8899)	(0.5005)	(0.0621)	(0.1722)	(0.6847)	(0.1381)	(0.4515)
Post-1500 CE Yield Ch. (Anc.)	-0.0918	-0.394*	-0.361*	-0.520**	-0.537***	-0.269	-0.284*	-0.105
	(0.6767)	(0.0511)	(0.0881)	(0.0120)	(0.0080)	(0.2302)	(0.0601)	(0.6446)
Observations	468	468	468	468	468	468	468	468
R-squared	0.732	0.825	0.816	0.834	0.834	0.801	0.865	0.858
Geographic	Y	Y	Y	Y	Y	Y	Y	Y
Socio-Economic, Pol., Health	Y	Y	Y	Y	Y	Y	Y	Y
State Dummies	Y	Y	Y	Y	Y	Y	Y	Y

Notes: "()" reports the p-values of the wild cluster bootstrap method using the post-estimation command boottest. Stars $^{***(**)[*]}$ indicate significance at the $0.01(0.05)[0.1]$ level. This table reports the effect of the ancestry-adjusted pre-1500 CE crop yield and ancestry-adjusted post-1500 crop yield change on the average percentage change in visits to recreational centers and the average percentage change in hours spent away from home using mobility data from Google mobility reports at the commuting zone level. The outcome variables are measured relative to the pre-crisis level (i.e., between January 3 and February 6, 2020). The monthly data are from the 15th of each month to the 14th of the following month. All columns include state dummies; geographic factors that include average roughness of terrain, precipitation, temperature, and elevation; average number of large central or large fringe metro counties and medium metro and small metro counties; mean income; proportion of males; average COVID-19 tests per 100,000 conducted in the commuting zone in the month when the regression is estimated; population density; proportion of population from a Black or African American, Native American, White, and Hispanic or Latino background; proportion of population using public transport; proportion of family and non-family households living in two or more unit structures; proportion of family and non-family households with three or more members; proportion of population in age groups younger than 19, 19–34, and 35–64 (with 65 and older as the omitted group); proportion working from home; proportion of population with an education level higher than or equal to the higher secondary level; healthcare coverage measured as the proportion of the population with two or more health insurance policies in the age groups younger than 19, 19–34, 35–64, and 65 and older, and the proportion of the population older than age 65 without any health insurance; distance to an airport with direct international flights to high-severity countries; Gini index and proportion of population below the poverty line; Social Capital Index; percentage of adult population with obesity; percentage of population who smoke as adults; 30-day risk-adjusted mortality rate; heart disease death rate; percentage of population diagnosed with diabetes among adults older than 20 years of age; contribution to the percentage change in GDP by private-services-providing industries, private-goods-providing industries, and government enterprises, and government enterprises; percentage change in annual average employment for a given year; and proportion of votes for Democrats in the 2016 US presidential election. All variables are normalized by subtracting their mean and dividing by their standard deviation. Therefore, all coefficients are comparable and estimate the effect of a one standard deviation increase in the independent variable.

Table B.31. DYNAMIC EFFECT OF LONG-TERM ORIENTATION TRAITS ON COVID-19 SEVERITY AT THE COMMUTING ZONE LEVEL

	COVID-19 case-prevalence	COVID-19 death-prevalence	COVID-19 case-prevalence	COVID-19 death-prevalence
High pre-1500 CE Yield (Anc.) *May	-0.485***	-0.339***		
	(0.0000)	(0.007)		
High Pre-1500 CE Yield (Anc.)*Jun	-0.441***	-0.354***		
	(0.0000)	(0.0010)		
High Pre-1500 CE Yield (Anc.)*Jul	-0.438***	-0.380***		
	(0.0020)	(0.0020)		
High Pre-1500 CE Yield (Anc.)*Aug	-0.465***	-0.415***		
	(0.0020)	(0.0040)		
High Pre-1500 CE Yield (Anc.)*Sep	-0.438***	-0.397***		
	(0.0050)	(0.0060)		
High Pre-1500 CE Yield (Anc.)*Oct	-0.412***	-0.395***		
	(0.0040)	(0.0030)		
High Pre-1500 CE Yield (Anc.)*Nov	-0.391***	-0.403***		
	(0.0090)	(0.0050)		
High Post-1500 CE Yield Ch. (Anc.)*May			-0.350*	-0.319**
			(0.0981)	(0.0280)
High Post-1500 CE Yield Ch. (Anc.)*June			-0.316	-0.317**
			(0.1161)	(0.0300)
High Post-1500 CE Yield Ch. (Anc.)*July			-0.289	-0.323**
			(0.1512)	(0.0300)
High Post-1500 CE Yield Ch. (Anc.)*Aug			-0.313	-0.364*
			(0.1962)	(0.0521)
High Post-1500 CE Yield Ch. (Anc.)*Sep			-0.266	-0.324*
			(0.2613)	(0.0731)
High Post-1500 CE Yield Ch. (Anc.)*Oct			-0.218	-0.310*
			(0.3914)	(0.0781)
High Post-1500 CE Yield Ch. (Anc.)*Nov			-0.178	-0.291*
			(0.5125)	(0.0861)
Observations	5,540	5,540	5,540	5,540
R-squared	0.178	0.199	0.176	0.198
Number of Counties	700	700	700	700
State*Trends	Yes	Yes	Yes	Yes
Geographic Controls	Yes	Yes	Yes	Yes

"()" reports the *p*-values of the wild cluster bootstrap method using the post-estimation command boottest. Stars $^{***(**)[*]}$ indicate significance at the 0.01(0.05)[0.1] level. This table presents the effect of the ancestry-adjusted high pre-1500 CE potential crop yield (where ancestry-adjusted high pre-1500 CE potential crop yield is a dummy variable that equals 1 if the ancestry-adjusted potential crop yield is above the sample median) and the ancestry-adjusted high post-1500 CE potential crop yield change during the Columbian Exchange (where ancestry-adjusted high post-1500 potential yield change is a dummy variable that equals 1 if the potential crop yield change is above the sample median) on average COVID-19 case prevalence and COVID-19 death prevalence at the commuting zone level for every month from April 1, 2020 to November 30, 2020. The reported effects are relative to the month of April 2020. COVID-19 monthly case prevalence is measured as total COVID-19 cases per tests per 100,000 averaged over counties within a commuting zone. COVID-19 monthly death prevalence is measured as total COVID-19 deaths per tests per 100,000 averaged over counties within a commuting zone. All columns control for temperature and precipitation at the commuting zone level and state-specific time trends. All variables are normalized by subtracting their mean and dividing by their standard deviation. Therefore, all coefficients are comparable and estimate the effect of a one standard deviation increase in the independent variable.

Table B.32. DYNAMIC EFFECT OF LONG-TERM ORIENTATION TRAITS ON MOBILITY AT THE COMMUTING ZONE LEVEL: HOBBY CENTERS AND GAMING STORES

**HOBBY CENTERS AND GAMING STORES**	**(1)**	**(2)**	**(3)**	**(4)**
Total	Visitors	Visits	Visitors	Visits
High Pre-1500 CE Yield (Anc.)*Mar	-0.0740**	-0.0829**		
	(0.044)	(0.039)		
High Pre-1500 CE Yield (Anc.)*Apr	-0.162*	-0.170**		
	(0.054)	(0.041)		
High Pre-1500 CE Yield (Anc.)*May	-0.0692	-0.0899*		
	(0.185)	(0.084)		
High Pre-1500 CE Yield (Anc.)*Jun	0.0502	0.0153		
	(0.251)	(0.699)		
High Pre-1500 CE Yield (Anc.)*Jul	0.105**	0.0655		
	(0.037)	(0.146)		
High Pre-1500 CE Yield (Anc.)*Aug	0.156***	0.113**		
	(0.0050)	(0.049)		
High Pre-1500 CE Yield (Anc.)*Sep	0.163**	0.117*		
	(0.0120)	(0.054)		
High Pre-1500 CE Yield (Anc.)*Oct	0.194***	0.143**		
	(0.003)	(0.022)		
High Post-1500 CE Yield Ch. (Anc.)*Mar			-0.106***	-0.113***
			(0.002)	(0.002)
High Post-1500 CE Yield Ch. (Anc.)*Apr			-0.227***	-0.232***
			(0.009)	(0.008)
High Post-1500 CE Yield Ch. (Anc.)*May			-0.196***	-0.203***
			(0.000)	(0.000)
High Post-1500 CE Yield Ch. (Anc.)*Jun			-0.0648**	-0.0814**
			(0.026)	(0.012)
High Post-1500 CE Yield Ch. (Anc.)*Jul			-0.0369	-0.0525*
			(0.182)	(0.081)
High Post-1500 CE Yield Ch. (Anc.)*Aug			-0.00840	-0.0224
			(0.765)	(0.447)
High Post-1500 CE Yield Ch. (Anc.)*Sep			-0.0287	-0.0413
			(0.417)	(0.232)
High Post-1500 CE Yield Ch. (Anc.)*Oct			-0.0278	-0.0420
			(0.423)	(0.187)
Observations	4,051	4,051	4,051	4,051
R-squared	0.256	0.237	0.255	0.238
Number of commuting zone	472	472	472	472
State*Trends	Yes	Yes	Yes	Yes
Geographic Controls	Yes	Yes	Yes	Yes

Notes: "()" reports the *p*-values of the wild cluster bootstrap method using the post-estimation command boottest. Stars $^{***(**)[*]}$ indicate significance at the 0.01(0.05)[0.1] level. This table presents the effect of the ancestry-adjusted high pre-1500 CE potential crop yield (where ancestry-adjusted high pre-1500 CE potential crop yield is a dummy variable that equals 1 if the ancestry-adjusted potential crop yield is above the sample median) and the ancestry-adjusted high post-1500 CE potential crop yield change during the Columbian Exchange (where ancestry-adjusted high post-1500 potential yield change is a dummy variable that equals 1 if the potential crop yield change is above the sample median) on monthly total number of visitors and visits to hobby centers and gaming stores at the commuting zone level from February 1, 2020 to October 31, 2020. The reported effects are relative to the month of February 2020. All columns control for temperature, precipitation, and state-specific time trends. All variables are normalized by subtracting their mean and dividing by their standard deviation. Therefore, all coefficients are comparable and estimate the effect of a one standard deviation increase in the independent variable.

Table B.33. DYNAMIC EFFECT OF LONG-TERM ORIENTATION ON MOBILITY AT COMMUTING ZONE LEVEL: RESTAURANTS

**RESTAURANTS**	**(1)**	**(2)**	**(3)**	**(4)**
Total	Visitors	Visits	Visitors	Visits
High Pre-1500 CE Yield (Anc.)*Mar	-0.129***	-0.148***		
	(0.003)	(0.001)		
High Pre-1500 CE Yield (Anc.)*Apr	-0.252***	-0.270***		
	(0.008)	(0.006)		
High Pre-1500 CE Yield (Anc.)*May	-0.209***	-0.231***		
	(0.008)	(0.005)		
High Pre-1500 CE Yield (Anc.)*Jun	-0.162**	-0.190***		
	(0.020)	(0.008)		
High Pre-1500 CE Yield (Anc.)*Jul	-0.150*	-0.177**		
	(0.071)	(0.039)		
High Pre-1500 CE Yield (Anc.)*Aug	-0.143	-0.172*		
	(0.094)	(0.051)		
High Pre-1500 CE Yield (Anc.)*Sep	-0.188**	-0.218**		
	(0.048)	(0.031)		
High Pre-1500 CE Yield (Anc.)*Oct	-0.201**	-0.233**		
	(0.038)	(0.019)		
High Post-1500 CE Yield Ch. (Anc.)*Mar			-0.121***	-0.141***
			(0.004)	(0.004)
High Post-1500 CE Yield Ch. (Anc.)*Apr			-0.235**	-0.257***
			(0.016)	(0.009)
High Post-1500 CE Yield Ch. (Anc.)*May			-0.208***	-0.230***
			(0.009)	(0.006)
High Post-1500 CE Yield Ch. (Anc.)*Jun			-0.164**	-0.190***
			(0.023)	(0.008)
High Post-1500 CE Yield Ch. (Anc.)*Jul			-0.154*	-0.179**
			(0.0541)	(0.017)
High Post-1500 CE Yield Ch. (Anc.)*Aug			-0.143*	-0.167*
			(0.0921)	(0.058)
High Post-1500 CE Yield Ch. (Anc.)*Sep			-0.188**	-0.210**
			(0.039)	(0.028)
High Post-1500 CE Yield Ch. (Anc.)*Oct			-0.211**	-0.232***
			(0.012)	(0.007)
Observations	4,051	4,051	4,051	4,051
R-squared	0.191	0.195	0.190	0.194
Number of commuting zone	472	472	472	472
State*Trends	Yes	Yes	Yes	Yes
Geographic Controls	Yes	Yes	Yes	Yes

"()" reports the p-values of the wild cluster bootstrap method using the post-estimation command boottest. Stars $^{***(**)[*]}$ indicate significance at the $0.01(0.05)[0.1]$ level. This table presents the effect of the ancestry-adjusted high pre-1500 CE potential crop yield (where ancestry-adjusted high pre-1500 CE potential crop yield is a dummy variable that equals 1 if the ancestry-adjusted potential crop yield is above the sample median) and the ancestry-adjusted high post-1500 CE potential crop yield change during the Columbian Exchange (where ancestry-adjusted high post-1500 potential yield change is a dummy variable that equals 1 if the potential crop yield change is above the sample median) on monthly total visitors and visits to restaurants at the commuting zone level from February 1, 2020 to October 31, 2020. The reported effects are relative to the month of February 2020. All columns control for temperature, precipitation, and state-specific time trends. All variables are normalized by subtracting their mean and dividing by their standard deviation. Therefore, all coefficients are comparable and estimate the effect of a one standard deviation increase in the independent variable.

Table B.34. DYNAMIC EFFECT OF LONG-TERM ORIENTATION TRAITS ON MOBILITY AT THE COMMUTING ZONE LEVEL: MOVIE THEATERS (EXCEPT DRIVE-INS)

**MOVIE THEATERS (EXCEPT DRIVE-INS)**	**(1)**	**(2)**	**(3)**	**(4)**
Total	Visitors	Visits	Visitors	Visits
High Pre-1500 CE Yield (Anc.)*Mar	-0.562***	-0.516***		
	(0.000)	(0.000)		
High Pre-1500 CE Yield (Anc.)*Apr	-0.890***	-0.811***		
	(0.002)	(0.007)		
High Pre-1500 CE Yield (Anc.)*May	-1.056***	-0.969***		
	(0.000)	(0.001)		
High Pre-1500 CE Yield (Anc.)*Jun	-1.219***	-1.114***		
	(0.000)	(0.000)		
High Pre-1500 CE Yield (Anc.)*Jul	-1.400***	-1.278***		
	(0.000)	(0.000)		
High Pre-1500 CE Yield (Anc.)*Aug	-1.531***	-1.396***		
	(0.000)	(0.003)		
High Pre-1500 CE Yield (Anc.)*Sep	-1.736***	-1.588***		
	(0.001)	(0.001)		
High Pre-1500 CE Yield (Anc.)*Oct	-1.929***	-1.773***		
	(0.000)	(0.001)		
High Post-1500 CE Yield Ch. (Anc.)*Mar			-0.403***	-0.373***
			(0.003)	(0.008)
High Post-1500 CE Yield Ch. (Anc.)*Apr			-0.545**	-0.520*
			(0.046)	(0.064)
High Post-1500 CE Yield Ch. (Anc.)*May			-0.582**	-0.569**
			(0.0340)	(0.049)
High Post-1500 CE Yield Ch. (Anc.)*Jun			-0.634**	-0.633*
			(0.039)	(0.052)
High Post-1500 CE Yield Ch. (Anc.)*Jul			-0.702**	-0.702**
			(0.023)	(0.034)
High Post-1500 CE Yield Ch. (Anc.)*Aug			-0.695*	-0.702*
			(0.070)	(0.087)
High Post-1500 CE Yield Ch. (Anc.)*Sep			-0.763*	-0.775*
			(0.056)	(0.072)
High Post-1500 CE Yield Ch. (Anc.)*Oct			-0.799*	-0.824*
			(0.072)	(0.093)
Observations	4,051	4,051	4,051	4,051
R-squared	0.274	0.252	0.252	0.233
Number of commuting zone	472	472	472	472
State*Trends	Yes	Yes	Yes	Yes
Geographic Controls	Yes	Yes	Yes	Yes

Notes: "()" reports the p-values of the wild cluster bootstrap method using the post-estimation command boottest. Stars $^{***(**)[*]}$ indicate significance at the 0.01(0.05)[0.1] level. This table presents the effect of the ancestry-adjusted high pre-1500 CE potential crop yield (where ancestry-adjusted high pre-1500 CE potential crop yield is a dummy variable that equals 1 if the ancestry-adjusted potential crop yield is above the sample median) and the ancestry-adjusted high post-1500 CE potential crop yield change during the Columbian Exchange (where ancestry-adjusted high post-1500 potential yield change is a dummy variable that equals 1 if the potential crop yield change is above the sample median) on monthly total visitors and visits to movie theaters (except drive-ins) at the commuting zone level from February 1, 2020 to October 31, 2020. The reported effects are relative to the month of February 2020. All columns control for geographic controls such as temperature, precipitation, and state-specific time trends. All variables are normalized by subtracting their mean and dividing by their standard deviation. Therefore, all coefficients are comparable and estimate the effect of a one standard deviation increase in the independent variable.

Table B.35. DYNAMIC EFFECT OF LONG-TERM ORIENTATION TRAITS ON MOBILITY AT THE COMMUTING ZONE LEVEL: CLOTHING STORES

**CLOTHING CENTERS**	**(1)**	**(2)**	**(3)**	**(4)**
Total	Visitors	Visits	Visitors	Visits
High Pre-1500 CE Yield (Anc.)*Mar	-0.155***	-0.162***		
	(0.005)	(0.006)		
High Pre-1500 CE Yield (Anc.)*Apr	-0.300***	-0.299***		
	(0.010)	(0.012)		
High Pre-1500 CE Yield (Anc.)*May	-0.236**	-0.250**		
	(0.0110)	(0.012)		
High Pre-1500 CE Yield (Anc.)*Jun	-0.0918	-0.132**		
	(0.196)	(0.103)		
High Pre-1500 CE Yield (Anc.)*Jul	-0.0371	-0.0902		
	(0.609)	(0.308)		
High Pre-1500 CE Yield (Anc.)*Aug	-0.0189	-0.0788		
	(0.780)	(0.402)		
High Pre-1500 CE Yield (Anc.)*Sep	-0.0469	-0.112		
	(0.576)	(0.223)		
High Pre-1500 CE Yield (Anc.)*Oct	-0.0420	-0.114		
	(0.614)	(0.255)		
High Post-1500 CE Yield Ch. (Anc.)*Mar			-0.149***	-0.154***
			(0.007)	(0.009)
High Post-1500 CE Yield Ch. (Anc.)*Apr			-0.285**	-0.288**
			(0.016)	(0.015)
High Post-1500 CE Yield Ch. (Anc.)*May			-0.261***	-0.269***
			(0.007)	(0.007)
High Post-1500 CE Yield Ch. (Anc.)*Jun			-0.141**	-0.167**
			(0.032)	(0.032)
High Post-1500 CE Yield Ch. (Anc.)*Jul			-0.105*	-0.141*
			(0.084)	(0.061)
High Post-1500 CE Yield Ch. (Anc.)*Aug			-0.0931*	-0.128*
			(0.080)	(0.0721)
High Post-1500 CE Yield Ch. (Anc.)*Sep			-0.129**	-0.167**
			(0.030)	(0.024)
High Post-1500 CE Yield Ch. (Anc.)*Oct			-0.146***	-0.184***
			(0.006)	(0.007)
Observations	4,051	4,051	4,051	4,051
R-squared	0.172	0.168	0.168	0.166
Number of commuting zones	472	472	472	472
State*Trends	Yes	Yes	Yes	Yes
Geographic Controls	Yes	Yes	Yes	Yes

Notes: "()" reports the p-values of the wild cluster bootstrap method using the post-estimation command boottest. Stars $^{***(**)[*]}$ indicate significance at the 0.01(0.05)[0.1] level. This table presents the effect of the ancestry-adjusted high pre-1500 CE potential crop yield (where ancestry-adjusted high pre-1500 CE potential crop yield is a dummy variable that equals 1 if the ancestry-adjusted potential crop yield is above the sample median) and the ancestry-adjusted high post-1500 CE potential crop yield change during the Columbian Exchange (where ancestry-adjusted high post-1500 potential yield change is a dummy variable that equals 1 if the potential crop yield change is above the sample median) on the monthly total number of visitors and visits to clothing stores at the commuting zone level from February 1, 2020 to October 31, 2020. The reported effects are relative to the month of February 2020. All columns control for geographic controls such as temperature, precipitation, and state-specific time trends. All variables are normalized by subtracting their mean and dividing by their standard deviation. Therefore, all coefficients are comparable and estimate the effect of a one standard deviation increase in the independent variable.

Table B.36. DYNAMIC EFFECT OF LONG-TERM ORIENTATION TRAITS ON MOBILITY AT THE COMMUTING ZONE LEVEL: FITNESS AND RECREATION CENTERS

**FITNESS AND RECREATION CENTERS**	**(1)**	**(2)**	**(3)**	**(4)**
Total	Visitors	Visits	Visitors	Visits
High Pre-1500 CE Yield (Anc.)*Mar	-0.214***	-0.265***		
	(0.000)	(0.000)		
High Pre-1500 CE Yield (Anc.)*Apr	-0.470***	-0.507***		
	(0.000)	(0.000)		
High Pre-1500 CE Yield (Anc.)*May	-0.461***	-0.513***		
	(0.000)	(0.000)		
High Pre-1500 CE Yield (Anc.)*Jun	-0.422***	-0.480***		
	(0.000)	(0.000)		
High Pre-1500 CE Yield (Anc.)*Jul	-0.429***	-0.477***		
	(0.000)	(0.001)		
High Pre-1500 CE Yield (Anc.)*Aug	-0.437***	-0.489***		
	(0.002)	(0.002)		
High Pre-1500 CE Yield (Anc.)*Sep	-0.491***	-0.532***		
	(0.002)	(0.003)		
High Pre-1500 CE Yield (Anc.)*Oct	-0.525***	-0.563***		
	(0.000)	(0.001)		
High Post-1500 CE Yield Ch. (Anc.)*Mar			-0.187***	-0.237***
			(0.000)	(0.000)
High Post-1500 CE Yield Ch. (Anc.)*Apr			-0.409***	-0.444***
			(0.001)	(0.003)
High Post-1500 CE Yield Ch. (Anc.)*May			-0.396***	-0.451***
			(0.002)	(0.002)
High Post-1500 CE Yield Ch. (Anc.)*Jun			-0.348***	-0.417***
			(0.000)	(0.000)
High Post-1500 CE Yield Ch. (Anc.)*Jul			-0.335***	-0.386***
			(0.003)	(0.002)
High Post-1500 CE Yield Ch. (Anc.)*Aug			-0.318**	-0.375**
			(0.012)	(0.011)
High Post-1500 CE Yield Ch. (Anc.)*Sep			-0.351***	-0.400**
			(0.009)	(0.008)
High Post-1500 CE Yield Ch. (Anc.)*Oct			-0.369***	-0.414***
			(0.003)	(0.003)
Observations	4,051	4,051	4,051	4,051
R-squared	0.191	0.195	0.187	0.192
Number of commuting zones	472	472	472	472
State*Trends	Yes	Yes	Yes	Yes
Geographic Controls	Yes	Yes	Yes	Yes

Notes: "()" reports the p-values of the wild cluster bootstrap method using the post-estimation command boottest. Stars $^{***(**)[*]}$ indicate significance at the 0.01(0.05)[0.1] level. This table presents the effect of the ancestry-adjusted high pre-1500 CE potential crop yield (where ancestry-adjusted high pre-1500 CE potential crop yield is a dummy variable that equals 1 if the ancestry-adjusted potential crop yield is above the sample median) and the ancestry-adjusted high post-1500 CE potential crop yield change during the Columbian Exchange (where ancestry-adjusted high post-1500 potential yield change is a dummy variable that equals 1 if the potential crop yield change is above the sample median) on the monthly total number of visitors and visits to fitness and recreational centers at the commuting zone level from February 1, 2020 to October 31, 2020. The reported effects are relative to the month of February 2020. All columns control for geographic controls such as temperature, precipitation, and state-specific time trends. All variables are normalized by subtracting their mean and dividing by their standard deviation. Therefore, all coefficients are comparable and estimate the effect of a one standard deviation increase in the independent variable.

Table B.37. DYNAMIC EFFECT OF LONG-TERM ORIENTATION TRAITS ON MOBILITY AT THE COMMUTING ZONE LEVEL: GOOGLE MOBILITY REPORT

**GOOGLE MOBILITY REPORTS**	**(1)**	**(2)**	**(3)**	**(4)**
Perc. Ch. in	Hrs Spent Away from Home	Hrs Spent Away from Home	Recreational Visits	Recreational Visits
High Pre-1500 CE Yield (Anc.)*Mar	0.0147		-0.0283	
	(0.828)		(0.5105)	
High Pre-1500 CE Yield (Anc.)*Apr	-0.0750		0.0614	
	(0.200)		(0.3373)	
High Pre-1500 CE Yield (Anc.)*May	-0.103**		-0.0332	
	(0.032)		(0.6466)	
High Pre-1500 CE Yield (Anc.)*June	-0.0917*		-0.194***	
	(0.053)		(0.0080)	
High Pre-1500 CE Yield (Anc.)*July	-0.0689		-0.219**	
	(0.169)		(0.0160)	
High Pre-1500 CE Yield (Anc.)*Aug	-0.0400		-0.110	
	(0.445)		(0.1912)	
High Pre-1500 CE Yield (Anc.)*Sep	-0.0523		-0.0978	
	(0.358)		(0.2923)	
High Pre-1500 CE Yield (Anc.)*Oct	0.0329		-0.0177	
	(0.553)		(0.8769)	
High Post-1500 CE Yield Ch. (Anc.)*Mar		-0.180***		-0.313***
		(0.0010)		(0.0000)
High Post-1500 CE Yield Ch. (Anc.)*Apr		-0.321***		-0.273***
		(0.0000)		(0.0000)
High Post-1500 CE Yield Ch. (Anc.)*May		-0.190***		-0.185***
		(0.0000)		(0.0040)
High Post-1500 CE Yield Ch. (Anc.)*June		-0.0683		-0.148*
		(0.1481)		(0.0571)
High Post-1500 CE Yield Ch. (Anc.)*July		-0.0792		-0.246***
		(0.1021)		(0.0020)
High Post-1500 CE Yield Ch. (Anc.)*Aug		-0.0379		-0.183**
		(0.4815)		(0.0210)
High Post-1500 CE Yield Ch. (Anc.)*Sep		-0.0639		-0.213**
		(0.2523)		(0.0130)
High Post-1500 CE Yield Ch. (Anc.)*Oct		-0.0393		-0.311***
		(0.5225)		(0.0030)
Observations	4,507	4,507	5,364	5,364
R-squared	0.912	0.914	0.763	0.763
No. of commuting zones	546	546	660	660
State*Trends	Yes	Yes	Yes	Yes
Geographic Controls	Yes	Yes	Yes	Yes

"()" reports the *p*-values of the wild cluster bootstrap method using the post-estimation command boottest. Stars $^{***(**)[*]}$ indicate significance at the $0.01(0.05)[0.1]$ level. This table presents the effect of the ancestry-adjusted high pre-1500 CE potential crop yield (where ancestry-adjusted high pre-1500 CE potential crop yield is a dummy variable that equals 1 if the ancestry-adjusted potential crop yield is above the sample median) and the ancestry-adjusted high post-1500 CE potential crop yield change during the Columbian Exchange (where ancestry-adjusted high post-1500 potential yield change is a dummy variable that equals 1 if the potential crop yield change is above the sample median) on the average monthly percentage change in hours spent away from residential places and the average percentage change in visits to recreation centers such as restaurants, cafes, shopping centers, theme parks, museums, libraries, and movie theaters at the commuting zone level. The outcome variables are measured relative to the pre-crisis level (i.e., between January 3, 2020 and February 6, 2020). The monthly data are from the 15th of each month to the 14th of the following month. The reported effects are relative to the outcome variable measured in the month of February 2020. All columns control for geographic variables such as temperature and precipitation and state-specific time trends. All variables are normalized by subtracting their mean and dividing by their standard deviation. Therefore, all coefficients are comparable and estimate the effect of a one standard deviation increase in the independent variable.

Appendix C. Towards an understanding of the mechanism of evolution of time preferences

In this section, we explain the mechanism underlying the persistent effects on contemporary LTO traits, as established in Galor and Özak (2016). The dynamic model in Galor and Özak (2016) captures the evolution of time preferences during the agricultural stage of development, which is a Malthusian era in which individuals who generate more resources are likely to have higher reproductive success (Ashraf and Galor, 2011; Dalgaard and Strulik, 2015; Vollrath, 2011). The theory of the evolution of LTO traits is based on four components: choice between endowment and investment mode of production, learning, reproductive success, and intergenerational transmission. For the first component, individuals with higher LTO traits choose the investment mode of agricultural production over endowment modes of production such as subsistence agriculture, herding, or hunting. The investment mode of agricultural production permits higher but delayed returns.

With the second component, obtaining larger rewards from delayed gratification reinforces the ability of the individuals with LTO traits to be patient and reduces their tendency to discount future gains (Dixon et al., 1998; Logue and Mazur, 1981; Rung and Young, 2015). Regarding the third component, patient individuals experience higher reproductive success from engaging in agricultural ventures that yield higher economic returns. With the fourth component, the offspring of patient individuals inherit their parents' LTO traits and pass these on to the next generation (Bandura and Mischel, 1965). The proportion of patient individuals increases in the population in each generation because of the higher reproductive success arising from access to better resources from profitable investments, while the proportion of population engaged in endowment modes of production declines asymptotically to zero because of the lower level of reproductive success arising from the lower returns from the subsistence mode of production. Therefore, the long-run steady-state level of the average time preference in the economy is determined by the steady-state level of time preferences among individuals engaged in agricultural investment.[4]

An increase in the return from agricultural investment lowers the threshold level of the discount factor above which individuals will choose to invest. Higher returns from agricultural modes of production reinforce LTO traits and increase reproductive success and hence the relative proportion of their traits in the economy given that offspring inherit parental traits. This results in an increase in the long-run steady-state level of time preferences in the economy. Thus, empirically investigating the deep roots of present-day time preferences focuses on variations in potential returns to agricultural investment. Galor and Özak (2016) empirically establish a causal link between return from agricultural investment and contemporary variations in time preferences at the individual, regional and national levels.

[4]The theory demonstrates that the initial distribution of time preferences does not affect the long-run steady state as long as the support of the distribution function is bound above by the steady-state level. Therefore, if individuals with LTO traits sort into an environment where the returns from agriculture are higher, then sorting would affect the time preference in the period of transition to the steady state but would not affect the long-run steady state in the economy (Galor and Özak (2016)).

References

Ashraf, Quamrul, and Oded Galor. 2011. "Dynamics and Stagnation in the Malthusian Epoch." *American Economic Review*, 101(5): 2003–41.

Bandura, Albert, and Walter Mischel. 1965. "Modifications of Self-imposed Delay of Reward through Exposure to Live and Symbolic Models." *Journal of Personality and Social Psychology*, 2(5): 698.

Bergeron, Augustin, Raj Chetty, Michael Stepner, Sarah Abraham, Shelby Lin, Benjamin Scuderi, Nicholas Turner, and David Cutler. 2016. "The Association between Income and Life expectancy in the United States, 2001-2014." *JAMA*, 315(16): 1750–1766.

Burchardi, Konrad B, Thomas Chaney, and Tarek A Hassan. 2019. "Migrants, Ancestors, and Foreign Investments." *The Review of Economic Studies*, 86(4): 1448–1486.

Chetty, Raj, Nathaniel Hendren, Patrick Kline, and Emmanuel Saez. 2014. "Where is the Land of Opportunity? The Geography of Intergenerational Mobility in the United States." *The Quarterly Journal of Economics*, 129(4): 1553–1623.

Dalgaard, Carl-Johan, and Holger Strulik. 2015. "The Physiological Foundations of the Wealth of Nations." *Journal of Economic Growth*, 20(1): 37–73.

Desmet, Klaus, and Romain Wacziarg. 2021. "Understanding Spatial Variation in COVID-19 Across the United States." *Journal of Urban Economics*, 103332.

Dixon, Mark R, Linda J Hayes, Lisa M Binder, Sharon Manthey, Connie Sigman, and Darlene M Zdanowski. 1998. "Using a Self-control Training Procedure to Increase Appropriate Behavior." *Journal of Applied Behavior Analysis*, 31(2): 203–210.

E. Dong, H. Du, L. Gardner. 2020. "An Interactive Web-based Dashboard to Track COVID-19 in Real Time." *Lancet Infect. Dis.*, 20: 3533–534.

Galor, Oded, and Ömer Özak. 2015. "Land Productivity and Economic Development: Caloric Suitability vs. Agricultural Suitability." *Brown University Working Paper*.

Galor, Oded, and Ömer Özak. 2016. "The Agricultural Origins of Time Preference." *American Economic Review*, 106(10): 3064–3103.

Galor, Oded, Ömer Özak, and Assaf Sarid. 2016. "Geographical Origins and Economic Consequences of Language Structures." *Brown University Working Paper*.

Logue, AW, and James E Mazur. 1981. "Maintenance of Self-control Acquired through a Fading Procedure: Follow-up on Mazur andLogue (1978)." *Behaviour Analysis Letters*.

MIT Election Data and Science Lab. 2018. *County Presidential Election Returns 2000-2016.* Harvard Dataverse.

Nordhaus, WD, and X Chen. 2016. "Global Gridded Geographically Based Economic Data (G-Econ), Version 4." *NASA Socioeconomic Data and Applications Center (SEDAC)*.

Oster, Emily. 2019. "Unobservable Selection and Coefficient Stability: Theory and Evidence." *Journal of Business & Economic Statistics*, 37(2): 187–204.

Putterman, Louis, and David N Weil. 2010. "Post-1500 Population Flows and the Long-run Determinants of Economic Growth and Inequality." *The Quarterly Journal of Economics*, 125(4): 1627–1682.

Rambachan, Ashesh, and Jonathan Roth. 2019. "An Honest Approach to Parallel Trends." *Unpublished manuscript,*

Harvard University.[99].

Roodman, David, Morten Ørregaard Nielsen, James G MacKinnon, and Matthew D Webb. 2019. "Fast and wild: Bootstrap inference in Stata using boottest." *The Stata Journal*, 19(1): 4–60.

Rung, Jillian M, and Michael E Young. 2015. "Learning to Wait for More Likely or Just more: Geater Tolerance to Delays of Reward with Increasingly Longer Delays." *Journal of the Experimental Analysis of behavior*, 103(1): 108–124.

Rupasingha, Anil, Stephan J Goetz, and David Freshwater. 2006. "The Production of Social Capital in US Counties." *The journal of Socio-Economics*, 35(1): 83–101.

Vollrath, Dietrich. 2011. "The Agricultural Basis of Comparative Development." *Journal of Economic Growth*, 16(4): 343–370.
